# Evolution of diet across the animal tree of life

**DOI:** 10.1002/evl3.127

**Published:** 2019-07-09

**Authors:** Cristian Román‐Palacios, Joshua P. Scholl, John J. Wiens

**Affiliations:** ^1^ Department of Ecology and Evolutionary Biology University of Arizona Tucson Arizona 85721

**Keywords:** Animal, diet, diversification, evolution, niche conservatism, phylogeny

## Abstract

What an animal eats is a fundamental aspect of its biology, but the evolution of diet has not been studied across animal phylogeny. Here, we performed a large‐scale phylogenetic analysis to address three unresolved questions about the evolution of animal diets. (i) Are diets conserved across animal phylogeny? (ii) Does diet influence rates of species proliferation (diversification) among animal phyla? (iii) What was the ancestral diet of animals and major animal clades? We analyzed diet data for 1087 taxa, proportionally sampled among animal phyla based on the relative species richness of phyla. Our survey suggests that across animals, carnivory is most common (∼63%), herbivory less common (∼32%), and omnivory relatively rare (∼3%). Despite considerable controversy over whether ecological traits are conserved or labile, we found strong conservatism in diet over extraordinarily deep timescales. We found that diet is unrelated to rates of species diversification across animal phyla, contrasting with previous studies showing that herbivory increased diversification within some important groups (e.g., crustaceans, insects, and mammals). Finally, we estimated that the ancestor of all animals was most likely carnivorous, as were many major phyla (e.g., arthropods, molluscs, and chordates). Remarkably, our results suggest that many carnivorous species living today may have maintained this diet through a continuous series of carnivorous ancestors for >800 million years.

**Impact Summary**

What an animal eats is a fundamental part of its biology. Surprisingly, the evolution of animal diets has not been studied across all animals. Here, we analyzed diet data across an evolutionary tree of animals to address three major questions. First, are diets evolutionary conserved over time, or are they highly labile and variable among species? Whether ecological traits are evolutionarily conserved has become a major debate in evolutionary biology and ecology. Most studies have examined traits over shorter timescales, but here we test a major ecological trait over an extraordinarily deep timescale (>800 million years). Second, does diet influence rates of species proliferation, and thereby determine patterns of diversity among animal phyla? Animal phyla vary from less than five species to more than 1.2 million (i.e., arthropods). Previous studies suggested that diet (especially a herbivorous, plant‐eating diet) drives rates of species proliferation and diversity patterns in major groups of animals (e.g., mammals, insects, and crustaceans). However, this has not been tested across animal phyla. Third, what was the diet of the ancestor of all living animals, and of the major animal clades? We find three surprising results. First, we show that diet is highly conserved across animals, such that related species tend to share similar diets. Thus, we show that ecological traits can be evolutionarily conserved over incredibly deep timescales. Second, diet does not significantly influence large‐scale patterns of animal diversity, despite previous studies showing that herbivorous diet increases rates of species proliferation. Finally, we find that the ancestor of all animals was most likely carnivorous (eating other heterotrophs), as were the ancestors of many of the largest animal groups (like arthropods, chordates, and molluscs). Our results suggest that many carnivorous species living today may have inherited this trait through a series of carnivorous ancestors dating back more than 800 million years.

One of the most fundamental aspects of an animal's biology is its diet. Animals have a remarkable diversity of diets and associated lifestyles, including mammalian carnivores that pursue large and dangerous prey, insect herbivores that specialize on a few plant species, and marine invertebrates that passively filter feed on tiny organisms (Hickman et al. 2012). Yet, the evolution of animal diets remains poorly understood at the largest phylogenetic scales (e.g., among phyla). Previous large‐scale studies have suggested that food webs in natural systems are shaped (in part) by phylogenetic constraints on diet (Cattin et al. 2014) and that ecological interactions among species (e.g., predator–prey) are broadly conserved across the tree of life (Gomez et al. 2010). However, these important studies did not directly address the evolution and conservatism of trophic strategies at deep phylogenetic scales.

Here, we address three major unresolved questions about the evolution of diet across animals. First, are diets evolutionary conserved across the animal tree of life? There has been considerable debate about whether ecological niches are conserved or not, including which aspects of the niche are conserved and over what timescales (e.g., Peterson et al. 1999; Losos et al. 2003; Losos 2008; Crisp et al. 2009; Gomez et al. 2010; Wiens et al. 2010; Peterson 2011; Cattin et al. 2014; Anderson and Wiens 2017). Yet, as noted by Olalla‐Tárraga et al. (2017), this literature typically focuses on the Grinnellian niche (e.g., large‐scale climate) and not the Eltonian niche (e.g., local‐scale species interactions; terminology following Soberón 2007). Here, we provide the broadest test (so far) of conservatism in the Eltonian niche, with an analysis spanning >800 million years of evolutionary history (Fig. 1).

**Figure 1:**
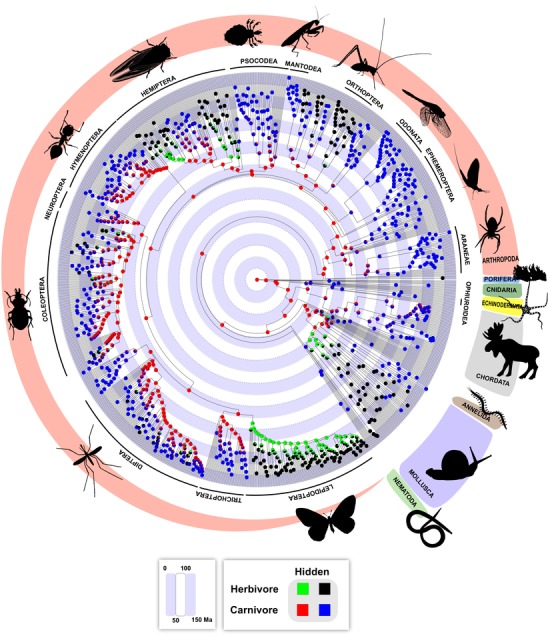
Evolution of diet across the animal tree of life, based on HiSSE. Pie diagrams at each node indicate the proportional likelihoods of each state. Nodes reconstructed as only green and/or black are unambiguously herbivorous. Red and/or blue nodes are carnivorous. Results are based on coding omnivorous and ambiguous taxa (5% of total) as carnivorous (maxcar). Results were generally similar coding them as herbivorous (Fig. S1), but the results for the maxcar strategy (under Tree I) are closer to the average results across coding strategies and topologies (Table 3). Selected phyla are shown in the outer ring of taxon labels, whereas selected subclades (e.g., insect orders) are shown in the inner ring. The full tree (Tree I; with tip labels) is in Dataset S2. Results for major nodes are also similar using alternative trees (Tables S20–S21).

Second, does diet influence rates of species diversification at broad phylogenetic scales across animal phylogeny? Previous studies have shown evidence that diet (e.g., herbivory) influences diversification within some important groups (e.g., mammals: Price et al. 2012; hexapods: Wiens et al. 2015; birds: Burin et al. 2016; and crustaceans: Poore et al. 2017). However, it remains unclear whether diet influences diversification patterns among phyla. There is striking variation in richness among animal phyla (from less than five species to more than 1.2 million) that is strongly associated with variation in diversification rates (Wiens 2015). Recent analyses suggest that most variation in diversification rates and richness among animal phyla is explained by whether phyla are predominantly nonmarine, have skeletons, and are parasites on other animals (Jezkova and Wiens 2017). However, diet itself was not included. Here, we test whether diet significantly influences large‐scale diversification patterns across animals.

Third, what was the ancestral diet of animals and what were the major shifts in diet across the animal tree of life? For example, were animals originally carnivores or herbivores? What about major phyla, such as arthropods, molluscs, and chordates? Few previous studies have explicitly addressed this topic. Vermeij and Lindberg (2000) suggested that “nonherbivory” was ancestral for animals, but focused on marine taxa, used a restricted definition of herbivory, and did not present explicit ancestral‐state reconstructions. More recent studies have commented on the possible ancestral feeding ecology of animals (using phylogenies and fossils; Sperling and Vinther 2010; Erwin et al. 2011; Sperling et al. 2013), and concluded that this ancestor was not carnivorous. However, they did not directly test whether this ancestor was more likely to be herbivorous (defined here as feeding on autotrophs) or carnivorous (i.e., feeding on heterotrophs). Although some might argue that such deep‐scale patterns can only be estimated with fossils, many relevant animal phyla do not preserve well (e.g., small, soft‐bodied taxa; Sperling 2013) and diet may be difficult to infer for many fossil taxa. Furthermore, analyzing data from extant taxa allows use of new methods that can estimate ancestral states while accounting for the possible impact of those states on diversification rates and the impact of diversification rates on ancestral‐state reconstructions (HiSSE; Beaulieu and O'Meara 2016a).

We address these three questions using a phylogenetic approach. We first assemble a dataset of 1087 carefully selected taxa with diet data from the literature (Dataset S1; all datasets and supplementary materials are available as Supporting Information and on Dryad, https://doi.org/10.5061/dryad.q2d60q3). The selected taxa have published diet data available, are represented in the time‐calibrated phylogeny assembled here (Dataset S2), and are sampled in proportion to the richness of the phyla they belong to (Table S1). We then test for phylogenetic signal in diet across the tree, as a test of niche conservatism. We next test whether diet influences diversification rates using state‐dependent speciation and extinction (HiSSE) models, and an alternative approach based on estimated net diversification rates for phyla. We then use the best‐fitting HiSSE models to estimate ancestral states and major changes in diet across animal phylogeny. We also conduct these analyses on two alternative trees, based on different assumptions about animal phylogeny and divergence times (Dunn et al. 2014; Wiens 2015). Note that HiSSE analyses can account for the potential impact of other traits on diversification besides diet, and we also perform analyses to address the potential confounding effects of marine habitat.

## Methods

Detailed methods and justification for these methods are provided in the Supplementary Methods section of the Supplementary Materials (see Supporting Information). Taxa were selected to represent each phylum, and taxa within each phylum were sampled in proportion to the richness of these phyla and also to represent major clades (when possible). Simply adding hundreds or thousands more species from certain phyla (e.g., Chordata) or groups within them (e.g., birds and mammals) would strongly bias the analyses and potentially generate misleading results for the HiSSE analyses (and others), which assume proportional sampling of taxa among clades.

## Results

### DISTRIBUTION OF DIET STATES AMONG ANIMALS

The analyzed dataset included 1087 proportionally sampled terminal taxa, including 85% arthropods, 6% mollusks, and 5% chordates (Dataset S1). Among these taxa, 63% were carnivorous, 32% herbivorous, and 3% omnivorous. The remaining taxa (2%) were ambiguous. We also estimated the frequency of diet states among species directly within each animal phylum. Across animals, we estimate the frequency of carnivory to be 59–64%, herbivory 35–39%, and omnivory 1–2% (ranges based on two alternative scenarios; Table S1). To our knowledge, these latter values represent the first direct estimates of the frequency of diet states across animal clades. Using projected richness (Table S1), given 20.9 million animal species in total, we estimate 85% are carnivorous, 14% herbivorous, and 1% omnivorous. Given 139.1 million species, we estimate 75% carnivorous, 24% herbivorous, and 1% omnivorous.

Omnivory and herbivory were collectively more common among these 28 phyla than among species (Table S1). Five phyla are predominantly (>95% of species) omnivorous, seven herbivorous, 10 carnivorous, and six with both carnivory and herbivory relatively common (>9%). Importantly, these six mixed phyla include most animal species (>94%).

### PHYLOGENETIC SIGNAL AND CONSERVATISM IN DIET

We found strong phylogenetic signal in animal diet at broad phylogenetic scales, showing that diets are evolutionarily conserved, rather than being extremely labile and varying randomly among species. The estimated lambda (Pagel 1999) was 0.79 (*P* < 0.0001; using three states), close to the maximum of 1 (Table 1). These analyses treated diet as three states (carnivory, herbivory, and omnivory). Several methods required use of only two states, and for these methods, we coded the few omnivorous and ambiguous taxa (∼5% of total) as either carnivorous (“maximum carnivory” coding, referred to as maxcar hereafter) or herbivorous (maximum herbivory coding, maxherb hereafter). Lambda values were similar using these two alternative coding methods (maxcar and maxherb; lambda = 0.84–0.85). The lambda model (including phylogenetic signal) was strongly favored over a model of random change for all coding methods (Table 1). An alternative approach, the D‐statistic (Fritz and Purvis 2010), also strongly supported a model of phylogenetic signal over a model of random change, for both binary coding methods (Table 2). Results were similar on the two alternative trees (lambda = 0.81–0.87; *P* < 0.0001), with consistently strong support for models with phylogenetic signal (Tables S2–S5).

**Table 1:** Comparison of the fit of different models for the evolution of diet, and estimated level of phylogenetic signal (lambda)

Dataset	Model	Ln‐likelihood	AICc
Three states	White‐noise	‐1059.794	2123.600
	Lambda (λ = 0.79^*^)	‐836.076	**1678.170**
Maxcar	White‐noise	‐713.628	1431.267
	Lambda (λ = 0.84^*^)	‐296.878	**599.779**
Maxherb	White‐noise	‐750.787	1505.586
	Lambda (λ = 0.85^*^)	‐345.590	**697.203**

The relative fit of two models was compared based on AICc values: a model with no phylogenetic signal (white noise model) and one with phylogenetic signal (lambda model). The best‐fitting model is boldfaced. Models were compared using the fitDiscrete function in *geiger* (Harmon et al. 2008; Pennell et al. 2014). The estimated value of lambda quantifies the level of phylogenetic signal, from 0 to 1 (maximum signal). Significant lambda values (*P* < 0.0001) are asterisked, and were tested using 1000 simulation replicates using the R package *phytools* version 0.5–65 (Revell 2012). For these analyses, we included all three states (i.e., carnivorous, herbivorous, and omnivorous; 2% taxa with ambiguous or unknown states were excluded), or coded omnivorous and ambiguous taxa (5% of all sampled taxa) as either carnivorous (maxcar) or herbivorous (maxherb). Results for alternative topologies (Trees II and III) are very similar, and are given in Tables S2 and S4.

**Table 2:** Testing for phylogenetic signal in diet using the D‐statistic

Coding strategy	Estimated D	Probability of D different from Brownian motion (strong signal)	Probability of D different from random noise (no signal)
Maxcar	‐0.483	0.996	<0.0001
Maxherb	‐0.445	0.993	<0.0001

Estimated D is scaled based on D‐values simulated under the Brownian motion model (strong phylogenetic signal) and random noise (no phylogenetic signal). Smaller values indicate stronger support for phylogenetic signal, with negative values showing that traits are highly conserved (Fritz and Purvis 2010). Probabilities (*P*‐values) indicate whether the observed D‐statistic is significantly different from 0 (Brownian motion) and from 1 (random noise). Because the D‐statistic is designed for binary data, two coding strategies were used, treating omnivorous and ambiguous taxa (5% of all sampled taxa) as either carnivorous (maxcar) or herbivorous (maxherb). Results for alternative topologies (Trees II and III) are very similar, and are given in Tables S3 and S5.

### DIET AND ANIMAL DIVERSIFICATION

We used two approaches to test the relationship between diet and diversification. Both showed little support for different diversification rates in herbivorous and carnivorous lineages. Both explicitly correct for incomplete taxon sampling in the tree. First, the best‐fitting HiSSE model for both coding strategies (labeled M24) did not support different rates of speciation and extinction in the observed diet states (Tables S6 and S7). Instead, the best‐fitting model supported different rates associated with the inferred hidden states. This model had relatively large differences in fit relative to the next best model in each case (maxcar: ΔAICc = 13.3; maxherb: ΔAICc = 6.72), indicating strong support (Table S6). Analyses of the alternative topologies also supported the M24 model, and not different diversification rates associated with different diet states (Tables S8 and S9).

Analyses of net diversification rates of phyla and their proportion of herbivorous species using phylogenetic regression showed no significant relationships (*r* ^2^ < 0.02; *P* > 0.05; Tables S10–S18), corroborating the HiSSE results. Importantly, these analyses incorporated all known species in each phylum when estimating diversification rates and diet (Tables S10–S15), not merely those species in the tree. We also performed analyses based on the projected richness (and diet) of each phylum, not merely numbers of described species. Projected numbers of animal species (total across phyla) ranged from 20.9 to 139.1 million (Table S1). None of these analyses showed a significant relationship between diet and diversification, including analyses using alternative topologies (Tables S10–S18). We also found no relationship between diversification rates and an interaction between herbivory and nonmarine habitat (Table S10–S15).

### ANCESTRAL DIET

We estimated ancestral diets across animal phylogeny using three approaches. These results are summarized in Table 3. All three methods generally supported carnivory as the most likely ancestral state for animals and most major clades (Table 3), including Bilateria, Protostomia, Deuterostomia, and the largest phyla (Arthropoda, Chordata, and Mollusca). Our primary analyses were based on HiSSE (Fig. 1), given that the complex model supported by this method had stronger support than simpler models. Using this method, support for ancestral carnivory was generally strong across coding strategies and trees (proportional likelihood > 0.87; Table 3; Tables S19–S25). Intriguingly, these reconstructions suggest that most extant carnivorous species included in our tree inherited this state through a continuous series of inferred carnivorous ancestors for >800 million years, starting with the ancestor of all animals (Fig. 1). In contrast, herbivory evolved independently in different phyla, and generally much more recently (Fig. 1).

**Table 3:** Estimated ancestral diets for key nodes across the animal tree of life

	HiSSE		corHMM		BayesTraits	
Clade	Herbivorous	Carnivorous	Herbivorous	Carnivorous	Herbivorous	Carnivorous
Root	0.067	0.933	0.089	0.911	0.096	0.904
Bilateria	0.083	0.917	0.054	0.946	0.142	0.858
Protostomia	0.087	0.913	0.081	0.919	0.062	0.938
Deuterostomia	0.098	0.902	0.047	0.953	0.077	0.910
Arthropoda	0.008	0.992	0.024	0.976	0.091	0.909
Chordata	0.086	0.914	0.039	0.961	0.088	0.912
Mollusca	0.032	0.968	0.028	0.972	0.350	0.649

Results are summarized for three different methods. For each method, we averaged the marginal (or posterior) probability of each diet across three different tree topologies (Trees I–III; Dataset S2) and two coding strategies (maxcar and maxherb). HiSSE, the preferred method, accounts for the possible impact of different diversification rates associated with different states, including both observed states (diet) and hidden states (full results in Tables S19–S21). Alternatively, corHMM allowed for hidden states in ancestral reconstructions (with different transitions rates) but did not incorporate diversification rates (full results in Tables S26–S34). Lastly, BayesTraits, the simplest method, did not include diversification rates or hidden states, and included only the observed states (full results in Tables S35–S41).

We also used a related approach (corHMM; Beaulieu et al. 2013; Beaulieu and O'Meara 2016b) that allows for more hidden states (with different transition rates) but ignores speciation and extinction rates. Reconstructions from the best‐fitting corHMM models generally supported carnivory as the most likely ancestral state for extant animals and most major clades (proportional likelihood > 0.87; Table 3; Tables S26–S34; Figs. S2–S3). However, our HiSSE results suggest that HiSSE‐type models that incorporate different speciation and extinction rates (especially for hidden states) have better fit than simpler models (like corHMM).

Finally, we analyzed even simpler likelihood models that did not incorporate diversification rates or hidden states, using BayesTraits (Pagel et al. 2004; Pagel and Meade 2006; Venditti et al. 2011). The best‐fitting models supported carnivory in the ancestor of all extant animals (Tables S35–S37; Figs. S4–S9). Comparing the fit of different root states also supported animals as ancestrally carnivorous (Table S37). On average, all BayesTraits reconstructions based on the three tree topologies and two coding strategies also supported the root and most major clades as ancestrally carnivorous (Tables 3, S35–S41). Again, these were not the primary results because they ignored diversification rates. Nevertheless, they show that these simpler models generally support our major results from HiSSE reconstructions.

## Discussion

In this study, we present the first large‐scale analysis of the evolution of animal diet. Our results show that there is strong conservatism in diet across animals, that diet appears to have little consistent impact on diversification rates, and that carnivory appears to be the ancestral state in animals (and many major clades and phyla). Remarkably, our results suggest that many carnivorous animals alive today may trace this diet through a continuous series of carnivorous ancestors stretching back for >800 million years. Below, we discuss each result in more detail.

Our results show that diets are strongly conserved among species at broad scales across the animal tree of life. As noted by Olalla‐Tárraga et al. (2017), there has been considerable debate about whether species’ ecological niches are evolutionarily conserved or not, but this literature has typically focused on the Grinnellian niche (e.g., large‐scale climate) and not the Eltonian niche (e.g., local‐scale species interactions). Here, we provide possibly the broadest test of whether a major component of the Eltonian niche is phylogenetically conserved, with an analysis spanning >800 million years (Fig. 1). Our results show strong phylogenetic signal in diet across animal phylogeny. Note that the argument that signal is uncoupled from rate (and therefore unrelated to conservatism) applies only to continuous characters, not the discrete data analyzed here (Revell et al. 2008). This pattern of strong signal and conservatism is surprising given that some authors have suggested that niches are conserved primarily over shorter timescales (e.g., Peterson 2011). Furthermore, some finer‐scale phylogenetic studies have shown mixed results regarding conservatism in diet (e.g., Olalla‐Tárraga et al. 2017). Indeed, diet shows unquestionably rapid evolution in some cases (e.g., Herrel et al. 2008). On the other hand, some previous studies have suggested that food webs are influenced by phylogenetic constraints on diet (Cattin et al. 2014) and that ecological interactions among species often show phylogenetic conservatism (Gomez et al. 2010). There is also evidence for clustering of diet types among related insect families (Rainford and Mayhew 2015), which is also consistent with our results.

There may be several explanations for the strong conservatism observed across animal phyla and for the conflicts with previous studies that suggested greater lability in diet. First, we used relatively coarse characterization of diet (e.g., carnivore, omnivore, and herbivore). Thus, two taxa could both be considered carnivores, for example, without overlapping in the species they consume. A more fine‐scaled characterization of diet might show different patterns. Nevertheless, the characterization of diet used here is typical, even for smaller‐scale studies (e.g., Price et al. 2012), and many studies are even more coarse scaled (e.g., plant feeding vs. not; Wiens et al. 2015; Poore et al. 2017). Second, analyses of different groups may simply show different patterns. However, our results show strong phylogenetic signal in diet at broad scales, regardless of results at smaller scales. Third, one factor driving conservatism in diet may be that animals cannot extract nutrients from plant cell walls themselves, and require specialized gut endosymbionts (e.g., McBee 1971; Ley et al. 2008). This may constrain the evolution of herbivory. For example, vertebrates require high body temperatures to be herbivores (seemingly due to thermal requirements of their gut microbiota), which may limit the lineages in which herbivory can evolve (e.g., Zimmerman and Tracy 1989; Espinoza et al. 2004). Fourth, our results suggest that origins of herbivory from carnivory are twice as common as gains of carnivory from herbivory (Table S42). This may reflect the more recent and widespread origins of herbivory across the tree (relative to the more ancient carnivory) or the difficulty of losing herbivory once it is attained (or regaining carnivory). This latter pattern might also contribute to phylogenetic conservatism in diet.

Our results also suggest that animals often specialize for a carnivorous or herbivorous diet, rather than being omnivores. This finding is potentially consistent with the idea that omnivory is a macroevolutionary sink, as suggested in birds (Burin et al. 2016). Specialization to carnivory or herbivory may also limit transitions between these states (i.e., few intermediates). In the food web literature, there has been debate about whether omnivory should be rare and whether it is rare in local food webs (Pimm and Lawton 1978; Yodzis 1984; McCann and Hastings 1997). Our survey supports the rarity of omnivory by focusing on clades rather than local communities, and may be the first to show (across animal phyla) this pattern of common carnivores, uncommon herbivores, and rare omnivores.

We found little significant effect of diet on diversification rates among animal phyla, even when we accounted for projected richness across phyla (Tables S1, S16–S18). This result is surprising given the evidence for faster diversification rates in herbivorous lineages in some important animal groups (e.g., mammals, insects, and crustaceans; Price et al. 2012; Wiens et al. 2015; Poore et al. 2017). Several factors may explain these contrasting results. First, our taxon sampling for the HiSSE approach may be too limited to detect positive impacts of herbivory on diversification in subclades within some phyla. However, no effect of herbivory was detected using an alternative approach that incorporated all species (Tables S10–S18). Thus, the different patterns may be more related to phylogenetic scale than limited taxon sampling. Second, increases in diversification rates associated with herbivory within some terrestrial clades might be related to the rapid diversification of angiosperms (but see Poore et al. 2017). Yet, this potentially positive effect of angiosperms on animal diversification may not apply to the largely herbivorous animal phyla (Table S10) in marine environments (e.g., Brachiopoda, Entoprocta, Hemichordata, and Kinorhyncha; Wiens 2015). Angiosperms have very limited diversity in the oceans (∼60 species; Les et al. 1997). Herbivory and marine habitats are both widespread among animal phyla and appear to be uncorrelated (Table S43). Third, analyses of diversification across animal phyla implicate parasitism (of other animals) as one of three crucial traits for explaining variation in diversification rates among phyla, along with nonmarine habitat and a skeleton (Jezkova and Wiens 2017). Thus, the positive impact of animal parasitism (carnivory) on diversification in some clades (e.g., nematodes and platyhelminths) might counterbalance the positive impacts of herbivory in others (e.g., mammals, insects, and crustaceans). This conflict may leave no strong, consistent impact of either trophic strategy when all animals are considered simultaneously. We favor this latter hypothesis overall.

Our reconstructions of diet across the animal tree of life suggested three main results: that the ancestral diet of animals was most likely carnivory, that many major animal groups were also most likely ancestrally carnivorous (e.g., arthropods, chordates, and molluscs), and that many carnivorous species extant today may trace their diet through a series of carnivorous ancestors to the ancestor of all extant animals, over 800 million years ago (Fig. 1). Few previous studies have addressed diet evolution at this deep scale. Our inference of ancestral carnivory in animals is concordant with that of Vermeij and Lindberg (2000), although those authors did not present explicit ancestral reconstructions and used a different definition of herbivory (which excluded many autotrophs). However, our results contrast with paleontological analyses that suggested that the ancestor of all animals was unlikely to be carnivorous (Erwin et al. 2011; Sperling et al. 2013). These different conclusions might reflect how dietary strategies were defined. One study (Erwin et al. 2011) suggested that carnivory was unlikely to be the ancestral state for animals (and for major animal clades). However, they defined carnivory based on how animals eat rather than on what they eat (e.g., excluding filter feeders from carnivory, regardless of what they eat). They also did not present an explicit analysis of ancestral states. Similarly, another study (Sperling et al. 2013) defined carnivory as “mobile animal‐animal interactions” rather than as feeding on heterotrophs (as we do here). We think that defining diet states based on diet alone is the more standard approach. We acknowledge that some readers may disbelieve deep‐scale ancestral reconstructions not based on fossils. However, it is difficult to directly infer diets of many fossil taxa, and some animal phyla are barely recorded in the fossil record at all (e.g., those lacking hard parts; Sperling 2013). Therefore, even though our reconstructions are not guaranteed to be correct, they may represent a particularly important line of evidence for inferring the ancestral diet of animals and many other ancient nodes. We also note that the absolute age of a given node is not necessarily relevant to whether it will be reconstructed unambiguously or correctly (e.g., Wiens 2015; Anderson and Wiens 2017). Instead, the support for a given node's reconstructions should depend more on patterns of variation in that trait among taxa near the node of interest. Finally, even if our analyses are wrong about some nodes, they still show strong support for ancient carnivory for many deep nodes (Fig. 1). Thus, regardless of the ancestral state for all animals, our results still strongly suggest that diets can be maintained over hundreds of millions of years.

In summary, we present here the first large‐scale phylogenetic analysis of the evolution of animal diets. Our results show that diet is phylogenetically conserved across animals, and that carnivory was most likely the ancestral diet of animals (and many major clades), with herbivory evolving more recently and independently across clades. Many carnivorous species living today seem to trace this diet back to this carnivorous ancestor that evolved >800 million years ago. Thus, despite considerable controversy over whether niches are conserved, our results show that a trait involved in local‐scale species interactions (Eltonian niche) can be conserved over remarkably deep timescales. Our results also show that diet does not consistently influence diversification when considered across all animals (despite strong effects in individual clades). Finally, our results suggest that carnivory is the most common dietary strategy across animals, with herbivory being less common and omnivory being relatively rare.

## CONFLICT OF INTEREST

The authors declare no competing financial interests.

Associate Editor: A. Goswami

## Supporting information


**Supplementary Material**. Supplementary Methods, Tables S1–S49, Figures S1–S9.

**Dataset S1**. Diet data for each taxon in the tree, and supporting references.

**Dataset S2**. Animal phylogenies in nexus format.

**Dataset S3**. Diet data for each taxon in the tree, with modified diet data for four phyla.
