# Supplementary material for: Evolution of diet across the animal tree of life
Source: Evol Lett. 2019 Jul 9;3(4):339–47. doi: 10.1002/evl3.127 (PMC6675143; doi:10.1002/evl3.127)

Supplementary Material

**Evolution of diet across the animal Tree of Life**

Cristian Román-Palacios^1^, Joshua P. Scholl^1^, and John J. Wiens^1^

^1^Department of Ecology and Evolutionary Biology, University of Arizona, Tucson, AZ 85721-0088, U.S.A.

**Supplementary Methods.**

**Table S1.** Summary of diet and species richness for the 28 analyzed animal phyla.

**Table S2.** Comparison of the fit of different models for the evolution of diet, and estimated level of phylogenetic signal (lambda) for Tree II.

**Table S3.** Testing for phylogenetic signal in diet using the D-statistic and Tree II.

**Table S4.** Comparison of the fit of different models for the evolution of diet, and estimated levels of phylogenetic signal (lambda) for Tree III.

**Table S5.** Testing for phylogenetic signal in diet using the D-statistic and Tree III.

**Table S6.** Comparison of the fit of different likelihood models for the evolution of animal diet (using HiSSE).

**Table S7.** Rates of speciation (λ) and extinction (μ) estimated from the best-fitting HiSSE models for each coding strategy (maxcar and maxherb).

**Table S8.** Comparison of the fit of different likelihood models for the evolution of animal diet (using HiSSE) for Tree II.

**Table S9.** Comparison of the fit of different likelihood models for the evolution of animal diet (using HiSSE) for Tree III.

**Table S10.** Estimated proportions of herbivorous, non-marine, terrestrial species and estimated net diversification rates for each of 28 animal phyla (using three different relative extinction fractions, ε) using rate estimates from Wiens (2015a), and based on Tree I.

**Table S11.** Results of PGLS analyses of the relationship between diet (proportion of herbivorous species) and net diversification rates among 28 animal phyla based on Tree I.

**Table S12.** Estimated net diversification rates for 28 animal phyla, based on Tree II.

**Table S13.** Results of PGLS analyses of the relationship between diet (proportion of herbivorous species) and net diversification rates among 28 animal phyla based on Tree II.

**Table S14.** Estimated net diversification rates for animal phyla for Tree III.

**Table S15.** Results of PGLS analyses of the relationship between diet (proportion of herbivorous species) and net diversification rates among 28 animal phyla based on Tree III.

**Table S16.** Estimated diversification rates for 28 animal phyla based on projected richness per phylum.

**Table S17.** Summary results for PGLS analyses of the relationship between diet (proportion of herbivorous species) and net diversification rates based on projected richness (lowest projections).

**Table S18.** Summary results for PGLS analyses of the relationship between diet (proportion of herbivorous species) and net diversification rates based on projected richness (largest projections).

**Table S19.** Estimated diet for key nodes of the animal Tree of Life (Tree I) based on the best-fitting HiSSE model.

**Table S20.** Estimated diet for key nodes of the animal Tree of Life based on the best-fitting HiSSE model for Tree II.

**Table S21.** Estimated diet for key nodes of the animal Tree of Life based on the best-fitting HiSSE model for Tree III.

**Table S22.** Comparison of the fit of different likelihood models for the evolution of animal diet (using HiSSE) for Trees I–III, using an alternative dataset for diet.

**Table S23.** Estimated diet for key nodes of the animal Tree of Life (Tree I) based on the best-fitting HiSSE model, using an alternative dataset for diet.

**Table S24.** Estimated diet for key nodes of the animal Tree of Life based on the best-fitting HiSSE model for Tree II, using an alternative dataset for diet.

**Table S25.** Estimated diet for key nodes of the animal Tree of Life based on the best-fitting HiSSE model for Tree III, using an alternative dataset for diet.

**Table S26.** Comparison of the fit (AICc) for different corHMM models for the evolution of diet for Tree I.

**Table S27.** Comparison of the relative fit (AICc) of three methods for estimating the initial probabilities of each state at the root of the animal tree, using the corHMM approach.

**Table S28.** Estimated ancestral states for diet for key nodes of the animal Tree of Life based on the best fitting corHMM models.

**Table S29.** Comparison of the fit (AICc) of different corHMM models for the evolution of diet for Tree II.

**Table S30.** Comparison of the relative fit (AICc) of three methods for estimating the initial probabilities of each state at the root of the animal tree, using the corHMM approach for Tree II.

**Table S31**. Estimated diet for key nodes of the animal Tree of Life based on the best-fitting corHMM model for Tree II.

**Table S32.** Comparison of the fit of different corHMM models for the evolution of diet for Tree III.

**Table S33.** Comparison of the fit of three methods for estimating the initial probabilities of each state at the root of the animal tree, using the corHMM approach for Tree III.

**Table S34.** Estimated diet for key nodes of the animal Tree of Life based on the best-fitting corHMM model for Tree III.

**Table S35.** Summary statistics for the 9 models compared for ancestral-state reconstruction of diet with BayesTraits, using Tree I.

**Table S36.** Estimated ancestral states for diet for key nodes of the animal Tree of Life (Tree I) based on BayesTraits.

**Table S37.** Comparison of the fit of different root states for animal phylogeny (Tree I) using BayesTraits.

**Table S38.** Summary statistics for the 8 models compared for ancestral-state reconstruction of diet with BayesTraits for Tree II.

**Table S39.** Estimated diet for key nodes of the animal Tree of Life based on the best fitting BayesTraits models for Tree II.

**Table S40.** Summary statistics for the 8 models compared for ancestral-state reconstruction of diet with BayesTraits for Tree III.

**Table S41.** Estimated diet for key nodes of the animal Tree of Life based on the best fitting BayesTraits models for Tree III.

**Table S42.** Transition-rate matrices for the best-fitting HiSSE model for both coding strategies.

**Table S43.** Results of PGLS analyses testing the relationship between habitat and diet among animal phyla.

**Table S44.** Comparison of the fit of different models for the evolution of diet, and estimated level of phylogenetic signal (lambda) for Tree I, using an alternative dataset.

**Table S45.** Testing for phylogenetic signal in diet using the D-statistic for Tree I, using an alternative dataset.

**Table S46.** Comparison of the fit of different models for the evolution of diet, and estimated level of phylogenetic signal (lambda) for Tree II, using an alternative dataset.

**Table S47.** Testing for phylogenetic signal in diet using the D-statistic for Tree II, using an alternative dataset.

**Table S48.** Comparison of the fit of different models for the evolution of diet, and estimated level of phylogenetic signal (lambda) for Tree III, using an alternative dataset.

**Table S49.** Testing for phylogenetic signal in diet using the D-statistic for Tree III, using an alternative dataset.

**Figure S1.** Ancestral-state reconstructions for the best-fitting model for HiSSE for the maxherb coding strategy.

**Figure S2.** Ancestral-state reconstructions for the best-fitting model for corHMM for the maxcar coding strategy.

**Figure S3.** Ancestral-state reconstructions for the best-fitting model for corHMM for the maxherb coding strategy.

**Figure S4.** Ancestral state reconstructions for the M1 model in BayesTraits using the maxherb coding strategy.

**Figure S5.** Ancestral state reconstructions for the M1 model in BayesTraits using the maxcar coding strategy.

**Figure S6.** Ancestral state reconstructions for the M2 model in BayesTraits using the maxcar coding strategy.

**Figure S7.** Ancestral state reconstructions for the M3 model in BayesTraits using the maxcar coding strategy.

**Figure S8.** Ancestral state reconstructions for the M6 model in BayesTraits using the maxcar coding strategy.

**Figure S9.** Ancestral state reconstructions for the M7 model in BayesTraits using the maxcar coding strategy.

*Supplementary Methods*

Given space constraints, the complete methods are given here rather than in the main text.

**Animal phylogeny**

We used a phylogeny (Scholl and Wiens 2016) that combined time-calibrated molecular trees from multiple sources. We modified this tree to make the sampling of terminal taxa (e.g. species, families) within phyla roughly proportional to species richness of phyla (i.e. based on described species richness) and to incorporate taxa with diet data. The overall phylogeny included 28 of 34 commonly recognized phyla (Tree 2 of Wiens 2015a). For arthropods, six taxa from the full chronogram of Wiens (2015a) were added to represent major clades (Pycnogonida, Merostomata, Chilopoda, Branchiopoda, two Malacostraca). The large-scale chordate tree was modified to include cyclostomes (following Kuraku and Kuratani 2006; Erwin et al. 2011; Wiens 2015b), a major branch of early vertebrates.

A total of 1,087 terminal taxa were sampled, including taxa represented in the time-calibrated phylogeny with diet data available. For the 28 included phyla, there was a very strong relationship (*r*^2^=0.99, *P*<0.0001) between the number of terminal taxa sampled per phylum and the number of described species per phylum (Table S1). This relationship remained strong (*r*^2^=0.86; *P*<0.0001) after excluding arthropods (which include 85% of sampled taxa). The relationship is also strong when comparing the projected richness of each phylum to the number of species included in the tree (lowest projected richness, *r*^2^=0.80; *P*<0.0001; highest projected richness, *r*^2^=0.85; *P*<0.0001; Table S1). Note that the relationship is not perfect because fully proportional sampling based on richness would exclude many phyla (17/28 have <7,000 described species, <0.5% of animal richness). Overall, we tried to balance proportional sampling with inclusion of key clades (i.e. phyla) that may be most influential in estimating ancestral states for major clades. We also tried to include key clades within relatively well-studied phyla (e.g. Arthropoda, Chordata). In other groups, we focused on taxa that were included in large-scale, time-calibrated phylogenies. For some groups, many more species with both phylogenetic and diet data could have been included (e.g. within tetrapods). However, this would have biased our proportional sampling across the tree, which would be problematic for HiSSE and other methods (i.e. potentially yielding misleading results).

In theory, it would be desirable to estimate the tree and divergence times simultaneously for all taxa across all phyla. This would facilitate incorporating uncertainty in the topology and divergence times in the comparative analyses. However, this would have been difficult here, given the many different datasets used to estimate different parts of the tree. We acknowledge that parts of animal phylogeny are controversial (review in Dunn et al. 2014), but the higher-level tree used here is relatively conservative (Dunn et al. 2014; Wiens 2015a). Nevertheless, we also performed the same analyses using two alternative trees, which generally yielded similar results. Our main results are based on Tree 2 of Wiens (2015a), referred to as Tree I hereafter, which is the most conservative in terms of both topology and clade ages (Wiens 2015a). The two alternative trees started from the two alternative phylum-level trees of Wiens (2015a; Trees 1 and 3), which incorporate different assumptions about the phylogeny and divergence times. We then added the same subtrees within phyla used for the main tree, as described above and in Scholl and Wiens (2016). We refer to these alternative trees as Trees II and III (Trees 1 and 3 of Wiens 2015a, respectively). All three trees are given in newick format in Dataset S2.

We acknowledge that our tree (and diet data) excluded six phyla. The missing phyla all have low richness (numbers from Zhang 2013) and most appear to be close to small phyla already in our tree (as per Figure 1 of Dunn et al. 2014). These include Acoelomorpha (428 species, related to Xenoturbellida), Cycliophora (2 species, sister to Entoprocta), Loricifera (30 species, related to Priapulida and Kinorhyncha), and Micrognathozoa (related to Rotifera and Gnathostomulida). The phyla Orthonectida (29 species) and Rhombozoa (or Dicyemida; 107 species) are of more uncertain placement but appear to be spiralian protostomes. None of the missing phyla appear to be close to the animal root or the largest animal phyla (Arthropoda, Chordata, Mollusca). Overall, these phyla are too small to strongly influence our estimates of diet frequencies and seem unlikely to impact our estimates of ancestral diet states for most major clades. Furthermore, their diets are somewhat unclear (e.g. for Cycliophora, Loricifera, Micrognathozoa; Kristensen 2002). However, Orthonectida and Rhombozoa are parasites on other animals (e.g. Hanelt et al. 1997; Furuya and Tsuneki 2003), and would therefore be classified as carnivores here (see below).

Note that incorporating fossils into these trees would be extremely difficult. First, diet is difficult to assess in many fossil taxa, and including taxa without diet data would be counterproductive. Second, the phylogenetic placements of many fossil taxa are highly uncertain (which would also prevent them from being included). Third, to include fossil taxa would also require having estimates of their divergence times on the molecular phylogeny (their specific placement on branches of the molecular tree). This third element is necessary but especially difficult to infer.

**Diet data**

We first obtained dietary information for each terminal taxon (e.g. species, family) in our tree from the literature (data and references in Dataset S1). We initially coded diet as a discrete character with three states (i.e. carnivore, herbivore, omnivore). When only qualitative data were available, taxa feeding primarily on autotrophs (e.g. plants, algae) were coded as herbivorous. Those consuming mostly heterotrophs were coded as carnivorous. Species consuming both heterotrophs and autotrophs (with no indication of which was the primary diet) were coded as omnivorous. When quantitative estimates were available, we used a 90% cutoff for distinguishing trophic strategies (e.g. a carnivore consumes ≥90% heterotrophs by weight or volume). Omnivores had both autotrophs and heterotrophs in quantities >10% each. The cutoff of 10% is arbitrary (as is any cutoff) but should help avoid coding species based on accidental ingestion of items outside their main diet. Moreover, most taxa were invariant for the diet types used here. Observations for captive animals were not used, unless these were the only data available for a given taxon. Taxa feeding on bacteria were coded based on other food items they ate, since it was unclear whether bacteria were autotrophs or heterotrophs (except for cyanobacteria). Fungivorous taxa were coded as carnivorous (i.e. fungi are heterotrophs), but relatively few taxa were exclusively fungivorous.

Not every terminal taxon was explicitly represented by a single species. For example, the extensive hexapod phylogeny that was used here treated each family as a terminal taxon (Rainford et al. 2014). Some hexapod families were coded based on diet data summarized in Wiens et al. (2015), but saprophagic taxa were coded as carnivorous and those feeding on wood and algae were coded as herbivorous. Most families were easily coded, since many insect orders show little variation in these diet states. Nevertheless, some families contained species with different trophic strategies (e.g. in beetles). In beetles, Wiens et al. (2015) analyzed subfamilies based on the data of Hunt et al. (2007). Here, data from different subfamilies were subsumed into the same family, coding the family based on the dietary state estimated to be present in the majority of species (based on diet and richness data for each subfamily). However, in some cases (~17 families), the diet state present in the majority of species was still unclear. In these cases, we coded the family as ambiguous. In downstream analyses, we examined the consequences of coding these ambiguous taxa as either carnivorous or herbivorous (as for omnivorous taxa). There were also four insect families present in the tree of Rainford et al. (2014) lacking diet data from Wiens et al. (2015), which were excluded. Outside of hexapods, some other terminal taxa in the tree were based on families (or other higher taxa) rather than individual species, and we coded these taxa based on published summaries of diet for these higher taxa or based on data from representative species.

When sampling species to represent higher taxa, we assumed that (all other things being equal) the diet of each species was representative of the most common diet in the higher taxa to which it belonged. However, the diet of a sampled species may not always represent the commonest state, especially when diet is highly variable within a higher taxon.

To address this possibility, we estimated the frequency of each diet within each of the 28 analyzed phyla, independently of the species sampled in the tree. The details of the data collection are given in a separate section below. We then tested whether the proportion of herbivorous species in each phylum estimated from terminal taxa in the trees was significantly related to the frequency of herbivory estimated across all species in each phylum (data for each in Table S1). These two sets of estimates were strongly related, across two different scenarios for estimating herbivory from species in the literature (see below for details; *r*^2^=0.76; *P*<0.0001). Thus, the diet frequencies among species sampled in the trees should generally reflect the overall estimate of diet across each phylum. Note that most of our tree-based analyses required that we use the terminal taxa actually sampled in the tree. However, for our clade-based analyses of herbivory and diversification, we also used the overall estimates of diet for each phylum. Furthermore, given that the relationship between the two sets of estimates for herbivory for each phylum was not perfect, we also performed a limited set of analyses in which we changed values for some terminal taxa in the tree so that they better reflected the phylum-based estimates. This yielded an almost perfect relationship between these two sets of estimates. We then performed analyses of phylogenetic signal, diversification, and ancestral reconstructions using this modified dataset (Dataset S3). The details of these analyses are described in the final section of the Supplementary Methods (after we describe how we estimated these frequencies across species in each phylum, and the differences between these two sets of estimates).

**Phylogenetic signal in diet**

The diet data were first tested for phylogenetic signal by comparing the fit of models with phylogenetic signal (based on estimated lambda values; Pagel 1999) and without (white noise). We fitted lambda and white noise models using the fitDiscrete function in the R package *geiger* version 2.0.6 (Harmon et al. 2008; Pennell et al. 2014). This function is explicitly designed to test for phylogenetic signal in discrete characters. The best-fit model was selected based on the sample-size corrected Akaike Information Criterion (AICc; Sugiura 1978). The significance of the lambda statistic was tested in the R package *phytools* version 0.5–65 (Revell 2012), using 1,000 simulation replicates.

We interpret strong phylogenetic signal as evidence of niche conservatism (e.g. Wiens et al. 2010), because phylogenetic signal shows that closely related species tend to retain similar trait values over time. Fit to an Ornstein-Uhlenbeck (OU) model could also indicate phylogenetic conservatism, even without significant signal (e.g. Wiens et al. 2010). However, implementation of the OU model is difficult for discrete data. Most importantly, the strong signal observed (see Results) suggests that a scenario of stasis without signal does not apply here. Some authors have questioned whether phylogenetic signal is relevant to niche conservatism (Losos 2008), given that signal and rate can be uncoupled (Revell et al. 2008). Yet, phylogenetic signal will reflect evolutionary rate for discrete characters (Revell et al. 2008), such as the diet data analyzed here.

As an alternative test for phylogenetic signal, we estimated the D-statistic (Fritz and Purvis 2010). This statistic was explicitly designed for evaluating signal in discrete (binary) characters. D-statistics were estimated using the R package *caper,* version 0.5.2 (Orme 2013). The D-value is the sum of changes along branches for a binary trait, with smaller values indicating fewer changes and supporting the hypothesis of trait conservatism. The estimated D-value is then compared to those generated with data simulated with a Brownian motion model (BM; strong signal) and a white noise model (WN; no signal). The estimated D-value is then scaled according to the simulated values, such that 0 indicates the conservatism expected under a BM model and 1 indicates a random distribution. Negative values indicate the trait is more conserved than expected under BM. *P*-values were calculated to determine if observed D-statistic values were significantly different from D-values simulated under the BM and WN models. Diet was treated as a binary character (see below).

**Estimating effects of diet on diversification**

We tested the effects of diet on large-scale animal diversification using two different approaches. We first compared different likelihood models that allowed for an effect of diet states on speciation and extinction rates using the Hidden State Speciation and Extinction (HiSSE) approach (Beaulieu and O’Meara 2016a, 2017). HiSSE allows for one or more unobserved (hidden) states to influence diversification, in addition to observed states. It also corrects for potential weaknesses identified with similar “BiSSE” models (Maddison and FitzJohn 2015; Rabosky and Goldberg 2015).

We first evaluated the fit of 36 HiSSE models, and two standard null models based on the main tree (Tree I). The two classical HiSSE null models constrain the two observed states to have identical speciation and extinction rates, with either two hidden states (“null two”) or four (“null four”), with speciation and extinction rates varying between the hidden states. The other 36 models correspond to different hypotheses about state-dependent diversification and transition rates between states. These models allowed for different numbers of hidden states, different transition rates between states, and different speciation and extinction rates between states (details in Table S6).

For the alternative topologies (Trees II and III), we then evaluated a subset of these 38 models. Specifically, we examined the full BiSSE model, two standard HiSSE null models, a full HiSSE, and the best fitting HiSSE model for Tree I. The best-fitting model was selected using AICc values, with an AICc difference ≥4 between the best and next-best model considered strong support for the best model (Burnham and Anderson 2002). Models were initially compared using the default method for setting initial root probabilities (i.e. “maddfitz”; FitzJohn et al. 2009).

State-dependent diversification analyses can be biased when a state is present in <10% of the terminal taxa (Davis et al. 2013). Furthermore, current implementations of the HiSSE and corHMM approaches only work with binary characters. Therefore, we used two alternative coding strategies to avoid including omnivory (3% of taxa) as a rare state: maximum herbivory (maxherb), with omnivorous and ambiguous taxa treated as herbivorous, and maximum carnivory (maxcar), with these taxa treated as carnivorous. All analyses were performed using these two coding strategies.

HiSSE includes a function that explicitly compensates for incomplete species sampling in trees. Thus, it should not necessarily be problematic that we have sampled only a small fraction of all animal species in the tree. This function assumes the overall number of species with each state can be estimated. Because taxa were randomly sampled with regards to diet and were sampled proportionally among clades across animals, we used our diet dataset (Dataset S1) to estimate the proportion of species having each diet state, and thus the sampling fraction for each state under each coding strategy (maxcar: carnivory=0.679, herbivory=0.320; maxherb: carnivory=0.630, herbivory=0.370). Importantly, because our sampling of terminal taxa was proportional to the richness of phyla, major patterns of diversity and diversification should be reflected even with limited sampling at the species level (i.e. our sampling reflects the relative age and diversity of different phyla, such as the relatively young age and high richness of arthropods, and so should capture differences in the overall diversification rates of phyla). We also confirmed that our estimates of the frequencies of different diet states across animal phyla based on our sampling in the tree were similar to the frequencies estimated across all species in each phylum (regardless of whether those species were included in the tree).

As an alternative approach to HiSSE, we also used phylogenetic generalized least-squares regression (PGLS; Martins and Hansen 1997) to test the relationship between the net diversification rate of each phylum and its proportion of herbivorous species (Table S10). We used diversification-rate estimates from the method-of-moments estimator for stem-group ages (Magallón and Sanderson 2001), using three standard values of the relative extinction fraction (epsilon, values of 0, 0.5, and 0.9). Using the crown-group estimator would be problematic given that the limited species sampling available for many phyla would likely give biased estimates of their crown ages (and hence their diversification rates; Meyer and Wiens 2018). Furthermore, stem-group estimators appear to be generally more accurate in simulations (Meyer and Wiens 2018). This approach does not require constant diversification rates within clades to accurately estimate rates (Meyer and Wiens 2018; Meyer et al. 2018) nor a positive relationship between clade age and richness (Kozak and Wiens 2016). Based on simulations, there can be strong relationships between true diversification rates (the known, simulated rates) and estimated diversification rates when clades have highly heterogeneous rates between their subclades and when rates vary strongly over time within the clade (Meyer et al. 2018), and when rates are faster in younger clades (Kozak and Wiens 2016). We estimated the proportion of herbivorous species in each phylum based on the sampled taxa (note that using carnivory instead would give identical results). Omnivorous and ambiguous taxa were treated as 50% carnivorous and 50% herbivorous when estimating phylum-level proportions. We also performed these analyses using estimated frequencies based on all species in each phylum (see below). For PGLS analyses, the phylogeny was pruned to one arbitrary taxon per phylum (all taxa will yield an identical stem-group age). PGLS was performed using *caper* version 0.5.2 (Orme 2013), with branch lengths transformed based on estimated lambda values and setting kappa and delta each to one. Overall, this approach is advantageous in incorporating all known species in each clade when estimating diversification rates, in contrast to HiSSE. This is also the same approach that revealed strong relationships between herbivory and diversification in insects (Wiens et al. 2015).

In addition to testing the relationship between diversification and diet alone, we also tested if non-marine or terrestrial habitat use was a confounding factor in this relationship (given the significant relationship between habitat use and diversification across phyla; Wiens 2015a). Specifically, we tested for effects of diet, habitat, and a diet-by-habitat interaction on diversification rates. Data on habitat use were from Wiens (2015a).

In general, we did not try to include other traits in these analyses. Note that the HiSSE analyses explicitly test whether diversification rates are influenced by the observed trait (i.e. diet) or by one or more “hidden states” associated with another trait. Furthermore, previous analyses across animal phyla show significant effects of diet (parasitism) on diversification when including other variables and without (Jezkova and Wiens 2017). Similarly, analyses across insect orders show significant effects of diet (herbivory) both before and after including other variables (Wiens et al. 2015). Overall, our HiSSE analyses explicitly account for effects of any other variables, whereas previous PGLS analyses suggest that diet can significantly impact diversification regardless of whether other traits are included. Moreover, our PGLS analyses assume that any significant relationships observed between diet and diversification are caused only by diet, making it easier to conclude that diet is the main driver of diversification. Note that including other potentially relevant variables would require species-level data for these traits for >1,000 terminal taxa, which we currently lack. Furthermore, it is not clear which variables would be relevant within each clade.

**Estimating ancestral states**

We estimated ancestral states using three approaches. First, reconstructions were primarily performed with HiSSE, using the best-fitting model(s) for each diet-coding approach (maxcar, maxherb). To assign priors to the state at the root of the tree, we used the default maddfitz approach (FitzJohn et al. 2009). Then, using the best-fitting model, we estimated the most likely state at each node across the tree (summing across hidden states to obtain values for each observed state for each node). We focused especially on the ancestor of all animals and select major clades (Bilateria, Deuterostomia, Protostomia) and the largest phyla (Arthropoda, Chordata, Mollusca). A proportional likelihood ≥0.87 was considered strong support for a given state at a given node, following the standard threshold.

As a second approach, we used corHMM, in the R package corHMM version 1.20 (Beaulieu et al. 2013; Beaulieu and O’Meara 2016b). This approach allows for more hidden states than HiSSE but does not include speciation and extinction rates. We first estimated the number of hidden states by comparing five models including 0–4 hidden rates in each observed state (for corHMM, unlike HiSSE, both observed states must have the same number of hidden states). For these comparisons, we used the default method for initial root-state probabilities (equal probabilities; “null”). After selecting the best-fitting model, we compared the fit of the null method to two other methods (“yang”: Yang 2006; “maddfitz”: FitzJohn et al. 2009). Reconstructions were then based on the best-fitting corHMM model and best-fitting method for initial root-state probabilities. Overall, HiSSE models that incorporated impacts of different diversification rates on hidden states had better fit than models (like corHMM) that ignored these effects (see Results). Therefore, the main results were based on HiSSE, not corHMM.

Finally, we performed ancestral reconstructions using even simpler models that did not estimate speciation rates, extinction rates, or hidden states. For these analyses, we used the method BayesMultistate (Pagel and Meade 2006) implemented in BayesTraits version 3.0 (Pagel et al. 2004; Venditti et al. 2011). We compared 8 different models to find the best-fitting model(s) for each coding strategy. These models involved different combinations of prior distributions (exponential, uniform), sampling methods (Markov-Chain Monte Carlo, MCMC, with and without a reversible jump; rj-MCMC), and a parameter allowing variable rates across the tree (Venditti et al. 2011).

For the analysis of each model, we ran three independent MCMC or rj-MCMC chains for 11 million generations (sampling every 1,000 generations), discarding the first 10% as burn-in. For each chain, mixing was assessed based on acceptance-rate values, with values from 20–40% considered indicative of optimal mixing (Venditti et al. 2011). Convergence was assessed based on the combined posterior distribution of the three chains by inspecting the harmonic mean over time and effective sample sizes (ESS, with ESS>200 indicating convergence; Pagel and Meade 2006). Models were compared based on AIC values, after estimating the number of parameters based on the number of analyzed states (nstates), with the number of parameters equal to nstates^2^ minus nstates (following Venditti et al. 2011). For models including variable rates, each rate was treated as an independent parameter (Venditti et al. 2011). The posterior probability for each state for six key nodes in the animal tree (see above) was estimated for the best-fitting model for each coding strategy (and those with ∆AIC<4 from the best model).

All three analyses (HiSSE, corHMM, BayesMultistate) were conducted on all three trees for both coding strategies (maxcar, maxherb). However, in the main text, we primarily focus on HiSSE results for the main tree.

**ESTIMATES OF DIET AND DIVERSIFICATION USING PROJECTED SPECIES RICHNESS OF PHYLA**

We also performed alternative analyses that addressed how the actual richness of phyla might impact these analyses. Specifically, all the analyses described above were based on numbers of described species. However, these might grossly underestimate the actual number of species in each phyla (if these species were known and described). We therefore estimated the projected richness for each phylum, and re-estimated diversification rates. We also re-estimated the frequency of herbivory, in cases where there was relevant information available to do so (see below). We then re-tested the relationship between diversification rates and herbivory using PGLS.

We used estimates for species richness for each clade following Chapman (2009), Appeltans et al. (2012), and Larsen et al. (2017). When different papers gave different estimates, we generally used the larger estimate. When a range of values was given by Appeltans et al. (2012), we used the midpoint of the range. For most phyla, we assumed that the proportion of different diets within each group will remain similar as richness increases. In general, there was too little information available to assume otherwise. However, we address modified estimates of diet for Arthropoda and Nematoda, the two phyla projected to have the highest species richness. The estimated richness and diet values are given in Table S1. Using these projections, we also estimated the total number of species with each diet state (see below).

**Arthropoda**: Larsen et al. (2017) summarized several studies that suggest that there are approximately 6.8 million terrestrial arthropod species, consisting mostly of insects (e.g. Stork et al. 2015). Following from our phylum-level estimates of diet frequencies, we assumed that 36–40% of these species are herbivorous (we used 38% here as the midpoint of these two estimates). Larsen et al. (2017) also estimated that there is approximately one mite species associated with each insect species, presumably mostly as parasites or commensals. Therefore, we assumed that there are approximately 6.8 million mite species associated with insect species, with relatively few mite species that are herbivorous. However, Larsen et al. (2017) also discussed the possibility that there could be as many as 2.7 million herbivorous mite species (although this was considered likely to be an overestimate). Appeltans et al. (2012) estimated that there may be a total of 150,000 marine crustacean species. We estimated that crustaceans include 12.2% herbivorous species (see below), as estimated among described species using data from Poore et al. (2017). However, given the small number of crustaceans relative to the total projected number of arthropod species, we did not include crustaceans in these recalculations. Overall, we estimated that there are 2.6 million herbivorous insects (and other terrestrial arthropods), 4.2 million carnivorous insects, up to 2.7 million herbivorous mites and 6.8 million carnivorous mites, for a total of 5.3 million herbivorous arthropods (32.5%) and 11.0 million carnivorous species (67.5%), and a total of 16.3 million arthropod species overall.

We also note that the number of herbivorous mite species might be overestimated, and might be considered part of the initial estimate of 6.8 million terrestrial arthropod species. Making this assumption yields 2.6 million herbivorous arthropods (19.1%) and 11.0 million carnivorous arthropods (80.9%), and a total of 13.6 million arthropod species. We used this latter number as our lower estimate of projected richness (see Table S1).

Finally, Larsen et al. (2017) also considered the possibility that there were as many as 6 morphologically cryptic arthropod species for each morphology-based species. This number was estimated from a systematic review of studies of phylogeography and cryptic species in arthropods. These cryptic species were associated with different geographic areas where each species occurred. Therefore, we simply multiplied our two previous estimates of arthropod richness by sixfold, to yield either 97.8 million arthropods (32.5% herbivorous) or 81.6 million arthropods (19.1% herbivorous). We used 97.8 million as our higher estimate of projected arthropod richness (see Table S1).

**Nematoda**: Larsen et al. (2017) estimated that there is (on average) one nematode species associated with each insect species. Therefore, we estimated that there are 6.8 million nematode species. If we assumed that each morphology-based insect species contains six cryptic species, and that each of these also had its own associated nematode species, this would yield 40.8 million nematode species. Therefore, we performed separate analyses assuming that there are 6.8 million nematode species and 40.8 million nematode species, corresponding to our analyses assuming low and high numbers of arthropod species. For these estimates, we tentatively assumed that mite species associated with insect species did not have their own nematode species (given the smaller size of insect-associated mites). We also assumed that the millions of undescribed nematode species are overwhelmingly carnivorous (given their association with insect hosts). Our other estimates of diet frequencies across nematodes (see below) also assumed that nematodes are predominantly carnivorous (97–100%). Therefore, we did not use different estimates of the frequency of herbivory for projected richness.

**Frequencies of diet states based on projected richness**: We used the estimates of diet frequencies within each phylum (see the next section) and the projected richness of each phylum (Table S1), to estimate the total frequency of each diet state across all animal species (both described and projected to exist). Using our lower projected estimate of arthropod richness, we estimated a total richness of 20.9 million animal species, and we estimate that 85% of these species are carnivorous, 14% herbivorous, and 1% omnivorous. Using our higher projected estimate of arthropod richness, we estimate a total of 139.1 million animal species, and we estimate that 75% are carnivorous, 24% herbivorous, and 1% omnivorous.

**ESTIMATING DIET FREQUENCIES ACROSS PHYLA**

In addition to estimating diet states for each phylum based on species sampled in the tree, we also estimated the frequency of each diet state (i.e. herbivory, carnivory, omnivory) in each phylum based on all known species. Species richness of clades within Annelida, Echinodermata, Hemichordata, Mollusca, Nematoda, Platyhelminthes, Porifera, Rotifera, and Tardigrada followed Roskov et al. (2019). Multiple literature sources were used for estimating species richness of taxa within Arthropods and Chordates (see references in each of these sections). The estimated frequencies of each diet within each phylum are summarized in Table S1. We give detailed justification (and references) for our estimates for each phylum below. We emphasize strongly that these frequencies are constrained by the available information for each phylum, and are merely estimates, not known values. Note that we generally summarize frequencies as proportions (0–1) rather than percentages (0–100%).

**1) Acoela**

Juvenile acoelans feed on protists, including unicellular algae such as diatoms (Brusca et al. 2016). Smaller species may continue this diet throughout their lives, whereas larger species are often predaceous, hunting minute crustaceans but also feeding on larval mollusks and other worms (Brusca et al. 2016). Therefore, acoelan species are considered omnivorous overall. The frequency of herbivory is 0, carnivory 0, and omnivory 1.

**2) Annelida**

We obtained diet estimates for groups representing 73% of the total richness within Annelida (10,527 of 14,407 species). We estimate the frequency of herbivory to be 0.194 (2,049 species), carnivory to be 0.530 (5,586 species), and omnivory to be 0.274 (2,892 species).

2.1) Polychaeta (11,764 species): The reviewed groups represented 82% of the total richness within polychaeta (9,667 of 11,764 species). The data were summarized from Fauchald and Jumars (1979). Herbivorous families are Arenicolidae (24 species), Capitellidae (201 species), Ctenodrilidae (4 species), Flabelligeridae (207 species), Paraonidae (161 species), Poecilochaetidae (31 species), Protodrilidae (39 species), Sabellariidae (130 species), Sabellidae (532 species), Scalibregmatidae (67 species), Spionidae (600 species), Tomopteridae (53 species). Carnivorous families are Amphinomidae (149 species), Aphroditidae (106 species), Dorvilleidae (201 species), Eunicidae (437 species), Euphrosinidae (59 species), Glyceridae (85 species), Goniadidae (89 species), Hesionidae (231 species), Ichthyotomidae (1 species), Lumbrineridae (302 species), Magelonidae (71 species), Nephtyidae (146 species), Oweniidae (54 species), Phyllodocidae (453 species), Polynoidae (896 species), Sigalionidae (221 species), Sphaerodoridae (115 species), Spintheridae (12 species), Syllidae (1,045 species), Tomopteridae (53 species). Omnivorous families are: Chaetopteridae (73 species), Dinophilidae (16 species), Maldanidae (271 species), Nereididae (704 species), Nerillidae (49 species), Onuphidae (341 species), Pilargiidae (34 species), Terebellidae (619 species), Trichobranchidae (83 species), Terebellidae (619 species), and Trichobranchidae (83 species). The diet is ambiguous for the following families: Cirratulidae (329 species), Opheliidae (164 species), Orbiniidae (202 species), Pectinariidae (60 species), Psammodrilidae (8 species), Serpulidae (565 species), and Trochochaetidae (13 species). In summary, we estimate that the proportions of each diet across polychaetes are 0.211 herbivorous (2,049 species), 0.489 carnivorous (4,726 species), and 0.299 omnivorous (2,892 species)

2.2) Hirudinea (680 species): This group exclusively contains animal parasites and predators (Brusca and Brusca 2005). The frequency of carnivory is 1.

2.3) Myzostomida (180 species): Myzostomids are parasitic on echinoderms (Brusca et al. 2016). The frequency of carnivory is 1.

**3) Arthropoda**

We performed two estimates of the proportion of herbivorous species in arthropods, both using estimates of species richness from Zhang (2013), with a total of 1,256,448 extant species. One utilized the estimated proportion of herbivorous species in each order of hexapods from Wiens et al. (2015) and the other used the estimated proportion from Grimaldi and Engel (2005). Using those of Wiens et al. (2015), we estimated that there are 449,686 herbivorous species (35.79%) and 806,762 carnivorous species (64.21%). Using those of Grimaldi and Engel (2005), we estimated that there are 50,4701 herbivorous species (40.17%) and 751,747 carnivorous species (59.83%). We describe how we obtained estimates for each of the four subphyla below (Chelicerata, Myriapoda, Crustacea, Hexapoda). We note that we did not explicitly include estimates of the frequency of omnivory here, but we also found relatively few taxa reported to be omnivorous at the species level (i.e. we consider omnivory to be present only when an individual species consumes both autotrophs and heterotrophs, not when a higher taxon includes both herbivores and carnivores). However, we acknowledge that further research on this topic might show higher frequencies of omnivory.

The suphylum Chelicerata includes 113,181 extant species (Zhang 2013), of which we estimate 103,377 are carnivorous and 9,804 are herbivorous. Chelicerata includes the class Pycnogonida and the class Arachnida. Pycnogonida includes 1,335 extant species (Zhang 2013), which all appear to be carnivorous (Cobb 2010). We estimate that there are 111,846 extant arachnid species, of which 102,042 are carnivorous (90.75%) and 9,804 are herbivorous (9.25%).

The estimates for arachnids were derived as follows. Opiliones includes 6,534 extant species (Zhang 2013). We considered them to be primarily carnivorous, following the summary in Halaj and Cady (2000), although some plant material is consumed. Scorpiones includes 1,988 extant species (Zhang 2013), all of which are considered to be carnivorous (Polis 1990). Solifugae includes 1,113 extant species (Zhang 2013), all of which are considered carnivorous (Walter and Proctor 2013). Pseudoscorpiones includes 3,533 extant species, which are considered carnivorous (Harvey 2002). Palpigradi includes 87 extant species, which are considered carnivorous (Nardi 2007). Ricinueli includes 61 extant species, which are considered carnivorous (Cooke 1967).

Also within Arachnida is the subclass Acari. There is a total of 54,312 species in the subclass Acari based on species numbers in Zhang (2013). Walter and Proctor (2013) indicated that only 10 of 150 families of Trombidiformes (with 25,766 species) feed on plants. Assuming that species numbers scale with family numbers, we estimate that 7% of trombidiform species are herbivorous and the rest are carnivorous (1,718 herbivorous; 24,048 carnivorous). Walter and Proctor (2013) also indicated that Scarcoptiformes (16,173) feed on a variety of items, including algae and detritus. We arbitrarily considered this group to be 50% herbivorous (8,086 herbivorous; 8,087 carnivorous). Other major mite groups appear to be carnivorous (Ixodida, ticks, 892 species; Mesostigmata: 11,419 species; Walter and Proctor 2013). We also considered Opiliocarida (35 species) and Holothyrida (27 species) to be carnivorous. In total, we estimated that there are 44,508 carnivorous mite species (82%) and 9,084 herbivorous species (18%).

The arachnid order Araneae includes 43,678 extant species (Zhang 2013), over 99% of which are considered carnivorous (Foelix 1996; Meehan et al. 2009). Amblypygi includes 163 extant species, which are considered carnivorous (Chapin and Hebets 2016). Thelyphonida includes 110 extant species (Zhang 2013), which are considered carnivorous (Grimaldi and Engel 2005). Schizomida includes 267 extant species (Zhang 2013), which are considered carnivorous **(**Grimaldi and Engel 2005).

The subphylum Myriapoda includes 11,999 extant species (Zhang 2013), of which 67% are estimated to be herbivorous. We considered the 3,112 extant species of Chilopoda to be predominantly carnivorous (Lewis 2007). We considered the 204 extant species of Symphala (Zhang 2013) to be mostly herbivorous (Minelli and Golovatch 2001). Pauropoda includes 846 extant species (Zhang 2013). They are considered to feed mostly on fungi (Minelli and Golovatch 2001), and so are classified as carnivorous here. Diplopoda includes 7,837 extant species (Zhang 2013), which are considered predominantly herbivorous (Hopkin and Read 1993).

The subphylym Crustacea is unlikely to be monophyletic, but we nevertheless used this taxon to help estimate the number of herbivorous species in arthropods overall. Zhang (2013) considered there to be 67,735 extant crustacean species. Poore et al. (2017) estimated the number of clades of herbivorous crustaceans and their species richness in their Table S1. Summing up the species richness of these clades yields 8,270 species, which is 12.2% of known crustacean species.

For subphylum Hexapoda, we first used the estimates of the proportion of herbivorous species for each order from Wiens et al. (2015), and species richness from Zhang (2013). This yields 423,571 herbivorous species out of 1,063,533 extant species (Zhang 2013), or 39.83%. We then used the estimates of the proportion of herbivorous species from Grimaldi and Engel (2005), which yields 478,586 herbivorous species out of 1063,533 extant species total, or 45.00%.

**4) Brachiopoda**

Brachiopods feed primarily on phytoplankton (Brusca and Brusca 1990; Ruppert et al. 2004). The frequency of herbivory is 1, carnivory 0, and omnivory 0.

**5) Bryozoa**

Bryozoans feed primarily on phytoplankton (Ruppert et al. 2004). The frequency of herbivory is 1, carnivory 0, and omnivory 0.

**6) Chaetognatha**

Caethonatans are exclusively carnivorous (Brusca and Brusca, 2003; Margulis and Chapman 2010; Ramel 2012; Shapiro 2012). The frequency of herbivory is 0, carnivory 1, and omnivory 0.

**7) Chordata**

We estimated the frequency of each diet in each subphylum, and then used these proportions to obtain the overall frequency for Chordata. We present two different estimates of the frequency of each diet across chordates given variation in estimates within fishes (see below). Based on the information summarized across subphyla, and using fish data from Bornstein et al. (2019), we obtained diet data for 55,508 chordate species (79% of 69,913 species total), and estimated that the frequency of herbivory is 0.207 (11,508 species), carnivory 0.592 (32,869 species), and omnivory 0.200 (11,131 species). When fish data from Fishbase (Froese and Pauly 2019) were used, we estimated that the frequency of herbivory is 0.198 (12,628 species), carnivory 0.727 (46,309 species), and omnivory 0.07 (4,690 species).

7.1) Vertebrata: Below, we estimate diet within major vertebrate groups.

7.1.1) Amphibians (8,001 species)*.* There are 8,001 species of extant amphibians, as of 31 March 2019 (Amphibiaweb 2019), of which 7,061 are anurans (frogs and toads), 728 are caudates (salamanders), and 212 are caecilians. Almost all adult amphibians are considered carnivorous (Vitt and Caldwell 2009), with only a few species that may ingest some plant matter. Based on this criterion, we consider amphibians to be carnivorous overall. However, many anuran larvae are often considered herbivorous, since they consume algae (Vitt and Caldwell 2009). Therefore, many anuran species could be considered omnivorous, considering both their adult and larval diets. However, this is complicated by several factors. First, the larvae of many anuran species are detritivores that also consume bacteria (Vitt and Caldwell 2009). In fact, the exact diets of the larvae in most anuran species have not been precisely determined. Second, many larvae are known to be carnivorous, cannibalistic, or oophagous (Vitt and Caldwell 2009). Third, many anuran species lack a tadpole stage entirely, including the members of the large clade Terraranae (Brachycephalidae: 73 species; Craugastoridae: 124; Eleutherodactylidae: 224; Strabomantidae: 699; Amphibiaweb 2019). We considered amphibians to be carnivorous overall, but we also recognize that many species might be considered omnivorous based on their larval diets.

7.1.2) Testudines (356 species)*.* There are 351 turtle species according to Uetz et al. (2018). We estimated the frequencies of herbivory, omnivory, and carnivory, based mostly on summaries of diet in Vitt and Caldwell (2009) and summaries of species richness in Uetz et al. (2018). Chelidae: considered omnivorous by Vitt and Caldwell (2009), with 58 species (Uetz et al. 2018). Pelomedusidae: considered predominantly carnivorous by Vitt and Caldwell (2009), with 27 species (Uetz et al. 2018). Podocnemididae: considered herbivorous or omnivorous by Vitt and Caldwell (2009), with 8 species (Uetz et al. 2018). These were treated as herbivorous here. Chelydridae: considered omnivorous by Vitt and Caldwell (2009), with 5 species (Uetz et al. 2018). Platysternidae: considered carnivorous by Vitt and Caldwell (2009), with 1 species (Uetz et al. 2018). Cheloniidae: this family includes the genera *Chelonia* (herbivorous; Vitt and Caldwell 2009), *Caretta* (carnivorous; Vitt and Caldwell 2009), *Eretmochelys* (carnivorous; Vitt and Caldwell 2009), *Natator* (primarily carnivorous; Ripple 1996), and *Lepidochelys* (primarily carnivorous; Ernest et al. 1984). All genera have 1 species, except for *Lepidochelys* with 2 (Uetz et al. 2018). Thus, there are 5 carnivorous species and 1 herbivorous species. Dermochelyidae: considered carnivorous by Vitt and Caldwell (2009), with 1 species (Uetz et al. 2018). Carettochelyidae: considered omnivorous by Vitt and Caldwell (2009), with 1 species (Uetz et al. 2018). Trionychidae: considered predominantly carnivorous by Vitt and Caldwell (2009), with 32 species (Uetz et al. 2018). Dermatemydidae: considered herbivorous by Vitt and Caldwell (2009), with 1 species (Uetz et al. 2018). Kinosternidae: considered carnivorous by Vitt and Caldwell (2009), with 27 species (Uetz et al. 2019). Emydidae: Stephens and Wiens (2003), estimated frequencies of 24% carnivory (*n*=14), 61% omnivory (*n*=36), and 15% herbivory (*n*=9) among 59 taxa of emydid turtles. Uetz et al. (2018) recognized 53 emydid species. Geoemydidae: an unpublished study (P. R. Stephens, J. B. Iverson, J. J. Wiens), estimated frequencies of carnivory (*n*=12; 17%), herbivory (*n*=11; 16%), and omnivory (*n*=47; 67%), among a total of 70 species (similar to the 71 used by Uetz et al. 2018). Testudinidae: considered primarily herbivorous by Vitt and Caldwell (2009), with 60 species (Uetz et al. 2018). Given these values, and assuming a total of 356 turtle species, we estimated that there were 119 carnivorous species (33.4%), 90 herbivorous species (25.3%), and 147 omnivorous species (41.3%).

7.1.3) Crocodilians (24 species)*.* All 24 crocodilian species (Uetz et al. 2018) are primarily carnivorous (Vitt and Caldwell 2009; Pough et al. 2016).

7.1.4) Squamates (10,417 species). Based on the July 2018 version of the Reptile Database (Uetz et al. 2018), we considered there to be 10,417 squamate species, of which 6,708 are lizards and 3,709 are snakes. No snakes are herbivorous or omnivorous (Vitt and Caldwell 2009), and only a few lizards are herbivorous, with the major exceptions being Iguanidae and Liolaemidae. We used diet data from Espinoza et al. (2004) to estimate the frequency of herbivory in Liolaemidae. Those authors sampled 88 species from across the group. They found 20 species that were herbivorous (22.7%), 34 that were insectivorous (38.6%), and 34 that were omnivorous (38.6%; defined as 11–50% plant matter in their diets). We alternatively considered these omnivorous species to be herbivorous and omnivorous. If these omnivorous species are considered herbivorous, then liolaemids are 61.3% herbivorous. If these omnivorous species are considered carnivorous, then liolaemids are 22.7% herbivorous. There are 307 liolaemid species according to Uetz et al. (2018). Therefore, the total number of herbivorous liolaemids is estimated to be 70 or 188. We considered all 44 species of Iguanidae to be herbivorous, following Pough et al. (2016). Although some herbivorous species are known in Agamidae, Scincidae and Teiidae (Espinoza et al. 2004), these generally consist of isolated species or genera in these large families. In total, we considered there to be roughly 1–2% herbivorous species in squamates (114 to 232). We also note that Cooper and Vitt (2002) estimated that 12% of lizard species are omnivorous. Following from that estimate, then 805 lizard species are omnivorous, and 7.7% of squamates are omnivorous overall. Therefore, we estimate that 91% of squamate species are carnivorous, 8% are omnivorous, and 1% are herbivorous.

27.1.4) Mammals (5,852 species). We estimated the frequency of different diets using data from Price et al. (2012) and Pantheria (Jones et al. 2009). In both datasets, omnivory is defined as feeding on both heterotrophs and autotrophs with no clear indication of a preference. Our dataset covers ~50% of all mammal species, except for Soricomorpha (only 30%), Rodentia (only 37%) and Didelphimorphia (only 49%). Sampling coverage within other orders was at least 50%. We summarize diet among orders as follows. Afrosoricida are carnivorous (28 of 28 species). 92% of Artiodactyla are herbivorous (168 of 183 species) and 8% are omnivorous (15 species). Within Carnivora, 54% are carnivorous (124 of 231 species), 45% omnivorous (106 species), and a single species is herbivorous. Cetacea are largely carnivorous (74 of 74 species). Most Chiroptera are carnivorous (64%, 344 of 537 species), but 25% are herbivorous (133 species) and 11% omnivorous (60 species). Cingulata are carnivorous (40%; 8 species of 20) and omnivorous (60%; 12 species). Dasyuromorphia are mainly carnivorous (86%; 44 of 51 species) and a few species are omnivorous (14%; 7 species). Dermoptera are herbivorous (2 of 2 species). We estimate that 63% of species within Didelphimorphia are omnivorous (26 of 41), 34% carnivorous (14 species) and 2% herbivorous (1 species). Diprotodontia are mostly herbivorous (76%; 83 of 108 species) and around 23% are omnivorous (25 of 108 species). Erinaceomorpha are carnivorous (72%; 13 of 18 species) and omnivorous (23%; 5 species). Hyracoidea (4 of 4 species) and Lagomorpha (48 of 48 species) are herbivorous. Macroscelidea are carnivorous (66%; 6 of 9 species) and omnivorous (33%; 3 of 9). Microbiotheria are omnivorous (1 of 1 species). Monotremata (3 of 3 species) and Notoryctemorphia (1 of 1) are carnivorous. Paucituberculata are omnivorous (60%; 3 of 5 species) and carnivorous (40%; 2 of 5). Peramelemorphia are mainly omnivorous (92%; 11 of 12 species) and a single species is herbivorous. Perissodactyla are herbivorous (14 of 14 species). Pholidota are carnivorous (4 of 4 species). Pilosa are carnivorous (44%; 4 of 9 species), herbivorous (44%) and omnivorous (12%; 1 species). Primates are mainly omnivorous (53%; 107 of 202 species) and herbivorous (47%; 95 species). Proboscideans (3 of 3 species) and Sirenians (4 of 4 species) are herbivorous. Rodents are herbivorous (53%; 421 of 781 species), omnivorous (40%; 308 species) and carnivorous (7%; 53 species). Scandentia are mainly omnivorous (86%; 12 of 14 species) and herbivorous (14%; 2 species). Soricomorpha are mainly carnivorous (79%; 86 of 109 species) and omnivorous (21%; 23 species). Tubulidentata are omnivorous (1 of 1 species). In summary, we estimated that 39% of mammal species are herbivores, 32% are carnivores, and 29% are omnivores.

7.1.5) Birds (10,356 species). Species-level diet data were retrieved from EltonTraits (Wilman et al. 2014). This dataset, including 10 different diet types, but was transformed into the three diet states used in our study (carnivory, herbivory, omnivory). We summarize diets at the ordinal level. Exclusively carnivorous orders are Ciconiiformes (19 species), Eurypygiformes (2 species), Leptosomiformes (1 species), Phaethontiformes (3 species), Podicipediformes (19 species), Procellariiformes (128 species), Sphenisciformes (18 species), Strigiformes (206 species), and Suliformes (52 species). Exclusively herbivorous orders are Coliiformes (6 species), Musophagiformes (23 species), Opisthocomiformes (1 species), and Pteroclidiformes (16 species). Finally, exclusively omnivorous orders are Cariamiformes (2 species), Casuariiformes (4 species), Otidiformes (25 species), Rheiformes (2 species), and Struthioniformes (1 species). All the remaining orders contain species with different trophic strategies. Accipitriformes are mainly carnivorous (99%; 249 of 251 species) but two species are omnivorous (1%). Caprimulgiformes are carnivorous (99%; 113 of 114 species) but a single species is herbivorous. Most falconiformes are carnivorous (98%; 63 of 64 species) and a single species is omnivorous. Within Coraciiformes, 97% of taxa are carnivorous (147 of 151 species) and 2% are omnivorous (4 species). Pelecaniformes are mostly carnivorous (96%; 102 of 106) with only 4% omnivorous (4 species). Gaviformes are manly carnivorous (80%; 4 of 5 species) but a single species is omnivorous (20%). Most Cuculiformes are carnivorous (78%; 111 of 142 species), 18% are omnivorous (25 species) and 4% are herbivorous (6 species). Charadriiformes are mostly carnivorous (73%; 269 of 369 species), but 26% are omnivorous (96 species) and 1% are herbivorous (4 species). Piciformes are mainly omnivorous (51%; 212 of 414 species), but species are also carnivorous (45%; 188 species) and herbivorous (3%; 14 species). Among Passeriformes, 49% are omnivorous (2943 of 5955 species), 43% are carnivorous (2566 species), and 8% are herbivorous (49 species). Mesitornithiformes are omnivorous (67%; 2 of 3 species) and carnivorous (33%; 1 species). Apodiformes are mainly herbivorous (74%; 331 of 450 species) but with some carnivorous (26%; 116 species) and omnivorous species (1%; 3 species). Apterygiformes are omnivorous (75%; 3 of 4 species) and carnivorous (1 species). Trogoniformes are omnivorous (70%; 30 of 43 species), carnivorous (23%; 10 species) and herbivorous (7%; 3 species). Bucerotiformes are mainly omnivorous (62%; 41 of 66), but some species are carnivores (20%: 13 species) and herbivores (18%; 12 species). Phoenicopteriformes are herbivorous (50%; 3 of 6 species), omnivorous (33%; 2 species), and carnivorous (17%; 1 species). Gruiformes are omnivorous (78%; 121 of 156 species), carnivorous (17%; 27 species) and herbivorous (5%; 8 species). Within Anseriformes, 46% of species are omnivores (73 of 160 species), 41% are herbivores (66 species), and 13% are carnivores (21 species). Galliformes are predominantly omnivorous (56%; 160 of 288 species), but 44% are herbivores (121 species) and 2% are carnivores (2 species). Psittaciformes are herbivorous (95%; 337 of 354 species) and omnivorous (5%; 17 species). Columbiformes are herbivorous (85%; 261 of 306 species) and omnivorous (14%; 44 species), but a single carnivore species is known (1%). Finally, Tinamiformes are mostly omnivorous (83%; 39 of 47 species), with 8 herbivorous species known (17%). Overall, we estimate that 45% of birds are carnivores, 39% are omnivores, and 17% are herbivores.

7.1.6) Fish (28,000 species): We estimated the frequency of each diet across families using two different datasets. Bornstein et al. (2019) summarized trophic data for reef fish (94 families, 1,545 species). FishBase (Froese and Pauly 2019) summarized species-level data for ~1,800 species in 307 families. FishBase gives several diet categories based on the percent of captured fish that a certain diet item was found in. However, the frequency of occurrence may not be a good indicator of how much a food item contributes to the diet of a given population (at least for adults). For example, phytoplankton in 50% of the examined stomachs may contribute much less to the overall diet than large polychaetes found in only 40% of the stomachs. Nevertheless, we applied our arbitrary 10% cutoff to the frequency data. If plants and animals were both above 10% for a given species we considered them an omnivore. Overall, based on Borstein et al. (2019), we estimate that 45% of fish species are carnivores, 28% omnivores, and 26% herbivores. Based on Fishbase, 93% of fish species are carnivores, 5% are omnivores, and only 3% are herbivores. Because both databases largely differ in the frequency of herbivory, we utilized both alternative estimates of the frequency of each diet in our calculations.

7.2) Urochordata (3,051 species; Ruppert et al. 2004): Although most tunicates are herbivorous, 12 benthic species from the Hexacrobylidae/Sorberacea are often classified as carnivorous (Alldredge 1982; Tatián et al. 2011). Therefore the frequency of herbivory is 0.996 (3,039 species) and carnivory is 0.04 (12 species).

7.3) Cephalochordata (28 species): Cephalochordates are filter-feeders. Although their feeding habits are largely unknown, at least one species is known to feed on phytoplankton, copepods, and detritus (Webb 1975). This is considered omnivorous here. The frequency of omnivory is 1.

**8) Cnidaria**

Cnidarians are carnivores (Likens 2009). The frequency of herbivory is 0, carnivory 1, and omnivory 0.

**9) Ctenophora**

Ctenophores are exclusively carnivorous (Brusca and Brusca 2003; Haddock 2007; Mills 2010). The frequency of herbivory is 0, carnivory 1, and omnivory 0.

**10) Echinodermata**

Based on the information summarized for each class below, we estimate that the overall frequency of herbivory within Echinodermata is 0, carnivory 0.854 (5,832 species), and omnivory 0.146 (996 species).

10.1) Crinoidea (105 species): The main food used is zooplankton (Rutman and Fishelson 1969, La Touche and West 1980; Holland et al. 1986, 1987, 1991; Brusca and Brusca 2003). The frequency of herbivory is 0, carnivory 1, and omnivory 0.

10.2) Asteroidea (1,895 species): Asteroids are primarily scavengers (feeding on decaying meat and therefore carnivores) or predators of sessile or slow-moving prey such as mollusks and barnacles (Brusca et al. 2013; Holland et al. 1991). The frequency of herbivory is 0, carnivory 1, and omnivory 0.

10.4) Ophiuroidea (2,090 species): Ophiuroids are carnivores (Hyman 1955). The frequency of herbivory is 0, carnivory 1, and omnivory 0.

10.5) Holothuroidea (1,742 species): Most species feed on dead animals (i.e. scavengers; Brusca and Brusca 2003; Waggoner 1999). The frequency of herbivory is 0, carnivory 1, and omnivory 0.

10.6) Echinoidea (996 species): Echinoids are omnivorous, grazing on both plant and animal tissues (Brusca and Brusca 1990). The frequency of herbivory is 0, carnivory 0, and omnivory 1.

**11) Entoprocta**

All entoprocts are sessile suspension feeders that usually feed on phytoplankton, as well as diatoms and algae. One species, *Loxocorone brochobola*, has nematocyst-type organs; their function is unknown but may be related to prey capture (Weise 1961; Emschermann 1993; Brusca and Brusca 2003). The frequency of herbivory is 0.995 (169 species), carnivory 0.005 (one species), and omnivory 0.

**12) Gastrotricha**

Gastrotrichians are mainly herbivorous (Brunson 1950; Bennett 1979; Brusca and Brusca, 1990; Strayer et al. 2010). The frequency of herbivory is 1, carnivory 0, and omnivory 0.

**13) Gnathostomulida**

Species primarily feed on bacteria and fungal hyphae (Barnes et al. 2003). As indicated in the Supplementary Methods, taxa feeding on bacteria are coded based on other food items, and fungi are heterotrophs. The frequency of herbivory is therefore 0, carnivory 1, and omnivory 0.

**14) Hemichordata**

Species within the most well-known and richest class within Hemichordates, the Enteropneusta (113 of 139 species), are classified as herbivorous (Brusca and Brusca 1990). At least two species, *Saccoglossus kowalevskii* and *Glandiceps hacksi*, are known to eat bacteria, diatoms, and microalgae (Grzimek et al. 2004). The frequency of herbivory is 1, carnivory 0, and omnivory 0.

**15) Kinorhyncha**

Kinorhyncha feed on unicellular algae and bacteria (Brusca et al. 2013). The frequency of herbivory is 1, carnivory 0, and omnivory 0.

**16) Mollusca**

Based on the diet summaries below for clades (classes) representing ~90% of mollusk diversity (75,337 species), we estimated the frequency of herbivory to be 0.715 (53,934 species), carnivory 0.272 (20,516), and omnivory 0.012 (897 species).

16.1) Aplacophora (320 species): Aplacophorans typically feed on cnidarians, annelids, and foraminiferans (Guralnick and Smith 1999; Todt 2013). The frequency of herbivory is 0, carnivory 1, and omnivory 0.

16.2) Polyplacophora (976 species): Except for the carnivorous genus *Placiphorella* (13 species; Horton et al. 2019), all known polyplacophorans are herbivorous (Horton et al. 2019). The frequency of herbivory is 0.987 (963 species), carnivory 0.013 (13 species), and omnivory 0.

16.3) Monoplacophora (20 species): We could find no information on their diet. Nevertheless, monoplacophorans are a species-poor lineage (i.e. <0.03% of total mollusk richness).

16.4) Cephalopoda (808 species): Cephalopods are carnivorous (Boyle and Rodhouse 2004). The frequency of herbivory is 0, carnivory 1, and omnivory 0.

16.5) Bivalvia (8,838 species): Except for a single carnivorous genus with 22 species (*Poromya*), bivalves feed on phytoplankton (Burton 2008; Horton et al. 2019). The frequency of herbivory is 0.998 (8,816 species), carnivory 0.002 (22 species), and omnivory 0.

16.6) Scaphopoda (571 species): Scaphopodans are omnivores, feeding on microscopic autotrophs and heterotrophs, including foraminiferans, bivalves, kinorhynchs, and diatoms (Brusca et al. 2016). The frequency of herbivory is 0, carnivory 0, and omnivory 1.

16.7) Caudofoveata (141 species): Caudofoveates feed on foraminiferans (Guralnick and Smith 1999), which are considered heterotrophs. The frequency of herbivory is 0, carnivory 1, and omnivory 0.

16.8) Gastropoda: We review gastropod diet based on the major orders. Higher-level taxonomy follows Jörger et al. (2010). Based on the information below for clades comprising 97% of gastropod diversity (63,521 of ~65,000 species), we estimate that the frequency of herbivory within gastropods is 0.692 (43,983 species), carnivory 0.302 (19,212), and omnivory 0.005 (326 species).

16.8.1) Panpulmonata (30,194 species): We reviewed diet within the major clades within Panpulmonata (Jörger et al. 2010). Sacoglossa (284 species) feed on algal cytoplasms (Heller 2015) and so are considered herbivorous. Glacidorboidea (20 species) are predatory carnivores (Strong et al. 2007). Pyramidelloidea (400 species) are ectoparasites of bivalves and polychaetes (Barnes 1982) and so are considered carnivorous. Hygrophila species (8,538 species) graze on plant matter and algae (Heller 2015) and so are considered herbivorous. However, a few species within Hygrophila are scavengers (Barnes 1982). Most Stylommatophora (20,500 species) are herbivorous (Barnes 1982), and a few carnivore species within the same group are classified under the Testacellidae (5 species) and Systrophiidae (44 species; Hausdorf 1998). Within Systellommatophora (137 species), Rathouisiidae (13 species) prey on other slugs and snails (Barnes 1982), Onchidiidae (86 species) feed on algae and bacteria (Rudman 1999), and Veronicellidae are herbivorous (South 2012). Acochlidiacea (46 species) are mainly herbivorous. We did not find information on the diet of Siphonarioidea (8 species), Amphiboloidea (15 species), Ellobioidea (224 species), Otinoidea (7 species), and *Trimusculus* (15 species). Species within the genus *Arion* are omnivorous (40 species). The frequency of herbivory is 0.678 (20,537 species), carnivory 0.321 (9,716 species), and omnivory 0.001 (40 species).

16.8.2) Euopisthobranchia (1,043 species): Species within Euopisthobranchia are classified as either carnivorous or herbivorous (Lobo-da-cunha et al. 2015). Carnivorous species belong to Cephalaspidea (600 species) and Gymnosomata (27 species; Jörger et al. 2010). The frequency of herbivory is 0.399 (416 species), carnivory 0.601 (627 species), and omnivory 0.

16.8.3) Nudipleura (3,069 species): All known Nudibranchia (2,406 species), the largest group within Nudipleura, are carnivorous (Valdés and Adams 2005). The remaining species in Nudipleura, classified within the Pleurobranchomorpha feed on ascidians (Thompson 1988). The frequency of herbivory is 0, carnivory 1, and omnivory 0.

16.8.4) Caenogastropoda (25,609 species): Most caenogastropods are herbivorous (Takano and Kano 2014), but at least 22 genera and four families are carnivorous (Bouchet and Rocroi 2005; Takano and Kano 2014). Carnivorous genera (not including those within the previously listed families; species richness from Roskov et al. 2019) include *Cerithium* (65 species), *Melanopsis* (38 species), *Planaxis* (7 species), *Terebra* (152 species), *Eburna* (3 species), *Buccinum* (129 species), *Harpa* (15 species), *Dolium* (100 species), *Cassidaria* (17 species), *Cassis* (11 species), *Ricinula* (2 species), *Cancellaria* (16 species), *Purpura* (3 species), *Concholepas* (1 species), *Pleurotoma* (1,052 species), *Rostellaria* (32 species), *Fusus* (11 species), *Fasciolaria* (6 species), *Turbinella* (8 species), *Columbella* (18 species), *Murex* (35 species), and *Ranella* (4 species). Additionally, family Eulimidae (706 species) feed on echinoderms, Pyramidellidae (3,038 species) prey on molluscs and polychaetes, and Epitoniidae (496 species), Coralliophilinae (250 species), and Pediculariinae (20 species) feed on anthozoans. The frequency of herbivory is 0.775 (19,870 species), carnivory 0.224 (5,739 species), and omnivory 0.

16.8.5) Neritimorpha (350 species): The Neritidae, comprising 74% of extant species within Neritimorpha (260 species) are probably the most well-studied group within Neritimorpha. Neritidae feed on diatoms, algae and the leaves of sea grasses (Heller 2015). The frequency of herbivory is 1, carnivory 0, and omnivory 0.

16.8.6) Vetigastropoda (3,700 species): We review diet within vetigastropod subfamilies. Fissurelloidea (135 species) are omnivorous and feed on sessile organisms and plant detritus (Heller 2015). Lepetodriloidea (26 species) and Neomphaloidea (50 species) are algae grazers that also feed on bacteria (Heller 2015). We consider them herbivorous here. Pleurotomarioidea (55 species) are carnivorous and feed on sponges, crinoids, and soft corals (Heller 2015). Haliotidoidea (55 species) are omnivorous, feeding on algae and sponges (Heller 2015). Most Trochoidea (2,088) are herbivorous, but two *Callistoma* species feed on invertebrates and several *Solariella* prey on foraminiferans (Heller 2015). We did not find information on the diet of three subfamilies: Angarioidea, Scissurelloidea, and Seguenzioidea. Out of the total 2409 species with diet (65% of the total richness), we estimate that the frequency of herbivory within the group is 0.899 (2,164 species), carnivory 0.002 (55 species), and omnivory 0.08 (190 species).

16.8.7) Patellogastropoda (104 species): Patellogastropods are herbivorous grazers (Heller 2015). The frequency of herbivory is 1, carnivory 0, and omnivory 0.

**17) Nematoda**

The following groups are mostly herbivorous (Yeates 1993): Rhabditida (163 species), Dorylaimida (259 species), and Triplonchida (6 species). Additionally, the following families contain at least one herbivorous species (Roskov et al. 2019; Horton et al. 2019): Anguinidae (2 species), Neotylenchidae (33 species), Psilenchidae (2 species), Rotylenchulidae (1 species), Tylenchidae (38 species), Aphelenchidae (30 species), Aphelenchoididae (1 species), and Belondiridae (13 species). Omnivorous groups (Yeates 1993) are: Aphelenchoides (150 species), Onchulidae (1 species), Actinolaimidae (5 species), Aporcelaimidae (34 species), Dorylaimidae (428 species), and Eudorylaimus (52 species). Because most species of Nematoda are considered to be carnivorous (Brusca and Brusca 2003), we assumed that all the remaining species apart from the herbivorous and omnivorous groups listed above are carnivorous. Based on the information summarized above, among the total of 25,033 described nematode species, the frequency of herbivory is 0.022 (548 species), carnivory 0.951 (23,815 species), and omnivory 0.027 (670 species).

**18) Nematomorpha**

Nematomorphans feed only during the parasitic stage. They parasitize arthropods (Brusca et al. 2013). The frequency of herbivory is 0, carnivory 1, and omnivory 0.

**19) Nemertea**

Most ribbon worms are predators (invertebrates and fish) or scavengers (decaying animal matter). A single monotypic genus (*Malacobdella*) is suspected to feed on phytoplankton captured from their host’s feeding and gas exchange currents (Brusca et al. 2013). The frequency of herbivory is 0.001 (one species), carnivory 0.999 (1,357 species), and omnivory 0.

**20) Onychophora**

Onychophorans prey on small invertebrates such as snails, worms, termites, and other insects (Brusca et al. 2013). The frequency of herbivory is 0, carnivory 1, and omnivory 0.

**21) Phoronida**

Phoronids are omnivores, usually feeding on bacteria, phytoplankton, diatoms, flagellates, peridinians, small invertebrate larvae, and detritus (Emig 1982). Semi-digested remnants of diatoms and shells of dinoflagellates are often encountered in the lumen of the intestine and in the stomach cells of phoronids (Temereva and Malakov 2010). The frequency of herbivory is 0, carnivory 0, and omnivory 1.

**22) Placozoa**

In laboratory settings, placozoans are known to feed on flagellated chromists (*Cryptomonas*) and chlorophytes (*Chlorella*), other algae, the nauplii of *Artemia* species, and commercial fish food (Collins 2000; Miller and Ball 2005; Pearse and Voigt 2007). Their diet in the wild is unclear, but given that they consume both autotrophs and heterotrophs in the laboratory, we assume that they are omnivores. The frequency of herbivory is 0, carnivory 0, and omnivory 1.

**23) Platyhelminthes**

Most platyhelminth species are generally considered to be carnivorous predators or scavengers, feeding on animal matter, bacteria, or fungi (Brusca and Brusca 2003). For example, Turbellaria (5,706 species) are carnivorous, preying on small invertebrates or protozoans, or scavenging on dead animals (Ruppert and Barnes 2004). However, at least eight turbellarian species are symbiotic with algae (Douglas 1988, 1987; Rumpho et al. 2011). Trematoda (5,975 species), Cestoda (1,940 species), and Monogenea (4,995 species) are parasites of animals (Walker and Anderson 2001). Among these four groups, the frequency of herbivory is <0.001 (8 species), carnivory 0.999 (18,616 species), and omnivory 0. The overall number of described species is 29,275 (Zhang 2013), but platyhelminths are generally considered to be carnivorous based on our criteria (Brusca and Brusca 2003).

**24) Porifera**

Sponges are usually classified as omnivorous (Brusca and Brusca 1990). They ingest particles including bacteria, protists, and micro-algae (Reiswig 1971). However, it has been shown recently that a carnivorous macrophage diet evolved in the deep sea poriferan taxon Cladorhizidae (Demospongiae; 177 species; Vacelet and Boury-Esnault 1995, 1996; Vacelet and Duport 2004; Horton et al. 2019). Based on the 8,659 known poriferan species, the frequency of herbivory is 0, carnivory 0.020 (177 species), and omnivory 0.979 (8,482 species).

**25) Priapulida**

Priapulids feed on soft-bodied invertebrates (e.g. polychaetes) and algae (Trott 1998; Brusca et al. 2013). The frequency of herbivory is 0, carnivory 0, and omnivory 1.

**26) Rotifera**

We review diet within three different groups within Rotifera (including Acanthocephala). The largest group within Rotifera, the Monogononta (~1,570 species), includes herbivorous species that feed on detritus, bacteria, and algae (Segers 2004, 2007). Acanthocephala (1,130 species) are all parasites of animals (Brusca and Brusca 2005). Finally, except for a single species, Bdelloidea (450 species) are herbivorous and feed on bacteria and yeast (Ricci et al. 2001; Majdi et al. 2012; Mialet et al. 2013). Based the total 3,150 rotifer species, the frequency of herbivory is 0.498 (1,570 species), carnivory 0.502 (1,580 species), and omnivory 0.

**27) Tardigrada**

Most tardigrades are phytophagous or feed on bacteria. Only a single carnivore species is known (Morgan 1977). Given the total species richness within Tardigrada (1,667 species), the frequency of herbivory is 0.999 (1,166 species), carnivory <0.001 (one species), and omnivory 0.

**28) Xenoturbellida**

Gut contents of *Xenoturbella* suggest that they feed primarily on bivalves (Brusca et al. 2013). The frequency of herbivory is 0, carnivory 1, and omnivory 0.

**COMPARING TREE-BASED AND LITERATURE-BASED ESTIMATES OF DIET FOR EACH PHYLUM**

We used two approaches for estimating the frequency of diet within each of the 28 analyzed animal phyla. First, we collected information on the diet of each of the 1,087 terminal taxa in our tree (Dataset S1) and then estimated the frequency of each diet state within each phylum based on their frequency among the sampled terminal taxa (Table S1). Second, we estimated the overall frequency of each diet state within each phylum (Table S1) based on the available literature for each phylum (see section above). For simplicity, we summarize both estimates as the frequency of herbivory (Table S1). A linear regression analysis showed that these estimates of the frequency of herbivory in each phylum are broadly similar to each other across phyla (*r*^2^=0.76; *P*<0.0001, under both herbivory scenarios in Table S1; see above). Below we compare these estimates on a phylum-by-phylum basis.

For 24 out of the 28 analyzed phyla, the difference between these estimates for each phylum was less than 30%. These phyla are Annelida (difference between estimates=27%), Arthropoda (difference between estimates for scenario 1 is 1.7%, and for scenario 2 is 6%), Brachiopoda (0%), Chaetognatha (0%), Chordata (scenario 1=4%, scenario 2=15%), Cnidaria (0%), Ctenophora (0%), Echinodermata (2.7%), Entoprocta (0.5%), Gastrotricha (0%), Gnathostomulida (0%), Hemichordata (25%), Kinorhyncha (0%), Mollusca (11%), Nematoda (3.5%), Nematomorpha (0%), Nemertea (0.1%), Onychophora (0%), Placozoa (0%), Platyhelminthes (0.1%), Porifera (1%), Priapulida (0%), Tardigrada (0.1%), and Xenoturbellida (0%). The difference was larger than 30% for only four phyla: Acoela (50%), Bryozoa (50%), Phoronida (50%), and Rotifera (50%). We address these four phyla below.

**ALTERNATIVE ANALYSES BASED ON MODIFIED DIET STATES**

We then performed alternative analyses that accounted for the largest differences in estimated diet frequencies between datasets. We first estimated phylogenetic signal across the tree (as in Tables 1 and 2 of the main text). We then focused on our analyses of diversification and ancestral states using HiSSE (note that our other analyses of ancestral states are based on simpler models that do not fit as well as HiSSE). Our clade-based PGLS analyses of diversification and diet (described above) already incorporated both sets of frequencies. For this analysis, we first modified the tree-based dataset (Dataset S1) so the phylum-level frequencies of herbivory matched the ones estimated across all species in each phyla. We focused on the four phyla that showed differences >30% (Acoela, Bryozoa, Phoronida, Rotifera). Each is represented by a single terminal taxon in our tree. Specifically, we made Acoela omnivorous, Bryozoa herbivororous, and Phoronida omnivorous. The single rotifer species in tree was then treated as ambiguous, since carnivory and herbivory are at roughly equal frequencies in Rotifera (Table S1). We then re-tested the relationship between the frequency of herbivory in each phylum estimated based on species in the tree (modified to reflect these four changes) and the frequencies estimated across all species in each phylum (Table S1). Importantly, these four changes were enough to yield very strong relationships between datasets (scenario 1, *r^2^=*0.960, *P<*0.0001; scenario 2, *r^2^=*0.965, *P<*0.0001). The alternative version of Dataset S1, with these four changes, is provided as Dataset S3. Note that this modified dataset is almost identical to the original dataset, since 99.6% of the data cells are unchanged.

Using this modified dataset, we first analyzed patterns of phylogenetic signal in the diet data, using all three coding strategies (three states, maxcar, maxherb) and the three topologies (Trees I, II, and III). We also applied the D-statistic to the binary data (maxcar, maxherb). The results (Tables S44–S49) were very similar to those from the main dataset, and strongly supported the presence of phylogenetic signal in the data (λ=0.80–0.87, *P<*0.0001) and models of phylogenetic signal over white noise.

We then repeated the HiSSE analyses for all three analyzed topologies (Trees, I II, and III) under both coding strategies (maxherb and maxcar). For each tree and coding strategy, we compared the fit of the full BiSSE model, two standard HiSSE null models, a full HiSSE model, and the best-fitting HiSSE model from the original analyses (M24). These results are given in Table S22. We then performed marginal reconstruction analyses on each tree based on the model with the best fit and extracted the marginal probability of each state for each of several key nodes of the animal Tree of Life. These results are given in Tables S23–S25.

In short, the best-fitting model for each tree and coding method (Table S22) was the same as in the original analyses (M24). This model does not support differences in diversification rates between diet states. Furthermore, ancestral reconstructions based on this model and the modified dataset were very similar to the original analyses. Specifically, the ancestral diet for the root of animals and for major clades (Bilateria, Protostomia, Deuterostomia) and the largest phyla (Arthropoda, Chordata, Mollusca) was supported as carnivory. The support for ancestral carnivory was generally strong for each node (probability >0.87), especially when averaged across coding methods and trees for each node (all nodes >0.90).

**REFERENCES (not in the main text)**

Alldredge, A. L., and L. P. Madin. 1982. Pelagic tunicates: unique herbivores in the marine plankton. Bioscience 32:655–663.

Appeltans, W., et al. 2012. The magnitude of global marine species diversity. Curr. Biol. 22:2189–2202.

AmphibiaWeb. 2019. <[https://amphibiaweb.org](https://amphibiaweb.org/)> University of California, Berkeley, CA, USA.

Barnes, R. F. K, R. S. Fox, and R. D. Barnes. 2003. Invertebrate Zoology. Sinauer Associates, Sunderland.

Barnes, R. F. K. 1982. Invertebrate Zoology. Sinauer Associates, Sunderland.

Bennett L. W. 1979. Experimental analysis of the trophic ecology of *Lepidodermella squamata* (Gastrotricha: Chaetonotida) in mixed culture. Trans. Am. Microsc. Soc. 98:254–260.

Borstein, S. R., J. A. Fordyce, B. C. O’Meara, P. C. Wainwright, and M. D. McGee. 2019. Reef fish functional traits evolve fastest at trophic extremes. Nat. Ecol. Evol. 3:191.

Bouchet, P, and J.-P. Rocroi. 2005. Classification and nomenclator of gastropod families. Malacologia 47:1–2.

Boyle, P., and P. Rodhouse. 2004. Cephalopods: ecology and fisheries. Blackwell, New Jersey.

Brunson, R. B. 1950. An introduction to the taxonomy of the Gastrotricha with a study of eighteen species from Michigan. Trans. Am. Microsc. Soc. 69:325-352.

Brusca, R. C., and G. J. Brusca. 1990. Invertebrates. Sinauer Associates, Sunderland, MA.

Brusca, R. C., and G. J. Brusca. 2003. Invertebrates. Sinauer Associates, Sunderland, MA.

Brusca, R. C., W. Moore, and M. Schuster. 2016. Invertebrates. Sinauer Associates, Sunderland, MA.

Burnham, K. P., and D. R. Anderson. 2002. Model selection and multimodel inference. Springer, NY.

Cameron, C. 2002. Particle retention and flow in the pharynx of the enteropneust worm *Harrimania planktophilus*: The filter-feeding pharynx may have evolved before the chordates. Biol. Bull. 202:192–200.

Chapin, K. J., and E. A. Hebets. 2016. Behavioral ecology of amblypygids. J. Arach. 44:1–14.

Chapman, A. D. 2009. Numbers of living species in Australia and the world. Report for the Australian Biological Resources Study, Australia.

Cobb, M. 2010. Pycnogonids. Curr. Biol. 20:R591–R593.

Collins, A. 2000. Introduction to Placozoa. Available at: <https://ucmp.berkeley.edu/phyla/placozoa/placozoa.html>

Cooke, J. A. L. 1967. Observations on the biology of Ricinulei (Arachnida) with descriptions of two new species of *Cryptocellus*. J. Zool. 151:31–42.

Cooper, W. E., Jr., and L. J. Vitt. 2002. Distribution, extent, and evolution of plant consumption by lizards. J. Zool. 257:487–517.

Davis, M. P., P. E. Midford, and W. P Maddison. 2013. Exploring power and parameter estimation of the BiSSE method for analyzing species diversification. BMC Evol. Biol. 13:38.

Douglas, A. E. 1987. Experimental studies on symbiotic *Chlorella* in the neorhabdocoel turbellaria *Dalyellia viridis* and *Typhloplana viridata*. Br. Phycol. J. 22:157–161.

Douglas, A. E. 1988. Alga-invertebrate symbiosis. In: Biochemistry of the algae and cyanobacteria (Rogers, L. J., and J. R. Gallon, eds.). Oxford Scientific Publisher, Oxford.

Emig, C. C. 1982. The biology of Phoronida. Adv. Mar. Biol. 19:1–89.

Emschermann, P. 1993. On Antarctic Entoprocta: Nematocyst-like organs in a loxosomatid, adaptive developmental strategies, host specificity, and bipolar occurrence of species. Biol. Bull. 184:153–185.

Ernst, C. H., R. W. Barbour, and J. E. Lovich. 1994. Turtles of the United States and Canada. Smithsonian Institution Press, Washington.

Fauchald, K., and P. A. Jumars. 1979. The diet of worms: a study of polychaete feeding guilds. Oceanogr. Mar. Biol. 17:193–284.

FitzJohn, R.G., W. P. Maddison, and S. P. Otto. 2009. Estimating trait-dependent speciation and extinction rates from incompletely resolved phylogenies. Syst. Biol. 58:595–611.

Foelix, R. F. 1996. Biology of spiders. Oxford University Press, New York.

Froese, R. and D. Pauly. 2019. FishBase. Available at: [www.fishbase.org](http://www.fishbase.org).

Furuya, H., and K. Tsuneki. 2003. Biology of dicyemid mesozoans. Zool. Scr. 20:519–532.

Grzimek, S. F. C., D. A. Thoney, N. Schlager, and M. Hutchins. 2004. Grzimek's animal life encyclopedia. Thomson/Gale, Detroit.

Guralnick, R., and K. Smith. 1999. Historical and biomechanical analysis of integration and dissociation in molluscan feeding, with special emphasis on the true limpets (Patellogastropoda: Gastropoda). J. Morph. 24:175–195.

Haddock, S. 2007. Comparative feeding behavior of planktonic ctenophores. Integr. Comp. Biol. 47:847–853.

Halaj, J., and A. B. Cady. 2000. Diet composition and significance of earthworms as food of harvestmen (Arachnida: Opiliones). Am. Midl. Nat. 143:487–491.

Hanelt, B. D. Van Schyndel, C. M. Adema, L. A. Lewis, and E. S. Loker. 1996. The phylogenetic position of *Rhopalura ophiocomae* (Orthonectida) based on 18S ribosomal DNA sequence analysis. Mol. Biol. Evol. 13:1187–1191.

Harvey, M. S. 2002. The neglected cousins: what do we know about the smaller arachnid orders? J. Arach. 30:357–372.

Hausdorf, B. 1998. Phylogeny of the *Limacoidea* sensu lato (Gastropoda: Stylommatophora). J. Molluscan Stud. 64:35–66

Heller, J. 2015. Sea snails - A natural history. Springer International Publishing, Switzerland.

Holland, N. D., J. C. Grimmer, and K. Wiegmann. 1991. The structure of a sea lily *Calamoerinus* *diomedae*, with special reference to the articulations, skeletal microstructure, symbiotic bacteria, axial organs, and stalk tissues (Crinoida, Millericrinida). Zoomorphology 111:115–132

Holland, N. D., J. R. Strickler, and A. B. Leonard. 1986. Particle interception, transport and rejection by the feather star *Oligometra serripinna* (Echinodermata: Crinoidea), studied by frame analysis of videotapes. Mar. Biol. 93:111–126

Holland, N. D., A. B. Leonard, and J. R. Strickler. 1987. Upstream and downstream feeding by Oligometra serripinna (Echinodermata: Crinoidea) under surge conditions. Mar. Biol. Lab. 173:552–556

Hopkin, S. P., and H. J. Read. 1992. The biology of millipedes. Oxford University Press, Oxford, UK.

Horton, T. et al. 2019. World Register of Marine Species. Available at: <http://www.marinespecies.org>

Hyman, L. H. 1955. The invertebrates. McGraw-Hill, New York.

Jones, K. E., et al. 2009. PanTHERIA: a species‐level database of life history, ecology, and geography of extant and recently extinct mammals. Ecology 90:2648.

Jörger K. M., I. Stöger, Y. Kano, H. Fukuda, T. Knebelsberger, and M. Schrödl. 2010. On the origin of Acochlidia and other enigmatic euthyneuran gastropods, with implications for the systematics of Heterobranchia. BMC Evol. Biol. 10:323.

Kozak, K. H., and J. J. Wiens. 2016. Testing the relationships between diversification, species richness, and trait evolution. Syst. Biol. 5:975–988.

Kristensen, R. M. 2002. An introduction to Loricifera, Cycliophora, and Micrognathozoa. Integr. Comp. Biol. 42:641–651.

Kuraku, S., and S. Kuratani. 2006. Time scale for cyclostome evolution inferred with a phylogenetic diagnosis of hagfish and lamprey cDNA sequences. Zool. Sci. 23:1053–1064.

La Touche, R. W., and A. B. West. 1980. Observations on the food of *Antedon bifida* (Echinodermata: Crinoidea). Mar. Biol. 60:39–46.

Larsen, B. B., E. C. Miller, M. K. Rhodes, and J. J. Wiens. 2017. Inordinate fondness multiplied and redistributed: the number of species on Earth and the new Pie of Life. Q. Rev. Biol. 92:229–265.

Lewis, J. G. E. 2007. The biology of centipedes*.* Cambridge University Press, London*.*

Likens, G. E. 2009. Encyclopedia of inland waters. Elsevier Academic Press, London.

Lobo-da-Cunha, A., Â.Alves, E. Oliveira, and G. Calado. 2015. Comparative study of salivary glands in carnivorous and herbivorous cephalaspideans (Gastropoda: Euopisthobranchia). J. Molluscan Stud. 82:43–54.

Maddison, W. P., and R. G. FitzJohn. 2015. The unsolved challenge to phylogenetic correlation tests for categorical characters. Syst. Biol. 64:127–136.

Magallón, S., and M. J. Sanderson. 2001. Absolute diversification rates in angiosperm clades. Evolution 55:1762–1780.

Majdi, N., et al. 2012. The relationship between epilithic biofilm stability and its associated meiofauna under two patterns of flood disturbance. Freshw. Sci. 31:38–50.

Margulis, L., and M. J. Chapman. 2009. Kingdoms and domains: An illustrated guide to the phyla of life on Earth. Academic Press, London.

Meehan, C. J., E. J. Olson, M. W. Reudink, T. K. Kyser, and R. L. Curry. 2009. Herbivory in a spider through exploitation of an ant-plant mutualism. Curr. Biol. 18:R892–893.

Meyer, A. L. S., and J. J. Wiens. 2018. Estimating diversification rates for higher taxa: BAMM can give problematic estimates of rates and rate shifts. Evolution 72:39-53.

Meyer, A. L. S., C. Román-Palacios, and J. J. Wiens. 2018. BAMM gives misleading rate estimates in simulated and empirical datasets. Evolution 72:2257–2266.

Mialet, B., N. Majdi, M. Tackx, F. Azémar, and E. Buffan-Dubau. 2013. Selective feeding of bdelloid rotifers in river biofilms. PLoS One 8:e75352.

Miller, D., and E. Ball. 2005. Animal evolution: The enigmatic phylum Placozoa revisited. Curr. Biol. 15:R26–R28.

Mills, C. 2010. Ctenophores. Available at: <http://faculty.washington.edu/cemills/Ctenophores.html>.

Minelli, A., and S. I. Golovatch. 2001. Myriapods. In: Encyclopedia of Biodiversity (S. A. Levin ed.). Academic Press, London.

Morgan, C. I. 1977. Population dynamics of two species of Tardigrada, *Macrobiotus hufelandii* (Schultze) and *Echiniscus* (*Echiniscus*) *testudo* (Doyere), in roof moss from Swansea. J. Anim. Ecol. 46:263–279.

Nardi, J. B. 2007. Life in the soil: a guide for naturalists and gardeners. University of Chicago Press, Chicago.

Orme, C. D. L. 2013. caper: Comparative analyses of phylogenetics and evolution in R. R package version 0.5.2. Available at: <http://cran.r-project.org/web/packages/caper/index.html>.

Pearse, V., and O. Voigt. 2007. Field biology of placozoans (*Trichoplax*): Distribution, diversity, biotic interactions. Integr. Comp. Biol. 47:677–692.

Polis, G. A. 1990. The biology of scorpions. Stanford University Press, Stanford.

Pough, F. H., R. M. Andrews, M. L. Crump, A. H. Savitzky, K. D. Wells, and M. C. Brandley. 2016. Herpetology. Sinauer, Sunderland.

Purchon, R. D. 1977. The biology of the Mollusca. Pergamon, Oxford.

Rabosky, D. L., and E. E. Goldberg. 2015. Model inadequacy and mistaken inferences of trait-dependent speciation. Syst. Biol. 64:340–355.

Rainford, J. L., M. Hofreiter, D. B. D. Nicholson, and P. J. P. Mayhew. 2014. Phylogenetic distribution of extant richness suggests metamorphosis is a key innovation driving diversification in insects. PLoS One 9:e109085.

Ramel, G. 2012. The Phylum Chaetognatha. Available at: <http://www.earthlife.net/inverts/chaetognatha.html>.

Reiswig, H. M. 1971. Particle feeding in natural populations of three marine demosponges. Biol. Bull. 141:568–591

Ricci, C. 1984. Culturing of some bdelloid rotifers. Hydrobiologia 112:45–51.

Ricci, C., G. Melone, and E. J. Walsh. 2001. A carnivorous bdelloid rotifer*, Abrochtha carnivora n. sp*. Inv. Biol. 120:136–141.

Ripple, J. 1996. Sea turtles. Voyaguer Press, Minnesota.

Roskov Y., et al. 2019. Species 2000 and ITIS Catalogue of Life. Available at [www.catalogueoflife.org/col](http://www.catalogueoflife.org/col)

Rudman, W.B. 1999 Marine Pulmonate slugs. Available at: <http://www.seaslugforum.net/factsheet/onchid>

Rumpho, M. E., K. N. Pelletreau, A. Moustafa, and D. Bhattacharya. 2011. The making of a photosynthetic animal. J. Exp. Biol. 214:303–311.

Ruppert, E. E., R. D. Barnes, and R. S. Fox. 2004. Invertebrate zoology: a functional evolutionary approach. Brooks/Cole Publishing, Monterrey.

Rutman, J., and L. Fishelson. 1969. Food composition and feeding behavior of shallow-water crinoids at Eilat (Red Sea). Mar. Biol. 3:46–57.

Scholl, J. P., and J. J. Wiens. 2016. Diversification rates and species richness across the Tree of Life. Proc. R. Soc. Lond. B 283:20161334.

Segers, H. 2004. Rotifera: Monogononta. Freshwater invertebrates of the Malaysian region. Academy of Sciences Malaysia and Monash University, Kuala Lumpur.

Segers, H. 2007. Annotated checklist of the rotifers (phylum Rotifera), with notes on nomenclature, taxonomy and distribution. Zootaxa 1564:1–104.

Segers, H. 2007. Global diversity of rotifers (Rotifera) in freshwater. In: Freshwater Animal Diversity Assessment (E.V., Balian, C., Lévêque, H., Segers, K., Martens, eds.). Springer, Dordrecht.

Shapiro, L. 2012. Chaetognatha. Available at: <http://eol.org/pages/1740/overview>

South, A. 2012. Terrestrial slugs: Biology, ecology and control. Springer, New York.

Stephens, P. R., and J. J. Wiens. 2003. Ecological diversification and phylogeny of emydid turtles. Biol. J. Linn. Soc. 79:577–610.

Sterrer, W. 2001. Gnathostomulida (unsegmented marine worms). Available at: <http://www.els.net>

Strayer, D., W. Hummon, and R. Hochberg. 2010. Gastrotricha. In: Ecology and classification of North American freshwater invertebrates (J. Thorp and A. Covich, eds.). Academic Press, London.

Stork, N. E., J. McBroom, C. Gely, and A. J. Hamilton. 2015. New approaches narrow global species estimates for beetles, insects, and terrestrial arthropods. Proc. Natl. Acad. Sci. U.S.A. 112:7519–7523.

Strong, E. E., O. Gargominy, W. F. Ponder, and P. Bouchet. 2008. Global diversity of gastropods (Gastropoda; Mollusca) in freshwater. Hydrobiologia 595:149–166.

Sugiura, N. 1978. Further analysis of the data by Akaike's information criterion and the finite corrections. Commun. Stat. Theory Methods 7:13–26.

Summers, M. M., and G. W. Rouse. 2014. Phylogeny of Myzostomida (Annelida) and their relationships with echinoderm hosts. BMC Evol. Biol. 14:170.

Takano, T., and Y. Kano. 2014. Molecular phylogenetic investigations of the relationships of the echinoderm-parasite family Eulimidae within Hypsogastropoda (Mollusca). Mol. Phylogenet. Evol. 79:258–269.

Tatián, M., C. Lagger, M. Demarchi, and C. Mattoni. 2011. Molecular phylogeny endorses the relationship between carnivorous and filter‐feeding tunicates (Tunicata, Ascidiacea). Zool. Scr. 40:603–612.

Temereva, E. N., and V. V. Malakhov. 2010. Filter feeding mechanism in the phoronid *Phoronopsis harmeri* (Phoronida, Lophophorata). Russ. J. Mar. Biol. 36:109–116.

Temereva, E. N., and V. V. Malakhov. 2011. Organization of the epistome in *Phoronopsis harmeri* (Phoronida) and consideration of the coelomic organization in Phoronida. Zoomorphology 130:121–134.

Thompson, T.E. 1988. Molluscs: benthic Opisthobranchs (Mollusca: Gastropoda). Synopses of the British fauna 8:1–356.

Todt, C. 2013. Aplacophoran mollusks—Still obscure and difficult? Am. Malacol Bull. 31:181–187.

Trott, T. J. 1998. Gustatory responses of *Priapulus caudatus* de Lamarck, 1816 (Priapulida, Priapulidae): feeding behavior and chemoreception by a living fossil. Mar. Fresh. Behav. Physiol. 31:251–257.

Uetz, P., P. Freed, and J. Hosek. The Reptile Database. Available at: <http://www.reptile-database.org>

Vacelet, J., and E. Duport. 2004. Prey capture and digestion in the carnivorous sponge *Asbestopluma hypogea* (Porifera: Demospongiae). Zoomorphology 123:179–190.

Vacelet, J., and N. Boury-Esnault. 1996. A new species of carnivorous sponge (Demospongiae: Cladorhizidae) from a Mediterranean cave. Mém. Mus. R. His. Nat. Belg. 66:109–115

Vacelet, J., and N. Boury-Esnault. 1995. Carnivorous sponges. Nature 373:333–335

Valdés, A., and M. Adams. 2005. A new species of *Glossodoris* (Mollusca: Nudibranchia), of the *Glossodoris atromarginata* color group, from Indonesia. Pac. Sci. 59:603–608.

Vaughan, B. 2008. The Bivalve, *Poromya granulata*. Archerd Shell Collection. Available at: [http://shells.tricity.wsu.edu/ArcherdShellCollection/Illustrations/Poromya-Prey-Capture.html](http://shells.tricity.wsu.edu/ArcherdShellCollection/Illustrations/Poromya_Prey_Capture.html)

Vitt, L. J., and J. P. Caldwell. 2009. Herpetology. An introductory biology of amphibians and reptiles. Elsevier, Amsterdam.

Waggoner, B. 1999. Introduction to the Echinodermata. Available at <http://www.ucmp.berkeley.edu/echinodermata/echinodermata.html>.

Walker, J. C., and D. T. Anderson. 2001. The Platyhelminthes. In D. T. Anderson. Invertebrate Zoology. Oxford University Press, London.

Walter, D. E., and H. C. Proctor. 1998. Feeding behaviour and phylogeny: observations on early derivative Acari. Exp. Appl. Acarol. 22:39–50.

Walter D. E., and H. C. Proctor. 2013. Mites: Ecology, evolution, and behaviour: Life at a microscale. Springer, Dordrecht.

Webb, J. E. 1975. The distribution of Amphioxus. Symp. Zool. Soc. Lond. 36:179–212.

Weise, J. 1961. The ecology of *Urnatella gracilis* Leidy: Phylum Entoprocta. Limnol. Oceanogr. 6:228–230.

Wiens, J. J. 2015a. Faster diversification on land than sea helps explain global biodiversity patterns among habitats and animal phyla. Ecol. Lett. 18:1234–1241.

Wiens, J. J. 2015b. Explaining large-scale patterns of vertebrate diversity. Biol. Lett. 11:20150506.

Wilman, H., J. Belmaker, J. Simpson, C. de la Rosa, M. M. Rivadeneira, and W. Jetz. 2014. EltonTraits 1.0: Species‐level foraging attributes of the world's birds and mammals: Ecological Archives E095‐178. Ecology 95:2027–2027.

Yang, Z. 2006. Computational molecular evolution. Oxford University Press, London.

Yeates, G. W., T. D. Bongers, R. G. M. De Goede, D. W. Freckman, and S. S. Georgieva. 1993. Feeding habits in soil nematode families and genera—an outline for soil ecologists. J. Nematol. 25:315–331.

Zhang, Z. 2013. Animal biodiversity: an update of classification and diversity in 2013. Zootaxa 3703:5–11.

**Table S1.** Summary of diet and species richness for the 28 analyzed animal phyla. Starting left to right, we first show the frequency of herbivory among species in each phylum estimated from terminal taxa in the trees ("Herbivory (tree)"); the raw data are in Dataset S1. We also estimated the frequency of each diet (herbivory, carnivory, and omnivory) across all the species in each phylum (“Phylum-level Diet”), not just those in the tree, including two scenarios for Arthropoda and Chordata (“Scenarios 1 and 2”). Scenario 1 assumes the lowest estimate of herbivory in both phyla, whereas Scenario 2 assumes the highest frequency of herbivory. Within Arthropods, different scenarios are based on diet estimates in Grimaldi and Engel (2005) and Wiens et al. (2015). Within chordates, different scenarios are based on diet estimates summarized from Fishbase (Froese and Pauly 2019) and Bornstein et al. (2019). We also indicate the estimated frequency of herbivory (“Est-herbivory” column) in each phylum, which is the frequency of herbivorous species in each phylum, with omnivorous species treated as 50% herbivorous. We also present the number of taxa sampled in the tree for each phylum ("Taxa sampled”). The total number of described species in each phylum (“Total richness”) is derived from Zhang (2013). Finally, we show two estimates of the projected richness (“Projected-low” and “Projected-high” columns) based on different estimates in Larsen et al. (2017) for Arthropoda and Nematoda, as described above in the Supplementary Methods. Estimates for other phyla are based on Chapman (2009) and Appeltans et al. (2012). For some taxa Appeltans et al. (2012) presented a range of values for projected richness. We used the midpoint of these ranges. Finally, we present estimates of the frequency of herbivory for Arthropoda based on the projected richness of different groups within the phylum (see Supplementary Methods for details). The bottom row summarizes the frequency of each diet under the tree-based sampling and phylum-based estimates under two scenarios, in addition to the total number of species sampled in the tree, the total number of described animal species, and the total number of projected species under two scenarios.

| Phylum | Herbivory (tree) | Phylum-level Diet: Scenario 1 (Low herbivory) | | | | Phylum-level Diet: Scenario 2 (High herbivory) | | | | Richness | | | |  |
| --- | --- | --- | --- | --- | --- | --- | --- | --- | --- | --- | --- | --- | --- | --- |
|  |  | Herbivory | Carnivory | Omnivory | Est-Herbivory | Herbivory | Carnivory | Omnivory | Est-Herbivory | Taxa sampled | Total richness | Projected low  (herbivory) | Projected high  (herbivory) | Projected richness source |
| Acoela | 1.0000 | 0.0000 | 0.0000 | 1.0000 | 0.5000 | 0.0000 | 0.0000 | 1.0000 | 0.5000 | 1 | 421 | 4,100 | 4,100 | Appeltans et al. (2012) |
| Annelida | 0.6000 | 0.1940 | 0.5300 | 0.2740 | 0.3310 | 0.1940 | 0.5300 | 0.2740 | 0.3310 | 5 | 18,114 | 30,000 | 30,000 | Chapman (2009); Appeltans et al. (2012); 26,000–37,000 |
| Arthropoda | 0.3400 | 0.3570 | 0.6420 | 0.0000 | 0.3570 | 0.4010 | 0.5980 | 0.0000 | 0.4010 | 921 | 1,257,040 | 13,600,000  (0.191) | 97,800,000  (0.325) | see above (Larsen et al. 2017) |
| Brachiopoda | 1.0000 | 1.0000 | 0.0000 | 0.0000 | 1.0000 | 1.0000 | 0.0000 | 0.0000 | 1.0000 | 1 | 394 | 394 | 394 | Appeltans et al. (2012) estimated only 388 |
| Bryozoa | 0.5000 | 1.0000 | 0.0000 | 0.0000 | 1.0000 | 1.0000 | 0.0000 | 0.0000 | 1.0000 | 2 | 6,029 | 9,900 | 9,900 | Appeltans et al. (2012): 8,700–11,100 |
| Chaetognatha | 0.0000 | 0.0000 | 1.0000 | 0.0000 | 0.0000 | 0.0000 | 1.0000 | 0.0000 | 0.0000 | 2 | 131 | 331 | 331 | Appeltans et al. (2012): 179–483 |
| Chordata | 0.1600 | 0.198 | 0.727 | 0.007 | 0.2015 | 0.2070 | 0.5920 | 0.2000 | 0.3070 | 52 | 69,255 | 80,500 | 80,500 | Chapman (2009) |
| Cnidaria | 0.0000 | 0.0000 | 1.0000 | 0.0000 | 0.0000 | 0.0000 | 1.0000 | 0.0000 | 0.0000 | 7 | 13,323 | 15,663 | 15,663 | Summed midpoints across groups from Appeltans et al. (2012) |
| Ctenophora | 0.0000 | 0.0000 | 1.0000 | 0.0000 | 0.0000 | 0.0000 | 1.0000 | 0.0000 | 0.0000 | 1 | 187 | 408 | 408 | Appeltans et al. (2012): 315–500 |
| Echinodermata | 0.1000 | 0.0000 | 0.8540 | 0.1460 | 0.0730 | 0.0000 | 0.8540 | 0.1460 | 0.0730 | 5 | 7,288 | 14,000 | 14,000 | Chapman (2009) |
| Entoprocta | 1.0000 | 0.9950 | 0.0050 | 0.0000 | 0.9950 | 0.9950 | 0.0050 | 0.0000 | 0.9950 | 1 | 174 | 1,223 | 1,223 | Appeltans et al. (2012) |
| Gastrotricha | 1.0000 | 1.0000 | 0.0000 | 0.0000 | 1.0000 | 1.0000 | 0.0000 | 0.0000 | 1.0000 | 1 | 812 | 2,744 | 2,744 | Appeltans et al. (2012): 2244–3244 |
| Gnathostomulida | 0.0000 | 0.0000 | 1.0000 | 0.0000 | 0.0000 | 0.0000 | 1.0000 | 0.0000 | 0.0000 | 1 | 97 | 315 | 315 | Appeltans et al. (2012): 313–318 |
| Hemichordata | 0.7500 | 1.0000 | 0.0000 | 0.0000 | 1.0000 | 1.0000 | 0.0000 | 0.0000 | 1.0000 | 2 | 130 | 130 | 130 | Appeltans et al. (2012) estimated only 128 |
| Kinorhyncha | 1.0000 | 1.0000 | 0.0000 | 0.0000 | 1.0000 | 1.0000 | 0.0000 | 0.0000 | 1.0000 | 1 | 186 | 186 | 186 | Current richness |
| Mollusca | 0.6100 | 0.7159 | 0.2720 | 0.0120 | 0.7219 | 0.7159 | 0.2720 | 0.0120 | 0.7219 | 70 | 73,006 | 200,000 | 200,000 | Chapman (2009) |
| Nematoda | 0.0000 | 0.0220 | 0.9510 | 0.0270 | 0.0355 | 0.0220 | 0.9510 | 0.0270 | 0.0355 | 5 | 25,035 | 6,800,000 | 40,800,000 | see above (Larsen et al. 2017) |
| Nematomorpha | 0.0000 | 0.0000 | 1.0000 | 0.0000 | 0.0000 | 0.0000 | 1.0000 | 0.0000 | 0.0000 | 1 | 339 | 339 | 339 | Current richness |
| Nemertea | 0.0000 | 0.0010 | 0.9990 | 0.0000 | 0.0010 | 0.0010 | 0.9990 | 0.0000 | 0.0010 | 1 | 1,358 | 19853 | 19,853 | Appeltans et al. (2012): 1985–2685 |
| Onychophora | 0.0000 | 0.0000 | 1.0000 | 0.0000 | 0.0000 | 0.0000 | 1.0000 | 0.0000 | 0.0000 | 1 | 183 | 220 | 220 | Chapman (2009) |
| Phoronida | 1.0000 | 0.0000 | 0.0000 | 1.0000 | 0.5000 | 0.0000 | 0.0000 | 1.0000 | 0.5000 | 1 | 16 | 18 | 18 | Appeltans et al. (2012) |
| Placozoa | 0.5000 | 0.0000 | 0.0000 | 1.0000 | 0.5000 | 0.0000 | 0.0000 | 1.0000 | 0.5000 | 1 | 1 | 74 | 74 | Appeltans et al. (2012): 29-119 |
| Platyhelminthes | 0.0000 | 0.0010 | 0.9990 | 0.0000 | 0.0010 | 0.0010 | 0.9990 | 0.0000 | 0.0010 | 1 | 29,275 | 80,000 | 80,000 | Chapman (2009) |
| Porifera | 0.5000 | 0.0000 | 0.0200 | 0.9790 | 0.4895 | 0.0000 | 0.0200 | 0.9790 | 0.4895 | 2 | 8,514 | 26,203 | 26,203 | Appeltans et al. (2012): 25,853–26,553 |
| Priapulida | 0.5000 | 0.0000 | 0.0000 | 1.0000 | 0.5000 | 0.0000 | 0.0000 | 1.0000 | 0.5000 | 1 | 19 | 19 | 19 | Current richness |
| Rotifera | 1.0000 | 0.4984 | 0.5015 | 0.0000 | 0.5015 | 0.4984 | 0.5015 | 0.0000 | 0.5015 | 1 | 3,246 | 3,246 | 3,246 | Current richness: both Chapman (2009) and Appeltans et al. (2012) had estimates lower then current richness |
| Tardigrada | 1.0000 | 0.9990 | 0.0010 | 0.0000 | 0.9990 | 0.9990 | 0.0010 | 0.0000 | 0.9990 | 2 | 1,167 | 1,303 | 1,303 | Appeltans et al. (2012) |
| Xenoturbellida | 0.0000 | 0.0000 | 1.0000 | 0.0000 | 0.0000 | 0.0000 | 1.0000 | 0.0000 | 0.0000 | 1 | 2 | 2 | 2 | Current richness |
|  |  |  |  |  |  |  |  |  |  |  |  |  |  |  |
| Summary | 0.3354 | 0.3495 | 0.6355 | 0.0111 | 0.3550 | 0.3864 | 0.5928 | 0.0199 | 0.3964 | 1087 | 1,515,954 | 20,891,171 | 139,091,171 |  |

**Table S2.** Comparison of the fit of different models for the evolution of diet, and estimated level of phylogenetic signal (lambda) for Tree II. The relative fit of two models was compared based on AICc values: a model with no phylogenetic signal (white noise model) and one with phylogenetic signal (lambda model). The best-fitting model is boldfaced. The estimated value of lambda quantifies the level of phylogenetic signal, from 0 to 1 (maximum signal). Significant lambda values (*P*<0.0001; tested by simulations) are asterisked. For these analyses, we initially included all three states (i.e. carnivorous, herbivorous, and omnivorous; the 2% of taxa with ambiguous or unknown states were excluded). Alternatively, all omnivorous and ambiguous taxa (5% of all sampled taxa) were coded as either carnivorous (maxcar) or herbivorous (maxherb).

| Dataset | Model | Ln-likelihood | AICc |
| --- | --- | --- | --- |
| Three states | White-noise | -1059.794 | 2123.588 |
|  | Lambda (λ=0.81*) | -835.582 | **1677.705** |
| Maxcar | White-noise | -713.628 | 1431.267 |
|  | Lambda (λ=0.86*) | -298.268 | **602.558** |
| Maxherb | White-noise | -750.787 | 1505.586 |
|  | Lambda (λ=0.87*) | -346.178 | **698.378** |

**Table S3.** Testing for phylogenetic signal in diet using the D-statistic and Tree II. Estimated D is scaled based on D-values simulated under the Brownian motion model (strong phylogenetic signal) and random noise (no phylogenetic signal). Smaller values indicate stronger support for phylogenetic signal, with negative values showing that traits are highly conserved. Probabilities (*P*-values) indicate whether the observed D-statistic is significantly different from 0 (Brownian motion) and from 1 (random noise). Since the D-statistic is designed for binary data, two coding strategies were used, treating omnivorous and ambiguous taxa (5% of all sampled taxa) as either carnivorous (maxcar) or herbivorous (maxherb).

| Coding strategy | Estimated D | Probability of D different from Brownian motion (strong signal) | Probability of D different from random noise (no signal) |
| --- | --- | --- | --- |
| Maxcar | -0.468 | 0.997 | <0.0001 |
| Maxherb | -0.437 | 0.998 | <0.0001 |

**Table S4.** Comparison of the fit of different models for the evolution of diet, and estimated levels of phylogenetic signal (lambda) for Tree III. The relative fit of two models was compared based on AICc values: a model with no phylogenetic signal (white noise model) and one with phylogenetic signal (lambda model). The best-fitting model is boldfaced. The estimated value of lambda quantifies the level of phylogenetic signal, from 0 to 1 (maximum signal). Significant lambda values (*P*<0.0001; tested by simulations) are asterisked. For these analyses, we initially included all three states (i.e. carnivorous, herbivorous, and omnivorous; the 2% of taxa with ambiguous or unknown states were excluded). Alternatively, all omnivorous and ambiguous taxa (5% of all sampled taxa) were coded as either carnivorous (maxcar) or herbivorous (maxherb).

| Dataset | Model | Ln-likelihood | AICc |
| --- | --- | --- | --- |
| Three states | White-noise | -1069.580 | 2143.1610 |
|  | Lambda (λ=0.83*) | -843.483 | **1692.966** |
|  |  |  |  |
| Maxcar | White-noise | -715.270 | 1434.551 |
|  | Lambda (λ=0.87*) | -301.203 | **608.406** |
|  |  |  |  |
| Maxherb | White-noise | -750.787 | 1505.586 |
|  | Lambda (λ=0.87*) | -345.736 | **697.495** |

**Table S5.** Testing for phylogenetic signal in diet using the D-statistic and Tree III. Estimated D is scaled based on D-values simulated under the Brownian motion model (strong phylogenetic signal) and random noise (no phylogenetic signal). Smaller values indicate stronger support for phylogenetic signal, with negative values showing that traits are highly conserved. Probabilities (*P*-values) indicate whether the observed D-statistic is significantly different from 0 (Brownian motion) and from 1 (random noise). Since the D-statistic is designed for binary data, two coding strategies were used, treating omnivorous and ambiguous taxa (5% of all sampled taxa) as either carnivorous (maxcar) or herbivorous (maxherb).

| Coding strategy | Estimated D | Probability of D different from Brownian motion (strong signal) | Probability of D different from random noise (no signal) |
| --- | --- | --- | --- |
| Maxcar | -0.479 | 0.994 | <0.0001 |
| Maxherb | -0.440 | 0.994 | <0.0001 |

**Table S6.** Comparison of the fit of different likelihood models for the evolution of animal diet (using HiSSE) for Tree I. Given that HiSSE allows only two states per character, omnivorous species (or taxa with ambiguous states) were either coded as carnivorous (state 1; maxcar) in one set of analyses, or as herbivorous in the other (state 0; maxherb). For each coding approach, we fitted a total of 38 HiSSE models, including two standard null models and 36 alternative models of trait evolution and state-dependent diversification. Observed states were coded as 0 (herbivorous) or 1 (carnivorous), and the hidden states as A or B. The default (maddfitz) option was used for estimating the initial probabilities of each state at the root (the default option in HiSSE). Log-likelihood (i.e., natural logarithm; lnL) and ΔAICc values are shown for each model. The states included in each model are also summarized. We also describe the transition matrix structure (i.e. allowed vs. constrained transitions) and whether transitions rates differed or not between states. Constraints on speciation rates (λ) and extinction rates (μ) are also summarized for each model (i.e. whether two states are constrained to have equal rates or whether their rates are allowed to differ). Values for the best-fitting models are boldfaced. The models are arranged as follows. There are six sets of six models each (plus two null models). These six sets of models were designed to systematically explore the different combinations of: (1) possible transitions and transition rates in the transition matrix, (2) different numbers of hidden states per observed state, and (3) constraints on speciation and extinction rates. Model set 1 allowed all transitions but excluded state 0B (i.e. no hidden state in observed state 0). Model set 2 was equivalent to model set 1, but excluded state 1B (no hidden state in observed state 1). Model set 3 included all states but disallowed transitions between states 0B and 1B. Model set 4 included all states but disallowed 1A-0B and 1B-0A state transitions. Model set 5 included all states and allowed all transitions. Lastly, model set 6 included all the states but disallowed 0A-1A transitions. Within each set of six models, the first model (i.e. M1, M7, M13, M19, M25, M31) assumed equal transition rates between states and a single rate for speciation and a single rate for extinction across all observed and hidden states. The second model (M2, etc.) constrained transition rates between states to be equal but allowed speciation and extinction rates to vary between states. The third model (M3, etc.) assumed equal transition rates between states and allowed different speciation and extinction rates for each state. The fourth model (M4, etc.) allowed different transition rates between states but had only a single parameter for speciation and another for extinction across all states. The fifth model (M5, etc.) allowed different transition rates between all states and different speciation and extinction rates for each state. The sixth model (M6, etc.) assumed all transition rates were different, but speciation and extinction rates were each constrained to be equal between the hidden states of the different observed states (i.e. 0A=1A, 0B=1B), such that rates differed between the hidden states and not the observed states (as in the null models). The two standard null models have effectively same number of variable parameters as a BiSSE-like model (i.e. “Null-two”) or HiSSE-like model (i.e. “Null-four”). However, in the “null-two” model there are different speciation and different extinction rates for each of the hidden states (A or B) but speciation and extinction rates are constrained to be equal between the observed states (0, 1). “Null-four” includes a total of four hidden states (A, B, C, D) within each observed state (0, 1). Each hidden state had different speciation rates and different extinction rates, but speciation and extinction rates were constrained to be equal between the observed states. Thus, for the two null models, there is no effect of the observed state on diversification. The sixth set of models is similar to the null models, but in the “standard” null models the transition rates are constrained to be equal between states, whereas in the sixth set of models transition rates were allowed to be different.

| Model set | Model name | Included states | Transition matrix | | λ | μ | Maxcar | | Maxherb | |
| --- | --- | --- | --- | --- | --- | --- | --- | --- | --- | --- |
|  |  |  | Structure* | Rates |  |  | lnL | ΔΑΙCc | lnL | ΔΑΙCc |
| Set 1 | M1 | 0A,1A,1B | All transitions included | All equal | 0A=1A=1B | 0A=1A=1B | -6977.78 | 396.69 | -7162.06 | 713.36 |
|  | M2 | 0A,1A,1B | All transitions included | All equal | 0A≠1A≠1B | 0A≠1A≠1B | -7119.74 | 668.47 | -7081.66 | 560.66 |
|  | M3 | 0A,1A,1B | All transitions included | All equal | 0A; 1A; 1B | 0A=1A; 1B | -7035.35 | 507.78 | -7099.20 | 591.68 |
|  | M4 | 0A,1A,1B | All transitions included | Different | 0A=1A=1B | 0A=1A=1B | -7043.16 | 519.35 | -7109.44 | 614.19 |
|  | M5 | 0A,1A,1B | All transitions included | Different | 0A≠1A≠1B | 0A≠1A≠1B | -7084.36 | 603.77 | -6901.95 | 207.34 |
|  | M6 | 0A,1A,1B | All transitions included | Different | 0A=1A; 1B | 0A=1A; 1B | -6857.40 | 157.97 | -6986.76 | 372.89 |
| Set 2 | M7 | 0A,0B,1A | All transitions included | All equal | 0A=1A=0B | 0A=1A=0B | -6892.80 | 224.70 | -7092.26 | 573.77 |
|  | M8 | 0A,0B,1A | All transitions included | All equal | 0A≠1A≠0B | 0A≠1A≠0B | -7115.76 | 660.50 | -7124.86 | 647.06 |
|  | M9 | 0A,0B,1A | All transitions included | All equal | 0A=1A; 0B | 0A=1A; 0B | -7065.23 | 567.53 | -7130.68 | 654.64 |
|  | M10 | 0A,0B,1A | All transitions included | Different | 0A=1A=0B | 0A=1A=0B | -7065.66 | 564.35 | -7115.57 | 626.44 |
|  | M11 | 0A,0B,1A | All transitions included | Different | 0A≠1A≠0B | 0A≠1A≠0B | -7081.18 | 597.40 | -6950.41 | 304.25 |
|  | M12 | 0A,0B,1A | All transitions included | Different | 0A=1A; 0B | 0A=1A; 0B | -6997.90 | 438.97 | -7102.51 | 604.38 |
| Set 3 | M13 | 0A,0B,1A,1B | 0B-1B not allowed | All equal | 0A=1A=0B=1B | 0A=1A=0B=1B | -7004.07 | 447.25 | -7130.38 | 650.00 |
|  | M14 | 0A,0B,1A,1B | 0B-1B not allowed | All equal | 0A≠1A≠0B≠1B | 0A≠1A≠0B≠1B | -7125.40 | 679.80 | -7009.89 | 419.12 |
|  | M15 | 0A,0B,1A,1B | 0B-1B not allowed | All equal | 0A=1A; 0B=1B | 0A=1A; 0B=1B | -6977.50 | 394.10 | -7039.93 | 473.13 |
|  | M16 | 0A,0B,1A,1B | 0B-1B not allowed | Different | 0A=1A=0B=1B | 0A=1A=0B=1B | -7046.63 | 526.29 | -7094.66 | 588.67 |
|  | M17 | 0A,0B,1A,1B | 0B-1B not allowed | Different | 0A≠1A≠0B≠1B | 0A≠1A≠0B≠1B | -7066.40 | 571.90 | -6839.94 | 91.48 |
|  | M18 | 0A,0B,1A,1B | 0B-1B not allowed | Different | 0A=1A; 0B=1B | 0A=1A; 0B=1B | -6796.72 | 44.79 | -7072.71 | 544.77 |
| Set 4 | M19 | 0A,0B,1A,1B | 1A-0B, 1B-0A not allowed | All equal | 0A=1A=0B=1B | 0A=1A=0B=1B | -7004.07 | 447.25 | -7168.64 | 726.53 |
|  | M20 | 0A,0B,1A,1B | 1A-0B, 1B-0A not allowed | All equal | 0A≠1A≠0B≠1B | 0A≠1A≠0B≠1B | -7126.43 | 681.84 | -7064.01 | 527.37 |
|  | M21 | 0A,0B,1A,1B | 1A-0B, 1B-0A not allowed | All equal | 0A=1A; 0B=1B | 0A=1A; 0B=1B | -7029.13 | 497.36 | -7054.69 | 502.65 |
|  | M22 | 0A,0B,1A,1B | 1A-0B, 1B-0A not allowed | Different | 0A=1A=0B=1B | 0A=1A=0B=1B | -7042.74 | 518.51 | -7080.51 | 564.44 |
|  | M23 | 0A,0B,1A,1B | 1A-0B, 1B-0A not allowed | Different | 0A≠1A≠0B≠1B | 0A≠1A≠0B≠1B | -7084.36 | 611.90 | -6822.50 | 6.72 |
|  | **M24** | **0A,0B,1A,1B** | **1A-0B, 1B-0A not allowed** | **Different** | **0A=1A; 0B=1B** | **0A=1A; 0B=1B** | **-6772.26** | **0.00** | **-6796.25** | **0.00** |
| Set 5 | M25 | 0A,0B,1A,1B | All transitions included | All equal | 0A=1A=0B=1B | 0A=1A=0B=1B | -6841.84 | 130.94 | -7154.22 | 697.68 |
|  | M26 | 0A,0B,1A,1B | All transitions included | All equal | 0A≠1A≠0B≠1B | 0A≠1A≠0B≠1B | -7126.43 | 681.84 | -7048.46 | 496.27 |
|  | M27 | 0A,0B,1A,1B | All transitions included | All equal | 0A=1A; 0B=1B | 0A=1A; 0B=1B | -7034.9 | 508.90 | -7088.74 | 570.76 |
|  | M28 | 0A,0B,1A,1B | All transitions included | Different | 0A=1A=0B=1B | 0A=1A=0B=1B | -7058.59 | 550.21 | -7154.94 | 721.48 |
|  | M29 | 0A,0B,1A,1B | All transitions included | Different | 0A≠1A≠0B≠1B | 0A≠1A≠0B≠1B | -7084.36 | 620.07 | -6799.38 | 22.76 |
|  | M30 | 0A,0B,1A,1B | All transitions included | Different | 0A=1A; 0B=1B | 0A=1A; 0B=1B | -6774.79 | 13.33 | -6899.97 | 215.67 |
| Set 6 | M31 | 0A,0B,1A,1B | 0A-1A not allowed | All equal | 0A=1A=0B=1B | 0A=1A=0B=1B | -7125.40 | 679.79 | -7130.38 | 650.00 |
|  | M32 | 0A,0B,1A,1B | 0A-1A not allowed | All equal | 0A≠1A≠0B≠1B | 0A≠1A≠0B≠1B | -6977.49 | 394.09 | -7024.45 | 448.26 |
|  | M33 | 0A,0B,1A,1B | 0A-1A not allowed | All equal | 0A=1A; 0B=1B | 0A=1A; 0B=1B | -7046.63 | 526.29 | -7039.92 | 473.13 |
|  | M34 | 0A,0B,1A,1B | 0A-1A not allowed | Different | 0A=1A=0B=1B | 0A=1A=0B=1B | -7066.39 | 571.88 | -7081.45 | 562.26 |
|  | M35 | 0A,0B,1A,1B | 0A-1A not allowed | Different | 0A≠1A≠0B≠1B | 0A≠1A≠0B≠1B | -6796.71 | 44.79 | -6824.98 | 61.57 |
|  | M36 | 0A,0B,1A,1B | 0A-1A not allowed | Different | 0A=1A; 0B=1B | 0A=1A; 0B=1B | -6810.64 | 64.45 | -6842.03 | 87.50 |
| Classical null | Null-two | 0A,0B,1A,1B | 1B-0A, 0B-1A not allowed | All equal | 0A=1A; 0B=1B | 0A=1A; 0B=1B | -7121.95 | 674.87 | -7054.68 | 502.65 |
|  | Null-four | 0A,0B,1A,1B | All transitions included | All equal | 1A=0A; 0B=1B; 0C=1C; 0D=1D | 1A=0A; 0B=1B; 0C=1C; 0D=1D | -6977.78 | 396.69 | -6974.69 | 350.76 |

*****Dual transitions are considered for the fitted models.

**Table S7.** Rates of speciation (λ) and extinction (μ) estimated from the best-fitting HiSSE model (M24) for each coding strategy (maxcar and maxherb) using Tree I. Model parameters are described in Table S6. Observed states were coded as either 0 (herbivorous) or 1 (carnivorous), and the hidden states are A and B. These results show little support for different diversification rates between the observed diet states.

| State | Maxcar | | Maxherb | |
| --- | --- | --- | --- | --- |
|  | λ | μ | λ | μ |
| 0A | 1.61E-02 | 4.02E-19 | 2.73E-3 | 3.47E-04 |
| 1A | 1.61E-02 | 4.02E-19 | 2.73E-3 | 3.47E-04 |
| 0B | 7.52E-53 | 5.02E-51 | 1.99E-2 | 9.53E-04 |
| 1B | 7.52E-53 | 5.02E-51 | 1.99E-2 | 9.53E-04 |

**Table S8.** Comparison of the fit of different likelihood models for the evolution of animal diet (using HiSSE) for Tree II. Given that HiSSE allows only two states per character, omnivorous species (or taxa with ambiguous states) were either coded as carnivorous (state 1; maxcar) in one set of analyses, or as herbivorous in the other (state 0; maxherb). For each coding approach, we fit a total of five SSE models, including a BiSSE model, two standard null HiSSE models, an alternative HiSSE null model (i.e. M24; see Table S6), and a full HiSSE model. These models were used for analyzing trait evolution and state-dependent diversification across the tree. Observed states were coded as 0 (herbivorous) or 1 (carnivorous), and the hidden states as A or B. The default (maddfitz) option was used for estimating the initial probabilities of each state at the root. Log-likelihood (i.e., natural logarithm; lnL) and AICc values are shown for each model. We fitted a full BiSSE model, with independent diversification parameters between the two observed states (i.e. 0, 1). The two standard null models have effectively same number of variable parameters as a BiSSE-like model (i.e. “Null-two”) or HiSSE-like model (i.e. “Null-four”). However, in the “null-two” model there are different speciation and different extinction rates for each of the hidden states (A or B) but speciation and extinction rates are constrained to be equal between the observed states (0, 1). “Null-four” includes a total of four hidden states (A, B, C, D) within each observed state (0, 1). Each hidden state had different speciation rates and different extinction rates, but speciation and extinction rates were constrained to be equal between the observed states. Thus, for the two null models, there is no effect of the observed state on diversification. Next, M24 included all states but disallowed 1A-0B and 1B-0A state transitions. This model is also similar to the null models, except that in the “standard” null models the transition rates are constrained to be equal between states, whereas in the M24 models transition rates were allowed to be different. Finally, the full HiSSE includes independent diversification parameters for each hidden state, and allows all transitions between states. ΔΑΙCc relative to the best-fitting model (M24 in this case).

| Model | Included states | Transition matrix | | λ | μ | Maxcar | | Maxherb | |
| --- | --- | --- | --- | --- | --- | --- | --- | --- | --- |
|  |  | Structure* | Rates |  |  | lnL | ΔΑΙCc | lnL | ΔΑΙCc |
| BiSSE | 0,1 | All transitions included | Different | 0≠1 | 0≠1 | -7159.67 | 751.43 | -7165.16 | 689.53 |
| Null-two | 0A,0B,1A,1B | 1B-0A, 0B-1A not allowed | All equal | 0A=1A; 0B=1B | 0A=1A; 0B=1B | -7071.14 | 576.42 | -7062.64 | 486.54 |
| Null-four | 0A,0B,1A,1B | All transitions included | All equal | 1A=0A; 0B=1B; 0C=1C; 0D=1D | 1A=0A; 0B=1B; 0C=1C; 0D=1D | -6956.84 | 355.93 | -6979.50 | 328.37 |
| M24 | 0A,0B,1A,1B | 1A-0B, 1B-0A not allowed | Different | 0A=1A; 0B=1B | 0A=1A; 0B=1B | -6775.95 | 0 | -6812.25 | 0 |
| Full HiSSE | 0A,0B,1A,1B | All transitions included | Different | 0A≠1A≠0B≠1B | 0A≠1A≠0B≠1B | -7139.26 | 708.63 | -7168.26 | 693.75 |

*****Dual transitions are considered for the fitted models.

**Table S9.** Comparison of the fit of different likelihood models for the evolution of animal diet (using HiSSE) for Tree III. Given that HiSSE allows only two states per character, omnivorous species (or taxa with ambiguous states) were either coded as carnivorous (state 1; maxcar) in one set of analyses, or as herbivorous in the other (state 0; maxherb). For each coding approach, we fitted a total of five SSE models, including a BiSSE model, two standard null HiSSE models, an alternative HiSSE null model (i.e. M24; see Table S6), and a full HiSSE. These models were used for analyzing trait evolution and state-dependent diversification across the tree. Observed states were coded as 0 (herbivorous) or 1 (carnivorous), and the hidden states as A or B. The default (maddfitz) option was used for estimating the initial probabilities of each state at the root. Log-likelihood (i.e., natural logarithm; lnL) and AICc values are shown for each model. We fitted a full BiSSE model, with independent diversification parameters between the two observed states (i.e. 0, 1). The two standard null models have effectively same number of variable parameters as a BiSSE-like model (i.e. “Null-two”) or HiSSE-like model (i.e. “Null-four”). However, in the “null-two” model there are different speciation and different extinction rates for each of the hidden states (A or B) but speciation and extinction rates are constrained to be equal between the observed states (0, 1). “Null-four” includes a total of four hidden states (A, B, C, D) within each observed state (0, 1). Each hidden state had different speciation rates and different extinction rates, but speciation and extinction rates were constrained to be equal between the observed states. Thus, for the two null models, there is no effect of the observed state on diversification. Next, M24 included all states but disallowed 1A-0B and 1B-0A state transitions. This model is also similar to the null models, except that in the “standard” null models the transition rates are constrained to be equal between states, whereas in the M24 models transition rates were allowed to be different. Finally, the full HiSSE includes independent diversification parameters for each hidden state, and allows all transitions between states. ΔΑΙCc relative to the best-fitting model (M24 in this case).

| Model | Included states | Transition matrix | | λ | μ | Maxcar | | Maxherb | |
| --- | --- | --- | --- | --- | --- | --- | --- | --- | --- |
|  |  | Structure* | Rates |  |  | lnL | ΔΑΙCc | lnL | ΔΑΙCc |
| BiSSE | 0,1 | All transitions included | Different | 0≠1 | 0≠1 | -7159.67 | 642.97 | -7159.49 | 690.06 |
| Null-two | 0A,0B,1A,1B | 1B-0A, 0B-1A not allowed | All equal | 0A=1A; 0B=1B | 0A=1A; 0B=1B | -7071.14 | 467.96 | -7030.12 | 433.38 |
| Null-four | 0A,0B,1A,1B | All transitions included | All equal | 1A=0A; 0B=1B; 0C=1C; 0D=1D | 1A=0A; 0B=1B; 0C=1C; 0D=1D | -6956.84 | 247.47 | -6976.24 | 333.73 |
| M24 | 0A,0B,1A,1B | 1A-0B, 1B-0A not allowed | Different | 0A=1A; 0B=1B | 0A=1A; 0B=1B | -6773.97 | 0 | -6806.32 | 0 |
| Full HiSSE | 0A,0B,1A,1B | All transitions included | Different | 0A≠1A≠0B≠1B | 0A≠1A≠0B≠1B | -7139.26 | 600.17 | -7074.51 | 528.23 |

*****Dual transitions are considered for the fitted models.

**Table S10.** Estimated proportions of herbivorous, non-marine, and terrestrial species and estimated net diversification rates for each of 28 animal phyla (using three different relative extinction fractions, ε) based on Tree I. Proportions of herbivorous species were estimated from the diet data for each phylum in Dataset S1. Rate estimates and data on habitat (non-marine [terrestrial and freshwater] vs. marine, and terrestrial vs. aquatic [marine and freshwater]) are from Wiens (2015a).

| Phylum | Herbivorous | Non-marine | Terrestrial | ε = 0 | ε = 0.5 | ε = 0.9 |
| --- | --- | --- | --- | --- | --- | --- |
| Acoela | 1.0000 | 0 | 0 | 0.0091709 | 0.0081225 | 0.0057084 |
| Annelida | 0.6000 | 0.28 | 0.19 | 0.0167623 | 0.0155774 | 0.0128265 |
| Arthropoda | 0.3400 | 0.96 | 0.86 | 0.0244300 | 0.0232242 | 0.0204246 |
| Brachiopoda | 1.0000 | 0 | 0 | 0.0113725 | 0.0100583 | 0.0070338 |
| Bryozoa | 0.5000 | 0.02 | 0 | 0.0149539 | 0.0137634 | 0.0110007 |
| Chaetognatha | 0.0000 | 0 | 0 | 0.0071632 | 0.0061559 | 0.0038776 |
| Chordata | 0.1600 | 0.73 | 0.38 | 0.0171470 | 0.0160807 | 0.0136048 |
| Cnidaria | 0.0000 | 0.15 | 0 | 0.0120706 | 0.0111897 | 0.0091449 |
| Ctenophora | 0.0000 | 0 | 0 | 0.0059717 | 0.0051865 | 0.0033968 |
| Echinodermata | 0.1000 | 0 | 0 | 0.0163751 | 0.0150992 | 0.0121380 |
| Entoprocta | 1.0000 | 0.01 | 0 | 0.0088632 | 0.0076822 | 0.0049940 |
| Gastrotricha | 1.0000 | 0.40 | 0 | 0.0132172 | 0.0118522 | 0.0086963 |
| Gnathostomulida | 0.0000 | 0 | 0 | 0.0100998 | 0.0085922 | 0.0052122 |
| Hemichordata | 0.7500 | 0 | 0 | 0.0089618 | 0.0076998 | 0.0048457 |
| Kinorhyncha | 1.0000 | 0 | 0 | 0.0100483 | 0.0087258 | 0.0057117 |
| Mollusca | 0.6100 | 0.40 | 0.33 | 0.0186133 | 0.0174612 | 0.0147862 |
| Nematoda | 0.0000 | 0.72 | 0.65 | 0.0254797 | 0.0237360 | 0.0196879 |
| Nematomorpha | 0.0000 | 0.98 | 0 | 0.0146568 | 0.0129205 | 0.0089300 |
| Nemertea | 0.0000 | 0.03 | 0.01 | 0.0129914 | 0.0117444 | 0.0088565 |
| Onychophora | 0.0000 | 1.00 | 1.00 | 0.0090619 | 0.0078656 | 0.0051401 |
| Phoronida | 1.0000 | 0 | 0 | 0.0052760 | 0.0040724 | 0.0017436 |
| Placozoa | 0.0000 | 0 | 0 | 0.0000000 | 0.0000000 | 0.0000000 |
| Platyhelminthes | 0.0000 | 0.60 | 0.30 | 0.0203042 | 0.0189367 | 0.0157621 |
| Porifera | 0.5000 | 0.03 | 0 | 0.0108183 | 0.0099898 | 0.0080669 |
| Priapulida | 0.5000 | 0 | 0 | 0.0056617 | 0.0044275 | 0.0019798 |
| Rotifera | 1.0000 | 0.82 | 0.42 | 0.0178501 | 0.0163205 | 0.0127727 |
| Tardigrada | 1.0000 | 0.85 | 0.80 | 0.0121065 | 0.0109197 | 0.0081724 |
| Xenoturbellida | 0.0000 | 0 | 0 | 0.0010520 | 0.0006154 | 0.0001447 |

**Table S11.** Results of PGLS analyses of the relationship between diet (proportion of herbivorous species) and net diversification rates among 28 animal phyla based on Tree I. Net diversification rates were analyzed under three relative extinction fractions (ε = 0 [low]; 0.5 [medium], and 0.9 [high]; Table S10). We present results for three different estimates of the frequency of herbivory within each of the analyzed phylum. First, an estimate based on Dataset S1, using the species included in the tree (“Tree”). Second, based on estimates across all species for each phylum (see Supplementary Methods), including estimates assuming a low frequency of herbivory (Scenario 1) and a high frequency of herbivory (Scenario 2). Details on each of these herbivory scenarios are provided in Table S1. We also evaluated models accounting for the effect of habitat (non-marine [terrestrial and freshwater] vs. marine, and terrestrial vs. aquatic [marine and freshwater]), and its interaction with diet. Data for each phylum are given in Tables S1 and S10. Significant PGLS models are highlighted in gray.

| Epsilon | Predictor(s) | Slope | *r*^2^ | *P* |
| --- | --- | --- | --- | --- |
| low | Herbivory (Tree) | -1.00E-03 | 0.005 | 0.716 |
| low | Herbivory (Scenario 1) | -1.63E-03 | 0.012 | 0.575 |
| low | Herbivory (Scenario 2) | -1.39E-03 | 0.009 | 0.634 |
| low | Non-marine | 9.91E-05 | 0.369 | 0.001 |
| low | Terrestrial | 1.04E-04 | 0.262 | 0.005 |
| med | Herbivory (Tree) | -6.14E-04 | 0.002 | 0.822 |
| med | Herbivory (Scenario 1) | -1.25E-03 | 0.007 | 0.668 |
| med | Herbivory (Scenario 2) | -1.01E-03 | 0.005 | 0.729 |
| med | Non-marine | 9.52E-05 | 0.366 | 0.001 |
| med | Terrestrial | 1.01E-04 | 0.267 | 0.005 |
| high | Herbivory (Tree) | -1.01E-03 | 0.006 | 0.685 |
| high | Herbivory (Scenario 1) | -1.38E-03 | 0.011 | 0.602 |
| high | Herbivory (Scenario 2) | -1.14E-03 | 0.007 | 0.667 |
| high | Non-marine | 8.49E-05 | 0.353 | 0.001 |
| high | Terrestrial | 9.36E-05 | 0.278 | 0.004 |
| low | Herbivory (Tree)*Non-marine | -1.81E-05 | 0.372 | 0.777 |
| low | Herbivory (Tree)*Terrestrial | -1.84E-05 | 0.263 | 0.833 |
| low | Herbivory (Scenario 1)*Non-marine | -1.78E-05 | 0.371 | 0.809 |
| low | Herbivory (Scenario 1)*Terrestrial | -2.32E-05 | 0.264 | 0.801 |
| low | Herbivory (Scenario 2)*Non-marine | -1.36E-05 | 0.371 | 0.853 |
| low | Herbivory (Scenario 2)*Terrestrial | -1.70E-05 | 0.263 | 0.854 |
| med | Herbivory (Tree)*Non-marine | -1.27E-05 | 0.368 | 0.837 |
| med | Herbivory (Tree)*Terrestrial | -1.40E-05 | 0.268 | 0.867 |
| med | Herbivory (Scenario 1)*Non-marine | -1.27E-05 | 0.367 | 0.858 |
| med | Herbivory (Scenario 1)*Terrestrial | -1.95E-05 | 0.270 | 0.825 |
| med | Herbivory (Scenario 2)*Non-marine | -8.13E-06 | 0.367 | 0.909 |
| med | Herbivory (Scenario 2)*Terrestrial | -1.31E-05 | 0.268 | 0.882 |
| high | Herbivory (Tree)*Non-marine | -7.49E-07 | 0.353 | 0.989 |
| high | Herbivory (Tree)*Terrestrial | -4.46E-06 | 0.280 | 0.953 |
| high | Herbivory (Scenario 1)*Non-marine | -6.41E-07 | 0.353 | 0.992 |
| high | Herbivory (Scenario 1)*Terrestrial | -1.07E-05 | 0.281 | 0.893 |
| high | Herbivory (Scenario 2)*Non-marine | 4.79E-06 | 0.353 | 0.941 |
| high | Herbivory (Scenario 2)*Terrestrial | -3.85E-06 | 0.279 | 0.961 |

**Table S12.** Estimated diversification rates for 28 animal phyla, based on Tree II. Diversification rate estimates (from Wiens 2015a) are based on three different relative extinction fractions (ε).

| Phylum | ε = 0 | ε = 0.5 | ε = 0.9 |
| --- | --- | --- | --- |
| Acoela | 0.0091709 | 0.0081225 | 0.0057084 |
| Annelida | 0.0167623 | 0.0155774 | 0.0128265 |
| Arthropoda | 0.0244300 | 0.0232242 | 0.0204246 |
| Brachiopoda | 0.0113725 | 0.0100583 | 0.0070338 |
| Bryozoa | 0.0149539 | 0.0137634 | 0.0110007 |
| Chaetognatha | 0.0071632 | 0.0061559 | 0.0038776 |
| Chordata | 0.0171470 | 0.0160807 | 0.0136048 |
| Cnidaria | 0.0120706 | 0.0111897 | 0.0091449 |
| Ctenophora | 0.0059717 | 0.0051865 | 0.0033968 |
| Echinodermata | 0.0163751 | 0.0150992 | 0.0121380 |
| Entoprocta | 0.0088632 | 0.0076822 | 0.0049940 |
| Gastrotricha | 0.0132172 | 0.0118522 | 0.0086963 |
| Gnathostomulida | 0.0100998 | 0.0085922 | 0.0052122 |
| Hemichordata | 0.0089618 | 0.0076998 | 0.0048457 |
| Kinorhyncha | 0.0100483 | 0.0087258 | 0.0057117 |
| Mollusca | 0.0186133 | 0.0174612 | 0.0147862 |
| Nematoda | 0.0254797 | 0.0237360 | 0.0196879 |
| Nematomorpha | 0.0146568 | 0.0129205 | 0.0089300 |
| Nemertea | 0.0129914 | 0.0117444 | 0.0088565 |
| Onychophora | 0.0090619 | 0.0078656 | 0.0051401 |
| Phoronida | 0.0052760 | 0.0040724 | 0.0017436 |
| Placozoa | 0.0000000 | 0.0000000 | 0.0000000 |
| Platyhelminthes | 0.0203042 | 0.0189367 | 0.0157621 |
| Porifera | 0.0108183 | 0.0099898 | 0.0080669 |
| Priapulida | 0.0056617 | 0.0044275 | 0.0019798 |
| Rotifera | 0.0178501 | 0.0163205 | 0.0127727 |
| Tardigrada | 0.0121065 | 0.0109197 | 0.0081724 |
| Xenoturbellida | 0.0010520 | 0.0006154 | 0.0001447 |

**Table S13.** Results of PGLS analyses of the relationship between diet (proportion of herbivorous species) and net diversification rates among 28 animal phyla based on Tree II. Net diversification rates were analyzed under three relative extinction fractions (ε = 0 [low]; 0.5 [medium], and 0.9 [high]; Table S12). We present results for three different estimates of the frequency of herbivory within each of the analyzed phylum. First, an estimate based on Dataset S1, using the species included in the tree (“Tree”). Second, based on estimates across all species for each phylum (see Supplementary Methods), including estimates assuming a low frequency of herbivory (Scenario 1) and a high frequency of herbivory (Scenario 2). Details on each of these herbivory scenarios are provided in Table S1. We also evaluated models accounting for the effect of habitat (non-marine [terrestrial and freshwater] vs. marine, and terrestrial vs. aquatic [marine and freshwater]), and its interaction with diet. The likelihood optimization for PGLS failed for ε=0.5, and ordinary least-squares regression was used instead. Diversification rates for each phylum are given in Table S12, and diet and habitat data are given in Table S10. Significant PGLS models are highlighted in gray.

| Epsilon | Predictor(s) | Slope | *r*^2^ | *P* |
| --- | --- | --- | --- | --- |
| low | Herbivory (Tree) | -9.10E-04 | 0.004 | 0.740 |
| low | Herbivory (Scenario 1) | -1.55E-03 | 0.011 | 0.595 |
| low | Herbivory (Scenario 2) | -1.31E-03 | 0.008 | 0.655 |
| low | Non-marine | 9.91E-05 | 0.369 | 0.001 |
| low | Terrestrial | 1.04E-04 | 0.262 | 0.005 |
| med | Non-marine | 9.52E-05 | 0.366 | 0.001 |
| med | Terrestrial | 1.01E-04 | 0.267 | 0.005 |
| high | Herbivory (Tree) | -1.01E-03 | 0.006 | 0.685 |
| high | Herbivory (Scenario 1) | -1.38E-03 | 0.011 | 0.602 |
| high | Herbivory (Scenario 2) | -1.14E-03 | 0.007 | 0.667 |
| high | Non-marine | 8.49E-05 | 0.353 | 0.001 |
| high | Terrestrial | 9.36E-05 | 0.278 | 0.004 |
| low | Herbivory (Tree)*Non-marine | -1.81E-05 | 0.372 | 0.777 |
| low | Herbivory (Scenario 1)*Non-marine | -1.78E-05 | 0.371 | 0.809 |
| low | Herbivory (Scenario 2)*Non-marine | -1.36E-05 | 0.371 | 0.853 |
| med | Herbivory (Tree)*Non-marine | -1.27E-05 | 0.368 | 0.837 |
| med | Herbivory (Tree)*Terrestrial | -1.40E-05 | 0.268 | 0.867 |
| med | Herbivory (Scenario 1)*Non-marine | -1.27E-05 | 0.367 | 0.858 |
| med | Herbivory (Scenario 1)*Terrestrial | -1.95E-05 | 0.270 | 0.825 |
| med | Herbivory (Scenario 2)*Non-marine | -8.13E-06 | 0.367 | 0.909 |
| med | Herbivory (Scenario 2)*Terrestrial | -1.31E-05 | 0.268 | 0.882 |
| high | Herbivory (Tree)*Non-marine | -7.49E-07 | 0.353 | 0.989 |
| high | Herbivory (Tree)*Terrestrial | -4.46E-06 | 0.280 | 0.953 |
| high | Herbivory (Scenario 1)*Non-marine | -6.41E-07 | 0.353 | 0.992 |
| high | Herbivory (Scenario 1)*Terrestrial | -1.07E-05 | 0.281 | 0.893 |
| high | Herbivory (Scenario 2)*Non-marine | 4.79E-06 | 0.353 | 0.941 |
| high | Herbivory (Scenario 2)*Terrestrial | -3.85E-06 | 0.279 | 0.961 |

**Table S14.** Estimated net diversification rates for animal phyla for Tree III. Estimates are from Wiens (2015a) and are based on three different relative extinction fractions (ε).

| Phylum | ε = 0 | ε = 0.5 | ε = 0.9 |
| --- | --- | --- | --- |
| Acoela | 0.00832478 | 0.00737312 | 0.00518171 |
| Annelida | 0.01737672 | 0.01614833 | 0.01329666 |
| Arthropoda | 0.02405359 | 0.02286644 | 0.02010997 |
| Brachiopoda | 0.01408248 | 0.01245515 | 0.00870996 |
| Bryozoa | 0.01527196 | 0.01405611 | 0.01123464 |
| Chaetognatha | 0.00759499 | 0.00652699 | 0.00411134 |
| Chordata | 0.01614028 | 0.01513653 | 0.01280601 |
| Cnidaria | 0.01216028 | 0.01127287 | 0.00921291 |
| Ctenophora | 0.0049605 | 0.00430827 | 0.0028216 |
| Echinodermata | 0.01752657 | 0.01616092 | 0.01299151 |
| Entoprocta | 0.00905168 | 0.00784559 | 0.00510022 |
| Gastrotricha | 0.01124555 | 0.01008412 | 0.00739901 |
| Gnathostomulida | 0.00779949 | 0.00663522 | 0.00402506 |
| Hemichordata | 0.00959201 | 0.00824119 | 0.00518643 |
| Kinorhyncha | 0.01000767 | 0.00869051 | 0.00568855 |
| Mollusca | 0.01905489 | 0.01787547 | 0.01513705 |
| Nematoda | 0.02548142 | 0.02373761 | 0.01968918 |
| Nematomorpha | 0.01465781 | 0.01292131 | 0.00893059 |
| Nemertea | 0.01370043 | 0.0123854 | 0.00933989 |
| Onychophora | 0.00892227 | 0.00774446 | 0.00506087 |
| Phoronida | 0.00653324 | 0.00504278 | 0.00215912 |
| Placozoa | 0 | 0 | 0 |
| Platyhelminthes | 0.01636488 | 0.01526276 | 0.01270402 |
| Porifera | 0.01102671 | 0.01018226 | 0.00822231 |
| Priapulida | 0.00563881 | 0.00440961 | 0.00197179 |
| Rotifera | 0.01378454 | 0.01260331 | 0.00986355 |
| Tardigrada | 0.01387106 | 0.01251131 | 0.00936357 |
| Xenoturbellida | 0.00114678 | 0.00067082 | 0.00015769 |

**Table S15.** Results of PGLS analyses of the relationship between diet (proportion of herbivorous species) and net diversification rates among 28 animal phyla based on Tree III. Net diversification rates were analyzed under three relative extinction fractions (ε = 0 [low]; 0.5 [medium], and 0.9 [high]; Table S14). We present results for three different estimates of the frequency of herbivory within each of the analyzed phylum. First, an estimate based on Dataset S1, using the species included in the tree (“Tree”). Second, based on estimates across all species for each phylum (see Supplementary Methods), including estimates assuming a low frequency of herbivory (Scenario 1) and a high frequency of herbivory (Scenario 2). Details on each of these herbivory scenarios are provided in Table S1. We also evaluated models accounting for the effect of habitat (non-marine [terrestrial and freshwater] vs. marine, and terrestrial vs. aquatic [marine and freshwater]), and its interaction with diet. Diversification rates for each phylum are given in Table S14, and diet and habitat data are given in Table S10. Significant PGLS models are highlighted in gray.

| Epsilon | Predictor(s) | Slope | *r*^2^ | *P* |
| --- | --- | --- | --- | --- |
| low | Herbivory (Tree) | 3.55E-05 | 0.000 | 0.999 |
| low | Herbivory (Scenario 1) | -2.36E-04 | 0.000 | 0.936 |
| low | Herbivory (Scenario 2) | -1.50E-05 | 0.000 | 0.996 |
| low | Non-marine | 8.77E-05 | 0.306 | 0.002 |
| low | Terrestrial | 9.90E-05 | 0.253 | 0.006 |
| med | Herbivory (Tree) | -2.11E-04 | 0.000 | 0.937 |
| med | Herbivory (Scenario 1) | -3.84E-04 | 0.001 | 0.892 |
| med | Herbivory (Scenario 2) | -1.61E-04 | 0.000 | 0.955 |
| med | Non-marine | 8.48E-05 | 0.308 | 0.002 |
| med | Terrestrial | 9.67E-05 | 0.259 | 0.006 |
| high | Herbivory (Tree) | -7.60E-04 | 0.004 | 0.753 |
| high | Herbivory (Scenario 1) | -7.58E-04 | 0.003 | 0.768 |
| high | Herbivory (Scenario 2) | -5.34E-04 | 0.002 | 0.837 |
| high | Non-marine | 7.67E-05 | 0.304 | 0.002 |
| high | Terrestrial | 9.00E-05 | 0.271 | 0.004 |
| low | Herbivory (Tree)*Non-marine | -3.56E-05 | 0.319 | 0.583 |
| low | Herbivory (Tree)*Terrestrial | -1.70E-05 | 0.255 | 0.842 |
| low | Herbivory (Scenario 1)*Non-marine | -2.07E-05 | 0.316 | 0.780 |
| low | Herbivory (Scenario 1)*Terrestrial | -6.99E-06 | 0.254 | 0.938 |
| low | Herbivory (Scenario 2)*Non-marine | -1.65E-05 | 0.316 | 0.824 |
| low | Herbivory (Scenario 2)*Terrestrial | -1.10E-06 | 0.255 | 0.990 |
| med | Herbivory (Tree)*Non-marine | -2.77E-05 | 0.315 | 0.658 |
| med | Herbivory (Tree)*Terrestrial | -1.21E-05 | 0.260 | 0.882 |
| med | Herbivory (Scenario 1)*Non-marine | -1.47E-05 | 0.314 | 0.838 |
| med | Herbivory (Scenario 1)*Terrestrial | -4.42E-06 | 0.260 | 0.959 |
| med | Herbivory (Scenario 2)*Non-marine | -1.01E-05 | 0.314 | 0.888 |
| med | Herbivory (Scenario 2)*Terrestrial | 1.69E-06 | 0.260 | 0.984 |
| high | Herbivory (Tree)*Non-marine | -1.05E-05 | 0.305 | 0.855 |
| high | Herbivory (Tree)*Terrestrial | -1.76E-06 | 0.272 | 0.981 |
| high | Herbivory (Scenario 1)*Non-marine | -4.94E-07 | 0.305 | 0.994 |
| high | Herbivory (Scenario 1)*Terrestrial | 1.78E-06 | 0.271 | 0.982 |
| high | Herbivory (Scenario 2)*Non-marine | 4.91E-06 | 0.306 | 0.940 |
| high | Herbivory (Scenario 2)*Terrestrial | 8.34E-06 | 0.272 | 0.915 |

**Table S16.** Estimated diversification rates for 28 animal phyla based on projected species richness per phylum. Diversification rates are estimated using three different relative extinction fractions (ε = 0, 0.5, and 0.9). However, instead of using the known, described species richness within each phylum, we used the projected richness (Table S1). Rate estimates are based on the three different tree topologies. Diversification rates are estimated under two different scenarios for projected species richness (projected low and high richness per phylum; see Table S1 for richness values).

| Phylum | Projected low richness | | | | | | | | | Projected high richness | | | | | | | | |
| --- | --- | --- | --- | --- | --- | --- | --- | --- | --- | --- | --- | --- | --- | --- | --- | --- | --- | --- |
|  | Tree I | | | Tree II | | | Tree III | | | Tree I | | | Tree II | | | Tree III | | |
|  | ε=0 | ε=0.5 | ε=0.9 | ε=0 | ε=0.5 | ε=0.9 | ε=0 | ε=0.5 | ε=0.9 | ε=0 | ε=0.5 | ε=0.9 | ε=0 | ε=0.5 | ε=0.9 | ε=0 | ε=0.5 | ε=0.9 |
| Acoela | 0.0200 | 0.0188 | 0.0162 | 0.0203 | 0.0191 | 0.0165 | 0.0208 | 0.0196 | 0.0169 | 0.0200 | 0.0188 | 0.0162 | 0.0203 | 0.0191 | 0.0165 | 0.0208 | 0.0196 | 0.0169 |
| Annelida | 0.0288 | 0.0275 | 0.0247 | 0.0286 | 0.0274 | 0.0246 | 0.0281 | 0.0269 | 0.0242 | 0.0322 | 0.0310 | 0.0282 | 0.0320 | 0.0308 | 0.0280 | 0.0315 | 0.0303 | 0.0276 |
| Arthropoda | 0.0161 | 0.0149 | 0.0120 | 0.0158 | 0.0146 | 0.0119 | 0.0161 | 0.0149 | 0.0121 | 0.0161 | 0.0149 | 0.0120 | 0.0158 | 0.0146 | 0.0119 | 0.0161 | 0.0149 | 0.0121 |
| Brachiopoda | 0.0181 | 0.0169 | 0.0140 | 0.0176 | 0.0164 | 0.0137 | 0.0183 | 0.0170 | 0.0142 | 0.0181 | 0.0169 | 0.0140 | 0.0176 | 0.0164 | 0.0137 | 0.0183 | 0.0170 | 0.0142 |
| Bryozoa | 0.0178 | 0.0165 | 0.0136 | 0.0178 | 0.0166 | 0.0137 | 0.0188 | 0.0175 | 0.0144 | 0.0178 | 0.0165 | 0.0136 | 0.0178 | 0.0166 | 0.0137 | 0.0188 | 0.0175 | 0.0144 |
| Chaetognatha | 0.0205 | 0.0192 | 0.0163 | 0.0223 | 0.0209 | 0.0177 | 0.0180 | 0.0168 | 0.0143 | 0.0205 | 0.0192 | 0.0163 | 0.0223 | 0.0209 | 0.0177 | 0.0180 | 0.0168 | 0.0143 |
| Chordata | 0.0093 | 0.0081 | 0.0053 | 0.0100 | 0.0087 | 0.0057 | 0.0100 | 0.0087 | 0.0057 | 0.0093 | 0.0081 | 0.0053 | 0.0100 | 0.0087 | 0.0057 | 0.0100 | 0.0087 | 0.0057 |
| Cnidaria | 0.0094 | 0.0082 | 0.0055 | 0.0094 | 0.0082 | 0.0054 | 0.0092 | 0.0081 | 0.0054 | 0.0094 | 0.0082 | 0.0055 | 0.0094 | 0.0082 | 0.0054 | 0.0092 | 0.0081 | 0.0054 |
| Ctenophora | 0.0122 | 0.0107 | 0.0074 | 0.0127 | 0.0112 | 0.0077 | 0.0098 | 0.0086 | 0.0059 | 0.0122 | 0.0107 | 0.0074 | 0.0127 | 0.0112 | 0.0077 | 0.0098 | 0.0086 | 0.0059 |
| Echinodermata | 0.0156 | 0.0146 | 0.0124 | 0.0174 | 0.0163 | 0.0138 | 0.0164 | 0.0154 | 0.0130 | 0.0156 | 0.0146 | 0.0124 | 0.0174 | 0.0163 | 0.0138 | 0.0164 | 0.0154 | 0.0130 |
| Entoprocta | 0.0111 | 0.0103 | 0.0085 | 0.0123 | 0.0114 | 0.0094 | 0.0124 | 0.0115 | 0.0094 | 0.0111 | 0.0103 | 0.0085 | 0.0123 | 0.0114 | 0.0094 | 0.0124 | 0.0115 | 0.0094 |
| Gastrotricha | 0.0121 | 0.0110 | 0.0082 | 0.0123 | 0.0111 | 0.0084 | 0.0141 | 0.0127 | 0.0096 | 0.0121 | 0.0110 | 0.0082 | 0.0123 | 0.0111 | 0.0084 | 0.0141 | 0.0127 | 0.0096 |
| Gnathostomulida | 0.0058 | 0.0052 | 0.0036 | 0.0069 | 0.0061 | 0.0043 | 0.0057 | 0.0050 | 0.0035 | 0.0058 | 0.0052 | 0.0036 | 0.0069 | 0.0061 | 0.0043 | 0.0057 | 0.0050 | 0.0035 |
| Hemichordata | 0.0124 | 0.0112 | 0.0084 | 0.0122 | 0.0110 | 0.0083 | 0.0125 | 0.0113 | 0.0084 | 0.0124 | 0.0112 | 0.0084 | 0.0122 | 0.0110 | 0.0083 | 0.0125 | 0.0113 | 0.0084 |
| Kinorhyncha | 0.0172 | 0.0157 | 0.0123 | 0.0179 | 0.0163 | 0.0128 | 0.0138 | 0.0126 | 0.0099 | 0.0172 | 0.0157 | 0.0123 | 0.0179 | 0.0163 | 0.0128 | 0.0138 | 0.0126 | 0.0099 |
| Mollusca | 0.0055 | 0.0043 | 0.0019 | 0.0055 | 0.0043 | 0.0019 | 0.0068 | 0.0053 | 0.0023 | 0.0055 | 0.0043 | 0.0019 | 0.0055 | 0.0043 | 0.0019 | 0.0068 | 0.0053 | 0.0023 |
| Nematoda | 0.0048 | 0.0040 | 0.0023 | 0.0054 | 0.0046 | 0.0027 | 0.0055 | 0.0046 | 0.0027 | 0.0048 | 0.0040 | 0.0023 | 0.0054 | 0.0046 | 0.0027 | 0.0055 | 0.0046 | 0.0027 |
| Nematomorpha | 0.0052 | 0.0041 | 0.0018 | 0.0057 | 0.0044 | 0.0020 | 0.0056 | 0.0044 | 0.0020 | 0.0052 | 0.0041 | 0.0018 | 0.0057 | 0.0044 | 0.0020 | 0.0056 | 0.0044 | 0.0020 |
| Nemertea | 0.0093 | 0.0080 | 0.0050 | 0.0090 | 0.0077 | 0.0048 | 0.0096 | 0.0082 | 0.0052 | 0.0093 | 0.0080 | 0.0050 | 0.0090 | 0.0077 | 0.0048 | 0.0096 | 0.0082 | 0.0052 |
| Onychophora | 0.0082 | 0.0072 | 0.0050 | 0.0085 | 0.0075 | 0.0052 | 0.0090 | 0.0080 | 0.0055 | 0.0082 | 0.0072 | 0.0050 | 0.0085 | 0.0075 | 0.0052 | 0.0090 | 0.0080 | 0.0055 |
| Phoronida | 0.0147 | 0.0129 | 0.0089 | 0.0147 | 0.0129 | 0.0089 | 0.0147 | 0.0129 | 0.0089 | 0.0147 | 0.0129 | 0.0089 | 0.0147 | 0.0129 | 0.0089 | 0.0147 | 0.0129 | 0.0089 |
| Placozoa | 0.0182 | 0.0169 | 0.0138 | 0.0176 | 0.0163 | 0.0133 | 0.0188 | 0.0174 | 0.0143 | 0.0182 | 0.0169 | 0.0138 | 0.0176 | 0.0163 | 0.0133 | 0.0188 | 0.0174 | 0.0143 |
| Platyhelminthes | 0.0111 | 0.0103 | 0.0086 | 0.0122 | 0.0113 | 0.0094 | 0.0124 | 0.0116 | 0.0096 | 0.0111 | 0.0103 | 0.0086 | 0.0122 | 0.0113 | 0.0094 | 0.0124 | 0.0116 | 0.0096 |
| Porifera | 0.0118 | 0.0108 | 0.0085 | 0.0126 | 0.0116 | 0.0091 | 0.0115 | 0.0105 | 0.0083 | 0.0118 | 0.0108 | 0.0085 | 0.0126 | 0.0116 | 0.0091 | 0.0115 | 0.0105 | 0.0083 |
| Priapulida | 0.0114 | 0.0101 | 0.0071 | 0.0114 | 0.0101 | 0.0070 | 0.0141 | 0.0125 | 0.0087 | 0.0114 | 0.0101 | 0.0071 | 0.0114 | 0.0101 | 0.0070 | 0.0141 | 0.0125 | 0.0087 |
| Rotifera | 0.0396 | 0.0378 | 0.0338 | 0.0396 | 0.0378 | 0.0338 | 0.0396 | 0.0378 | 0.0338 | 0.0441 | 0.0423 | 0.0383 | 0.0441 | 0.0423 | 0.0383 | 0.0441 | 0.0423 | 0.0383 |
| Tardigrada | 0.0144 | 0.0131 | 0.0102 | 0.0156 | 0.0143 | 0.0111 | 0.0133 | 0.0121 | 0.0094 | 0.0144 | 0.0131 | 0.0102 | 0.0156 | 0.0143 | 0.0111 | 0.0133 | 0.0121 | 0.0094 |
| Xenoturbellida | 0.0010 | 0.0006 | 0.0001 | 0.0011 | 0.0006 | 0.0001 | 0.0011 | 0.0007 | 0.0002 | 0.0010 | 0.0006 | 0.0001 | 0.0011 | 0.0006 | 0.0001 | 0.0011 | 0.0007 | 0.0002 |

**Table S17.** Results of PGLS analyses of the relationship between diet (proportion of herbivorous species) and net diversification rates based on projected richness (lowest projections) among 28 animal phyla based on Trees I–III. Net diversification rates were analyzed under three relative extinction fractions (ε = 0 [low]; 0.5 [med], and 0.9 [high]; Table S16) and based on the lower estimate of projected richness per phylum (Table S1). We present results for two different datasets describing the frequency of herbivory within each phylum. Except for arthropods, the frequency of herbivory is estimated based on a scenario with a low frequency of herbivory (Scenario 1) or a high frequency (Scenario 2). These two scenarios are summarized in Table S1. For arthropods, we analyzed the lowest projected frequency of herbivory (corresponding to the lowest projected richness), as summarized in Table S1 (projected frequency=0.191). Diversification rates for each phylum are given in Table S16. Significant PGLS results are highlighted in gray.

| Tree number | Epsilon | Predictor | Slope | *r^2^* | *P* |
| --- | --- | --- | --- | --- | --- |
| TreeI | low | Herbivory (Scenario 1) | 5.26E-03 | 0.076 | 0.157 |
| TreeI | low | Herbivory (Scenario 2) | 5.32E-03 | 0.076 | 0.155 |
| TreeI | med | Herbivory (Scenario 1) | 5.04E-03 | 0.073 | 0.164 |
| TreeI | med | Herbivory (Scenario 2) | 5.09E-03 | 0.073 | 0.163 |
| TreeI | high | Herbivory (Scenario 1) | 4.43E-03 | 0.064 | 0.195 |
| TreeI | high | Herbivory (Scenario 2) | 4.47E-03 | 0.063 | 0.196 |
| TreeII | low | Herbivory (Scenario 1) | 4.93E-03 | 0.068 | 0.182 |
| TreeII | low | Herbivory (Scenario 2) | 4.87E-03 | 0.065 | 0.189 |
| TreeII | med | Herbivory (Scenario 1) | 4.76E-03 | 0.066 | 0.188 |
| TreeII | med | Herbivory (Scenario 2) | 4.70E-03 | 0.063 | 0.196 |
| TreeII | high | Herbivory (Scenario 1) | 4.27E-03 | 0.059 | 0.213 |
| TreeII | high | Herbivory (Scenario 2) | 4.19E-03 | 0.056 | 0.223 |
| TreeIII | low | Herbivory (Scenario 1) | 6.26E-03 | 0.114 | 0.079 |
| TreeIII | low | Herbivory (Scenario 2) | 6.22E-03 | 0.112 | 0.082 |
| TreeIII | med | Herbivory (Scenario 1) | 5.97E-03 | 0.109 | 0.087 |
| TreeIII | med | Herbivory (Scenario 2) | 5.92E-03 | 0.106 | 0.091 |
| TreeIII | high | Herbivory (Scenario 1) | 5.28E-03 | 0.094 | 0.112 |
| TreeIII | high | Herbivory (Scenario 2) | 5.23E-03 | 0.091 | 0.118 |

**Table S18.** Results of PGLS analyses of the relationship between diet (proportion of herbivorous species) and net diversification rates based on projected richness (highest projections) among 28 animal phyla based on Trees I–III. Net diversification rates were analyzed under three relative extinction fractions (ε = 0 [low]; 0.5 [med], and 0.9 [high]; Table S16) and based on the highest projected richness per phylum (Table S1). We present results for two different datasets describing the frequency of herbivory within each phylum. Except for arthropods, the frequency of herbivory is estimated based on a scenario with a low frequency of herbivory (Scenario 1) or a high frequency (Scenario 2). These two scenarios are summarized in Table S1. For arthropods, we analyzed the highest projected frequency of herbivory (corresponding to the highest projected richness), as summarized in Table S1 (projected frequency=0.325). Diversification rates for each phylum are given in Table S16. Significant PGLS results are highlighted in gray.

| Tree number | Diversification rate | Predictor | Slope | *r^2^* | *P* |
| --- | --- | --- | --- | --- | --- |
| TreeI | low | Herbivory (Scenario 1) | 5.40E-03 | 0.064 | 0.192 |
| TreeI | low | Herbivory (Scenario 2) | 5.33E-03 | 0.062 | 0.200 |
| TreeI | med | Herbivory (Scenario 1) | 5.18E-03 | 0.062 | 0.202 |
| TreeI | med | Herbivory (Scenario 2) | 5.11E-03 | 0.060 | 0.210 |
| TreeI | high | Herbivory (Scenario 1) | 4.56E-03 | 0.053 | 0.238 |
| TreeI | high | Herbivory (Scenario 2) | 4.48E-03 | 0.051 | 0.249 |
| TreeII | low | Herbivory (Scenario 1) | 5.94E-03 | 0.080 | 0.145 |
| TreeII | low | Herbivory (Scenario 2) | 5.88E-03 | 0.078 | 0.150 |
| TreeII | med | Herbivory (Scenario 1) | 5.75E-03 | 0.078 | 0.151 |
| TreeII | med | Herbivory (Scenario 2) | 5.69E-03 | 0.076 | 0.157 |
| TreeII | high | Herbivory (Scenario 1) | 5.22E-03 | 0.070 | 0.172 |
| TreeII | high | Herbivory (Scenario 2) | 5.15E-03 | 0.068 | 0.180 |
| TreeIII | low | Herbivory (Scenario 1) | 5.65E-03 | 0.074 | 0.161 |
| TreeIII | low | Herbivory (Scenario 2) | 5.60E-03 | 0.072 | 0.167 |
| TreeIII | med | Herbivory (Scenario 1) | 5.38E-03 | 0.070 | 0.175 |
| TreeIII | med | Herbivory (Scenario 2) | 5.32E-03 | 0.068 | 0.181 |
| TreeIII | high | Herbivory (Scenario 1) | 4.72E-03 | 0.059 | 0.214 |
| TreeIII | high | Herbivory (Scenario 2) | 4.65E-03 | 0.056 | 0.223 |

**Table S19.** Estimated diet for key nodes of the animal Tree of Life based on the best-fitting HiSSE model (M24; Tree I; Table S6). Values are proportional likelihoods of each observed state (herbivore: 0; carnivore: 1), summed across the two hidden states inferred for each observed state. Ancestral-state reconstructions used the default option for estimating the initial probabilities at the root for each state (maddfitz). Results are shown for each binary coding strategy (maxcar and maxherb). Speciation and extinction rates for this model are described in Table S6 and transition rates are given in Table S42. Estimates for all nodes are shown graphically in Figure 1 (maxcar) and Figure S1 (maxherb).

| Strategy | Clade | Herbivorous | Carnivorous |
| --- | --- | --- | --- |
| Maxcar | Root | 0.0000 | 1.0000 |
|  | Bilateria | 0.0191 | 0.9809 |
|  | Protostomia | 0.1351 | 0.8649 |
|  | Deuterostomia | 0.0000 | 1.0000 |
|  | Arthropoda | 0.0000 | 1.0000 |
|  | Chordata | 0.0198 | 0.9802 |
|  | Mollusca | 0.0047 | 0.9953 |
|  |  |  |  |
| Maxherb | Root | 0.0474 | 0.9526 |
|  | Bilateria | 0.1209 | 0.8791 |
|  | Protostomia | 0.1950 | 0.8050 |
|  | Deuterostomia | 0.3141 | 0.6859 |
|  | Arthropoda | 0.4467 | 0.5533 |
|  | Chordata | 0.0111 | 0.9889 |
|  | Mollusca | 0.0169 | 0.9831 |

**Table S20.** Estimated diet for key nodes of the animal Tree of Life based on the best-fitting HiSSE model (M24; Table S8) for Tree II. Values are proportional likelihoods of each observed state (herbivore: 0, carnivore: 1), summed across the hidden states inferred for each observed state. Ancestral-state reconstructions used the best-fitting option for estimating the initial probabilities at the root for each state (maddfitz). Results are shown for each coding strategy (maxcar and maxherb).

| Strategy | Clade | Herbivorous | Carnivorous |
| --- | --- | --- | --- |
| Maxcar | Root | 0.023 | 0.977 |
|  | Bilateria | 0.171 | 0.829 |
|  | Protostomia | 0.012 | 0.988 |
|  | Deuterostomia | 0.108 | 0.892 |
|  | Arthropoda | 0.000 | 1.000 |
|  | Chordata | 0.002 | 0.998 |
|  | Mollusca | 0.000 | 1.000 |
|  |  |  |  |
| Maxherb | Root | 0.149 | 0.851 |
|  | Bilateria | 0.006 | 0.994 |
|  | Protostomia | 0.007 | 0.993 |
|  | Deuterostomia | 0.003 | 0.997 |
|  | Arthropoda | 0.035 | 0.965 |
|  | Chordata | 0.027 | 0.973 |
|  | Mollusca | 0.047 | 0.953 |

**Table S21.** Estimated diet for key nodes of the animal Tree of Life based on the best-fitting HiSSE model for Tree III. The best fitting model is M24: see Table S9 for details. Values are proportional likelihoods of each observed state (herbivore: 0, carnivore: 1), summed across the hidden states inferred for each observed state. Ancestral-state reconstructions used the best-fitting option for estimating the initial probabilities at the root for each state (maddfitz). Results are shown for each coding strategy (maxcar and maxherb).

| Coding | Clade | Herbivorous | Carnivorous |
| --- | --- | --- | --- |
| Maxcar | Root | 0.088 | 0.912 |
|  | Bilateria | 0.090 | 0.910 |
|  | Deuterostomia | 0.049 | 0.951 |
|  | Arthropoda | 0.000 | 1.000 |
|  | Chordata | 0.006 | 0.994 |
|  | Mollusca | 0.000 | 1.000 |
|  |  |  |  |
| Maxherb | Root | 0.092 | 0.908 |
|  | Bilateria | 0.091 | 0.909 |
|  | Deuterostomia | 0.115 | 0.885 |
|  | Arthropoda | 0.012 | 0.988 |
|  | Chordata | 0.005 | 0.995 |
|  | Mollusca | 0.122 | 0.878 |

**Table 22.** Comparison of the fit of different likelihood models for the evolution of animal diet (using HiSSE) for Trees I–III, using an alternative dataset for diet. Analyses were based on a matrix in which states for four phyla were modified to better reflect estimates of diet based on all species in each phylum (Dataset S3). Given that HiSSE allows only two states per character, omnivorous species (or taxa with ambiguous states) were either coded as carnivorous (state 1; maxcar) in one set of analyses, or as herbivorous in the other (state 0; maxherb). For each coding approach, we fit a total of five SSE models, including a BiSSE model, two standard null HiSSE models, an alternative HiSSE null model (i.e. M24; see Table S6), and a full HiSSE model. These models were used for analyzing trait evolution and state-dependent diversification across the tree. Observed states were coded as 0 (herbivorous) or 1 (carnivorous), and the hidden states as A or B. The default (maddfitz) option was used for estimating the initial probabilities of each state at the root. Log-likelihood (i.e., natural logarithm; lnL) and AICc values are shown for each model. We fitted a full BiSSE model, with independent diversification parameters between the two observed states (i.e. 0, 1). The two standard null models have effectively same number of variable parameters as a BiSSE-like model (i.e. “Null-two”) or HiSSE-like model (i.e. “Null-four”). However, in the “null-two” model there are different speciation and different extinction rates for each of the hidden states (A or B) but speciation and extinction rates are constrained to be equal between the observed states (0, 1). “Null-four” includes a total of four hidden states (A, B, C, D) within each observed state (0, 1). Each hidden state had different speciation rates and different extinction rates, but speciation and extinction rates were constrained to be equal between the observed states. Thus, for the two null models, there is no effect of the observed state on diversification. Next, M24 included all states but disallowed 1A-0B and 1B-0A state transitions. This model is also similar to the null models, except that in the “standard” null models the transition rates are constrained to be equal between states, whereas in the M24 models transition rates were allowed to be different. Finally, the full HiSSE includes independent diversification parameters for each hidden state, and allows all transitions between states. ΔΑΙCc relative to the best-fitting model (M24 in this case). The best-fitting model (∆AIC<4) is boldfaced.

| Model | Included states | Transition matrix | | λ | μ | Tree 1 | | | | Tree 2 | | | | Tree 3 | | | |
| --- | --- | --- | --- | --- | --- | --- | --- | --- | --- | --- | --- | --- | --- | --- | --- | --- | --- |
|  |  |  |  |  |  | Maxcar | | Maxherb | | Maxcar | | Maxherb | | Maxcar | | Maxherb | |
|  |  | Structure* | Rates |  |  | lnL | ΔΑΙCc | lnL | ΔΑΙCc | lnL | ΔΑΙCc | lnL | ΔΑΙCc | lnL | ΔΑΙCc | lnL | ΔΑΙCc |
| BiSSE | 0,1 | All transitions included | Different | 0≠1 | 0≠1 | -7094.91 | 649.03 | -7155.84 | 702.91 | -7166.24 | 742.35 | -7165.17 | 689.54 | -7158.53 | 743.24 | -7159.49 | 700.06 |
| Null-two | 0A,0B,1A,1B | 1B-0A, 0B-1A not allowed | All equal | 0A=1A; 0B=1B | 0A=1A; 0B=1B | -7050.40 | 562.06 | -7054.69 | 502.65 | -7073.13 | 558.19 | -7059.76 | 480.77 | -7072.13 | 572.49 | -7055.46 | 494.05 |
| Null-four | 0A,0B,1A,1B | All transitions included | All equal | 1A=0A; 0B=1B; 0C=1C; 0D=1D | 1A=0A; 0B=1B; 0C=1C; 0D=1D | -6953.54 | 376.46 | -6974.69 | 350.76 | -6959.52 | 339.07 | -6979.51 | 328.38 | -6957.51 | 351.35 | -6976.24 | 343.72 |
| M24 | **0A,0B,1A,1B** | **1A-0B, 1B-0A not allowed** | **Different** | **0A=1A; 0B=1B** | **0A=1A; 0B=1B** | **-6772.25** | **0.00** | **-6796.25** | **0.00** | **-6786.92** | **0.00** | **-6812.26** | **0.00** | **-6778.77** | **0.00** | **-6806.32** | **0.00** |
| Full HiSSE | 0A,0B,1A,1B | All transitions included | Different | 0A≠1A≠0B≠1B | 0A≠1A≠0B≠1B | -6762.20 | 16.39 | -6799.38 | 22.76 | -6790.61 | 23.87 | -6810.34 | 12.68 | -6780.05 | 19.05 | -6797.32 | 8.50 |

**Table S23.** Estimated diet for key nodes of the animal Tree of Life based on the best-fitting HiSSE model for Tree I, using a modified diet dataset. Analyses were based on a matrix in which states for four phyla were modified to better reflect estimates of diet based on all species in each phylum (Dataset S3). The best fitting model is M24: see Table S22 for details. Values are proportional likelihoods of each observed state (herbivore: 0, carnivore: 1), summed across the hidden states inferred for each observed state. Ancestral-state reconstructions used the best-fitting option for estimating the initial probabilities at the root for each state (maddfitz). Results are shown for each coding strategy (maxcar and maxherb).

| Coding | Clade | Herbivorous | Carnivorous |
| --- | --- | --- | --- |
| Maxcar | Root | 0.028 | 0.972 |
|  | Bilateria | 0.020 | 0.980 |
|  | Protostomia | 0.045 | 0.955 |
|  | Deuterostomia | 0.036 | 0.964 |
|  | Arthropoda | 0.183 | 0.817 |
|  | Chordata | 0.001 | 0.999 |
|  | Mollusca | 0.001 | 0.999 |
|  |  |  |  |
| Maxherb | Root | 0.040 | 0.960 |
|  | Bilateria | 0.007 | 0.993 |
|  | Protostomia | 0.009 | 0.991 |
|  | Deuterostomia | 0.005 | 0.995 |
|  | Arthropoda | 0.119 | 0.881 |
|  | Chordata | 0.032 | 0.968 |
|  | Mollusca | 0.028 | 0.972 |

**Table S24.** Estimated diet for key nodes of the animal Tree of Life based on the best-fitting HiSSE model for Tree II, using a modified diet dataset. Analyses were based on a matrix in which states for four phyla were modified to better reflect estimates of diet based on all species in each phylum (Dataset S3). The best fitting model is M24: see Table S22 for details. Values are proportional likelihoods of each observed state (herbivore: 0, carnivore: 1), summed across the hidden states inferred for each observed state. Ancestral-state reconstructions used the best-fitting option for estimating the initial probabilities at the root for each state (maddfitz). Results are shown for each coding strategy (maxcar and maxherb).

| Coding | Clade | Herbivorous | Carnivorous |
| --- | --- | --- | --- |
| Maxcar | Root | 0.028 | 0.972 |
|  | Bilateria | 0.020 | 0.980 |
|  | Protostomia | 0.163 | 0.837 |
|  | Deuterostomia | 0.036 | 0.964 |
|  | Arthropoda | 0.183 | 0.817 |
|  | Chordata | 0.001 | 0.999 |
|  | Mollusca | 0.001 | 0.999 |
|  |  |  |  |
| Maxherb | Root | 0.119 | 0.881 |
|  | Bilateria | 0.006 | 0.994 |
|  | Protostomia | 0.007 | 0.993 |
|  | Deuterostomia | 0.003 | 0.997 |
|  | Arthropoda | 0.035 | 0.965 |
|  | Chordata | 0.027 | 0.973 |
|  | Mollusca | 0.047 | 0.953 |

**Table S25.** Estimated diet for key nodes of the animal Tree of Life based on the best-fitting HiSSE model for Tree III, using a modified diet dataset. Analyses were based on a matrix in which states for four phyla were modified to better reflect estimates of diet based on all species in each phylum (Dataset S3). The best fitting model is M24: see Table S22 for details. Values are proportional likelihoods of each observed state (herbivore: 0, carnivore: 1), summed across the hidden states inferred for each observed state. Ancestral-state reconstructions used the best-fitting option for estimating the initial probabilities at the root for each state (maddfitz). Results are shown for each coding strategy (maxcar and maxherb).

| Coding | Clade | Herbivorous | Carnivorous |
| --- | --- | --- | --- |
| Maxcar | Root | 0.092 | 0.908 |
|  | Bilateria | 0.091 | 0.909 |
|  | Deuterostomia | 0.115 | 0.885 |
|  | Arthropoda | 0.012 | 0.988 |
|  | Chordata | 0.005 | 0.995 |
|  | Mollusca | 0.122 | 0.878 |
|  |  |  |  |
| Maxherb | Root | 0.105 | 0.895 |
|  | Bilateria | 0.079 | 0.921 |
|  | Deuterostomia | 0.018 | 0.982 |
|  | Arthropoda | 0.005 | 0.995 |
|  | Chordata | 0.000 | 1.000 |
|  | Mollusca | 0.087 | 0.913 |

**Table S26.** Comparison of the fit (AICc) for different corHMM models for the evolution of diet for Tree I. Models differ based on the number of hidden states (rate categories) within each observed state, from 0 to 4. The total number of hidden states is always twice the number of observed states because the number of hidden states per observed state must be equal for the two observed states (e.g. when there are two hidden states for carnivory, there must also be two hidden states for herbivory). Different hidden states differ based on their transition rates only, not differences in rates of speciation and extinction. Results are shown for the two coding strategies (maxcar and maxherb). The best-fitting model is boldfaced for each coding strategy. Transition rates correspond to the all-rates-different model (all transition between states are allowed to take a different rate, rather than being constrained to be equal).

| Hidden states per observed state | Maxcar | Maxherb |
| --- | --- | --- |
| 0 | 823.28 | **870.07** |
| 2 | **718.52** | 1416.97 |
| 3 | 825.46 | 1460.082 |
| 4 | 742.60 | 893.656 |

**Table S27.** Comparison of the relative fit (AICc) of three methods for estimating the initial probabilities of each state at the root of the animal tree (Tree I), using the corHMM approach. Results are shown for the best-fitting corHMM model for each coding strategy from Table S26. The observed states are 0 (herbivory) and 1 (carnivory). Hidden states within these observed states are labeled as R1–R2. Note that two hidden states per observed state are supported for maxcar coding, and no hidden states are supported for maxherb. The AICc values for the best-fitting method are boldfaced for each coding strategy. Columns ∑(0) and ∑(1) summarize the proportional likelihoods for states 0 and 1 at the root, summed across the different hidden states within each observed state, with values boldfaced for the best-fitting method.

| Coding | Method | AICc | Root state | | | | |  |
| --- | --- | --- | --- | --- | --- | --- | --- | --- |
|  |  |  | (0,R1) | (1,R1) | (0,R2) | (1,R2) | ∑(0) | ∑(1) |
| Maxcar | Yang | 717.53 | 9.6E-05 | 2.0E-03 | 2.2E-01 | 7.8E-01 | 0.218 | 0.782 |
|  | NULL | 733.03 | 6.7E-09 | 1.3E-06 | 1.0E+00 | 3.8E-06 | 1.000 | 0.000 |
|  | **Maddfitz** | **717.35** | 1.4E-06 | 4.6E-04 | 4.8E-01 | 5.2E-01 | **0.478** | **0.522** |
| Maxherb | Yang | 871.30 | 2.9E-1 | 0.70 | - | - | 2.9E-1 | 0.70 |
|  | NULL | 870.11 | 4.2E-2 | 0.95 | - | - | 4.2E-2 | 0.95 |
|  | **Maddfitz** | **868.89** | 7.4E-5 | 0.99 | - | - | **7.4E-5** | **0.99** |

**Table S28.** Estimated ancestral states for diet for key nodes of the animal Tree of Life (Tree I) based on the best fitting corHMM models (see Tables S26–S27). Estimates for all nodes are shown graphically in Figures S2 and S3.

| Coding | Clade | Herbivore | Carnivore |
| --- | --- | --- | --- |
| Maxcar | Root | 0.478 | 0.522 |
|  | Bilateria | 0.169 | 0.831 |
|  | Protostomia | 0.229 | 0.771 |
|  | Deuterostomia | 0.163 | 0.837 |
|  | Arthropoda | 0.043 | 0.957 |
|  | Chordata | 0.228 | 0.772 |
|  | Mollusca | 0.166 | 0.834 |
|  |  |  |  |
| Maxherb | Root | 0.017 | 0.983 |
|  | Bilateria | 0.001 | 0.999 |
|  | Protostomia | 0.001 | 0.999 |
|  | Deuterostomia | 0.001 | 0.999 |
|  | Arthropoda | 0.064 | 0.936 |
|  | Chordata | 0.001 | 0.999 |
|  | Mollusca | 0.001 | 0.999 |

**Table S29.** Comparison of the fit (AICc) of different corHMM models for the evolution of diet for Tree II. Models differ based on the number of hidden states within each observed state, from 0 to 4. The total number of hidden states is always twice the number of observed states because the number of hidden states per observed state must be equal for the two observed states (e.g., when there are two hidden states for carnivory, there must also be two hidden states for herbivory). Different hidden states differ based on their transition rates only, not differences in rates of speciation and extinction. Results are shown for the two coding strategies (maxcar and maxherb). The best-fitting model is boldfaced for each coding strategy. Transition rates correspond to the all-rates-different model (all transition between states are allowed to take a different rate, rather than being constrained to be equal).

| Hidden states per observed state | Maxcar | Maxherb |
| --- | --- | --- |
| 0 | 822.4520 | 871.1219 |
| 2 | 753.6300 | 869.9160 |
| 3 | **730.5047** | **706.9345** |
| 4 | 747.5435 | 779.8283 |

**Table S30.** Comparison of the relative fit (AICc) of three methods for estimating the initial probabilities of each state at the root of the animal tree, using the corHMM approach for Tree II. Results are shown for the best-fitting corHMM model for each coding strategy from Table S29. The observed states are 0 (herbivory) and 1 (carnivory). Hidden states within these observed states are labeled as R1–R3. Three hidden states per observed state are supported for both maxcar and maxherb coding. The AICc values for the best-fitting method are boldfaced for each coding strategy. Columns ∑(0) and ∑(1) summarize the proportional likelihoods for states 0 and 1 at the root, summed across the different hidden states within each observed state, with values boldfaced for the best-fitting method.

| Coding | Method | AICc | Root state | | | | | | | |
| --- | --- | --- | --- | --- | --- | --- | --- | --- | --- | --- |
|  |  |  | (0,R1) | (1,R1) | (0,R2) | (1,R2) | (0,R3) | (1,R3) | ∑(0) | ∑(1) |
| Maxcar | Yang | 725.3405 | 1.2E-10 | 1.6E-11 | 1.4E-09 | 3.7E-01 | 6.3E-01 | 1.2E-10 | 0.366 | 0.634 |
|  | NULL | 726.7932 | 7.3E-01 | 4.2E-03 | 2.6E-01 | 8.2E-04 | 4.4E-04 | 7.3E-01 | 0.005 | 0.995 |
|  | Maddfitz | **723.2973** | 9.6E-01 | 1.1E-07 | 4.0E-02 | 7.8E-10 | 1.3E-10 | 9.6E-01 | **0.000** | **1.000** |
|  |  |  |  |  |  |  |  |  |  |  |
| Maxherb | Yang | 773.942 | 9E-10 | 2.2E-3 | 6E-9 | 1 | 8.5E-15 | 1.6E-14 | 0.000 | 1.000 |
|  | NULL | **706.111** | 1.5E-2 | 9.5E-1 | 2.5E-2 | 1.5E-2 | 4.4E-9 | 2.7E-9 | **0.040** | **0.960** |
|  | Maddfitz | 767.398 | 1.3E-7 | 2.8E-22 | 1.9E-4 | 1.4E-2 | 4.4E-1 | 5.4E-1 | 0.441 | 0.559 |

**Table S31.** Estimated diet for key nodes of the animal Tree of Life based on the best-fitting corHMM model for Tree II (Tables S29–S30). Results are shown for both maxcar and maxherb coding. Values are proportional likelihoods of each observed, summed across the hidden states inferred for each observed state. Ancestral-state reconstructions used the best-fitting option for estimating the initial probabilities at the root for each state (maddfitz method for maxcar, and the default for maxherb).

| Coding | Clade | Herbivore | Carnivore |
| --- | --- | --- | --- |
| Maxcar | Root | 0.000 | 1.000 |
|  | Bilateria | 0.001 | 0.999 |
|  | Protostomia | 0.013 | 0.987 |
|  | Deuterostomia | 0.003 | 0.997 |
|  | Arthropoda | 0.005 | 0.995 |
|  | Chordata | 0.000 | 1.000 |
|  | Mollusca | 0.000 | 1.000 |
|  |  |  |  |
| Maxherb | Root | 0.040 | 0.960 |
|  | Bilateria | 0.153 | 0.847 |
|  | Protostomia | 0.082 | 0.918 |
|  | Deuterostomia | 0.113 | 0.887 |
|  | Arthropoda | 0.021 | 0.979 |
|  | Chordata | 0.004 | 0.996 |
|  | Mollusca | 0.000 | 1.000 |

**Table S32.** Comparison of the fit of different corHMM models for the evolution of diet for Tree III. Models differ based on the number of hidden states (rate categories) within each observed state, from 0 to 4. The total number of hidden states is always twice the number of observed states because the number of hidden states per observed state must be equal for the two observed states (e.g., when there are two hidden states for carnivory, there must also be two hidden states for herbivory). Different hidden states differ based on their transition rates only, not differences in rates of speciation and extinction. Results are shown for the two coding strategies (maxcar and maxherb). The best-fitting model is boldfaced for each coding strategy. Transition rates correspond to the all-rates-different model (all transition between states are allowed to take a different rate, rather than being constrained to be equal).

| Hidden states per observed state | Maxcar | Maxherb |
| --- | --- | --- |
| 0 | 828.6954 | 870.1156 |
| 2 | 1387.832 | 868.8769 |
| 3 | **728.2734** | **720.4860** |
| 4 | 748.5404 | 893.4632 |

**Table S33.** Comparison of the fit of three methods for estimating the initial probabilities of each state at the root of the animal tree, using the corHMM approach for Tree III. Methods are compared based on the AICc. Results are shown for the best-fitting corHMM model for each coding strategy from Table S32. The observed states are 0 (herbivory) and 1 (carnivory). Hidden states within these observed states are labeled as R1–R3. Three hidden states per observed state are supported for both maxcar and maxherb coding (Table S32). The AICc values for the best-fitting method are boldfaced for each coding strategy. Columns ∑(0) and ∑(1) summarize the proportional likelihoods for states 0 and 1 at the root, summed across the different hidden states within each observed state, with values boldfaced for the best-fitting method.

| Coding | Method | AICc | Root state | | | | | | | |
| --- | --- | --- | --- | --- | --- | --- | --- | --- | --- | --- |
|  |  |  | (0,R1) | (1,R1) | (0,R2) | (1,R2) | (0,R3) | (1,R3) | ∑(0) | ∑(1) |
| Maxcar | Yang | 728.6906 | 3.78E-23 | 1.77E-10 | 5.17E-11 | 1.99E-09 | 3.53E-01 | 6.47E-01 | 0.353 | 0.647 |
|  | NULL | 728.7669 | 7.59E-13 | 7.01E-01 | 6.51E-03 | 2.88E-01 | 2.31E-03 | 1.76E-03 | 0.009 | 0.991 |
|  | Maddfitz | **726.2839** | 1.17E-36 | 9.40E-01 | 5.31E-07 | 6.04E-02 | 2.44E-08 | 1.06E-08 | **0.000** | **1.000** |
|  |  |  |  |  |  |  |  |  |  |  |
| Maxherb | Yang | 724.0499 | 5.97E-24 | 1.17E-10 | 1.64E-11 | 1.35E-09 | 3.66E-01 | 6.34E-01 | 0.366 | 0.634 |
|  | NULL | 725.0794 | 3.18E-13 | 7.31E-01 | 4.20E-03 | 2.64E-01 | 8.16E-04 | 4.37E-04 | 0.005 | 0.995 |
|  | Maddfitz | **722.4853** | 7.73E-38 | 9.60E-01 | 1.08E-07 | 4.00E-02 | 7.78E-10 | 1.30E-10 | **0.000** | **1.000** |

**Table S34.** Estimated diet for key nodes of the animal Tree of Life based on the best-fitting corHMM model for Tree III (Tables S32–S33). Values are proportional likelihoods of each observed, summed across the hidden states inferred for each observed state. Ancestral-state reconstructions used the best-fitting option for estimating the initial probabilities at the root for each state (maddfitz for both maxcar and maxherb).

| Coding | Clade | Herbivore | Carnivore |
| --- | --- | --- | --- |
| Maxcar | Root | 0.000 | 1.000 |
|  | Bilateria | 0.000 | 1.000 |
|  | Deuterostomia | 0.001 | 0.999 |
|  | Arthropoda | 0.005 | 0.995 |
|  | Chordata | 0.000 | 1.000 |
|  | Mollusca | 0.002 | 0.998 |
|  |  |  |  |
| Maxherb | Root | 0.000 | 1.000 |
|  | Bilateria | 0.001 | 0.999 |
|  | Deuterostomia | 0.003 | 0.997 |
|  | Arthropoda | 0.005 | 0.995 |
|  | Chordata | 0.000 | 1.000 |
|  | Mollusca | 0.000 | 1.000 |

**Table S35.** Summary statistics for the models compared for ancestral-state reconstruction of diet with BayesTraits for Tree I. We compared 9 different models to find the best-fitting model(s) for each coding strategy. These models involved different combinations of prior distributions (exponential, uniform), sampling methods (Markov-Chain Monte Carlo, MCMC, with and without a reversible jump; rj-MCMC), and a parameter allowing variable rates across the tree (Venditti et al. 2011). For the analysis of each model, we ran three independent MCMC or rj-MCMC chains for 11 million generations (sampling every 1,000 generations), discarding the first 10% as burn-in. For each chain, mixing was assessed based on acceptance-rate values, with values from 20–40% considered indicative of optimal mixing (Venditti et al. 2011). Convergence was assessed based on the combined posterior distribution of the three chains by inspecting the harmonic mean over time and effective sample sizes (ESS, with ESS>200 indicating convergence (Pagel et al. 2004). Models were compared based on AIC values, after estimating the number of parameters based on the number of analyzed states (nstates), with the number of parameters equal to nstates^2^-nstates (following Venditti et al. 2011). For models including variable rates, each rate was treated as an independent parameter (Venditti et al. 2011). The posterior probability for each state for six key nodes in the animal tree (see above) was estimated for the best-fitting model for each coding strategy (and those with ∆AIC<4 from the best model; see Table S36). Both uniform and exponential priors were set on the transition rates between states (carnivore and herbivore) across the phylogeny. Four models with variable rates for the analyzed states were also fitted (Venditti et al. 2011). Log-likelihood values are summarized under the “Ln” column. For models that did not include variable rates, the number of parameters was estimated as nstates^2^-nstates (Venditti et al. 2011). The effective sample size (ESS) of the MCMC or rj-MCMC runs was estimated based on three independent runs. Acceptance rates (%) are indicated for each chain. The latter statistics suggest adequate mixing when values are between 20% and 40%. The best-fitting models for each strategy (∆AIC<4 from the best model) are highlighted in gray.

| Model | Dataset | Variable rates | Sampling | Prior | ESS | Ln | nstates | AIC | ∆AIC | Acceptance |
| --- | --- | --- | --- | --- | --- | --- | --- | --- | --- | --- |
| 1 | Maxcar | No | MCMC | Uniform (0,100) | 899 | -412.24 | 2 | 828.48 | 0.80 | 22.3–35.4% |
| 2 | Maxcar | No | MCMC | Uniform (0,10) | 8632 | -412.25 | 2 | 828.50 | 0.80 | 30.4–30.8% |
| 3 | Maxcar | No | MCMC | Exponential (10) | 8900 | -412.24 | 2 | 828.48 | 0.80 | 25.2–25.9% |
| 4 | Maxcar | Yes | MCMC | Exponential (10) | 276 | -327.18 | 140+2 | 938.36 | 110.68 | 14.8–50.9% |
| 5 | Maxcar | Yes | MCMC | Uniform (0,100) | 364 | -328.63 | 140+2 | 943.26 | 115.58 | 10.6–52.6% |
| 6 | Maxcar | No | rj-MCMC | Exponential (10) | 7888 | -411.85 | 2 | 827.70 | 0.02 | 18.7–40.6% |
| 7 | Maxcar | No | rj-MCMC | Uniform (0,100) | 10224 | -411.84 | 2 | 827.68 | 0.00 | 21.9–35.0% |
| 8 | Maxcar | Yes | rj-MCMC | Exponential (10) | 276 | -327.18 | 139+2 | 936.36 | 108.68 | 10.6–52.6% |
| 9 | Maxcar | Yes | rj-MCMC | Uniform (0,100) | 486 | -325.66 | 138+2 | 931.32 | 103.64 | 13.1–50.0% |
| 1 | Maxherb | No | MCMC | Exponential (10) | 8484 | -433.20 | 2 | 870.40 | 0.00 | 26.2–40.4% |
| 2 | Maxherb | No | MCMC | Uniform (0,100) | 9000 | -441.07 | 2 | 886.14 | 15.74 | 21.6–33.5% |
| 3 | Maxherb | Yes | MCMC | Exponential (10) | 830 | -357.34 | 140+2 | 998.68 | 128.28 | 14–27.59% |
| 4 | Maxherb | Yes | MCMC | Uniform (0,100) | 716 | -358.25 | 140+2 | 1000.50 | 130.10 | 10.6–35% |
| 5 | Maxherb | No | rj-MCMC | Exponential (10) | 9345 | -441.08 | 2 | 886.16 | 15.76 | 24.5–37.8% |
| 6 | Maxherb | No | rj-MCMC | Uniform (0,100) | 9000 | -443.20 | 2 | 890.40 | 20.00 | 18.6–42.2% |
| 7 | Maxherb | Yes | rj-MCMC | Exponential (10) | 507 | -356.82 | 139+2 | 995.64 | 125.24 | 11.3–60.8% |
| 8 | Maxherb | Yes | rj-MCMC | Uniform (0,100) | 640 | -355.27 | 138+2 | 992.54 | 122.14 | 12.6–56.2% |

**Table S36.** Estimated ancestral states for diet for key nodes of the animal Tree of Life (Tree I) based on BayesTraits. Results are based on the best-fitting models for each coding strategy (see Table S35). Values are the posterior probabilities for each state at each node (C=carnivore, H=herbivore). Estimates for all nodes for all these models are shown in Figures S4–S9.

|  | Maxcar | | | | | | | | | | Maxherb | |
| --- | --- | --- | --- | --- | --- | --- | --- | --- | --- | --- | --- | --- |
|  | Model 1 | | Model 2 | | Model 3 | | Model 6 | | Model 7 | | Model 1 | |
|  | C | H | C | H | C | H | C | H | C | H | C | H |
| Root | 0.9831 | 0.0169 | 0.9832 | 0.0168 | 0.9833 | 0.0167 | 0.9798 | 0.0202 | 0.9798 | 0.0202 | 0.8428 | 0.1571 |
| Bilateria | 0.9947 | 0.0053 | 0.9947 | 0.0053 | 0.9948 | 0.0052 | 0.9933 | 0.0067 | 0.9933 | 0.0067 | 0.7233 | 0.2766 |
| Protostomia | 0.9952 | 0.0048 | 0.9952 | 0.0048 | 0.9953 | 0.0047 | 0.9940 | 0.0060 | 0.9940 | 0.0060 | 0.9990 | 0.0009 |
| Deuterostomia | 0.9945 | 0.0055 | 0.9944 | 0.0056 | 0.9946 | 0.0054 | 0.9867 | 0.0133 | 0.9867 | 0.0133 | 0.9968 | 0.0031 |
| Arthropoda | 0.9985 | 0.0015 | 0.9985 | 0.0015 | 0.9985 | 0.0015 | 0.9978 | 0.0022 | 0.9978 | 0.0022 | 0.9970 | 0.0029 |
| Chordata | 0.9917 | 0.0083 | 0.9917 | 0.0083 | 0.9918 | 0.0082 | 0.9877 | 0.0123 | 0.9877 | 0.0123 | 0.9021 | 0.0978 |
| Mollusca | 0.9674 | 0.0326 | 0.9677 | 0.0323 | 0.9680 | 0.0320 | 0.9587 | 0.0413 | 0.9587 | 0.0413 | 0.4162 | 0.5837 |

**Table S37.** Comparison of the fit of different root states for animal phylogeny (Tree I) using BayesTraits. For the best-fitting models identified in Table S35, the fit of different root states for animal phylogeny were compared for each coding strategy (maxcar and maxherb) using the AIC. We compared the fit of an unconstrained model (see Tables S35–S36), a model constraining the ancestor of all animals to be carnivorous (posterior probability at the root=1), or herbivorous. The best-fitting root-state assumptions (AIC<4) are highlighted in gray.

| Strategy | Model | Root state | Likelihood | Parameters | AIC |
| --- | --- | --- | --- | --- | --- |
| Maxcar | Model 1 | Unconstrained | -412.24 | 2 | 828.48 |
|  |  | Root: carnivore | -413.30 | 2 | 830.60 |
|  |  | Root: herbivore | -450.21 | 2 | 904.42 |
|  | Model 2 | Unconstrained | -412.25 | 2 | 828.50 |
|  |  | Root: carnivore | -414.76 | 2 | 833.52 |
|  |  | Root: herbivore | -467.21 | 2 | 938.42 |
|  | Model 3 | Unconstrained | -412.24 | 2 | 828.48 |
|  |  | Root: carnivore | -412.27 | 2 | 828.54 |
|  |  | Root: herbivore | -458.87 | 2 | 921.74 |
|  | Model 6 | Unconstrained | -411.84 | 2 | 827.68 |
|  |  | Root: carnivore | -411.65 | 2 | 827.30 |
|  |  | Root: herbivore | -420.54 | 2 | 845.08 |
|  | Model 7 | Unconstrained | -411.85 | 2 | 827.70 |
|  |  | Root: carnivore | -411.76 | 2 | 827.52 |
|  |  | Root: herbivore | -432.84 | 2 | 869.68 |
| Maxherb | Model 1 | Unconstrained | -433.20 | 2 | 870.40 |
|  |  | Root: carnivore | -432.54 | 2 | 869.08 |
|  |  | Root: herbivore | -454.89 | 2 | 913.78 |

**Table S38.** Summary statistics for the 8 models compared for ancestral-state reconstruction of diet with BayesTraits for Tree II. We compared 8 different models to find the best-fitting model(s) for each coding strategy (note that we here excluded one of two very similar models that were compared in Table S35 for Tree I). These models involved different combinations of prior distributions (exponential, uniform), sampling methods (Markov-Chain Monte Carlo, MCMC, with and without a reversible jump; rj-MCMC), and a parameter allowing variable rates across the tree (Venditti et al. 2011). For the analysis of each model, we ran three independent MCMC or rj-MCMC chains for 11 million generations (sampling every 1,000 generations), discarding the first 10% as burn-in. For each chain, mixing was assessed based on acceptance-rate values, with values from 20–40% considered indicative of optimal mixing (Venditti et al. 2011). Convergence was assessed based on the combined posterior distribution of the three chains by inspecting the harmonic mean over time and effective sample sizes (ESS, with ESS>200 indicating convergence (Pagel et al. 2004). Models were compared based on AIC values, after estimating the number of parameters based on the number of analyzed states (nstates), with the number of parameters equal to nstates^2^-nstates (following Venditti et al. 2011). For models including variable rates, each rate was treated as an independent parameter (Venditti et al. 2011). The posterior probability for each state for six key nodes in the animal tree (see above) was estimated for the best-fitting model for each coding strategy (and those with ∆AIC<4 from the best model; Table S39). Both uniform and exponential priors were set on the transition rates between states (carnivore and herbivore) across the phylogeny. Four models with variable rates for the analyzed states were also fitted (Venditti et al. 2011). Log-likelihood values are summarized under the “Ln” column. For models that did not include variable rates, the number of parameters was estimated as nstates^2^-nstates (Venditti et al. 2011). The effective sample size (ESS) of the MCMC or rj-MCMC runs was estimated based on three independent runs. Acceptance rates (%) are indicated for each chain. The latter statistics suggest adequate mixing when values are between 20% and 40%. The best-fitting models for each strategy (∆AIC<4 from the best model) are highlighted in gray.

| Model | Coding strategy | Variable rates | Sampling | Prior | ESS | Ln | nstates | AIC | dAIC | Acceptance (%) |
| --- | --- | --- | --- | --- | --- | --- | --- | --- | --- | --- |
| 1 | Maxcar | No | MCMC | Exponential (10) | 10000.00 | -411.76 | 2.00 | 827.52 | 1.03 | 25–26 |
| 2 | Maxcar | No | MCMC | Uniform (0,100) | 10000.00 | -411.77 | 2.00 | 827.54 | 1.05 | 26–28 |
| 3 | Maxcar | Yes | MCMC | Exponential (10) | 774.43 | -321.15 | 142.00 | 926.31 | 99.82 | 23–24 |
| 4 | Maxcar | Yes | MCMC | Uniform (0,100) | 682.76 | -318.60 | 144.00 | 925.19 | 98.71 | 23–24 |
| 5 | Maxcar | No | rj-MCMC | Exponential (10) | 5000.00 | -411.24 | 2.00 | 826.49 | 0.00 | 25–25 |
| 6 | Maxcar | No | rj-MCMC | Uniform (0,100) | 5000.00 | -411.24 | 2.00 | 826.49 | 0.00 | 24–24 |
| 7 | Maxcar | Yes | rj-MCMC | Exponential (10) | 542.54 | -319.55 | 140.00 | 919.09 | 92.60 | 22 |
| 8 | Maxcar | Yes | rj-MCMC | Uniform (0,100) | 398.07 | -319.56 | 141.00 | 921.13 | 94.64 | 22–23 |
| 1 | Maxherb | No | MCMC | Exponential (10) | 9000 | -310.58 | 2.00 | 625.16 | 0.00 | 22.8–36.5 |
| 2 | Maxherb | No | MCMC | Uniform (0,100) | 9467.99 | -440.59 | 2.00 | 885.18 | 260.02 | 23.9–36 |
| 3 | Maxherb | Yes | MCMC | Exponential (10) | 711.20 | -357.12 | 142.00 | 998.24 | 373.08 | 12.9–58.2 |
| 4 | Maxherb | Yes | MCMC | Uniform (0,100) | 858.39 | -357.39 | 144.00 | 1002.78 | 377.62 | 26.4–56 |
| 5 | Maxherb | No | rj-MCMC | Exponential (10) | 627.12 | -353.12 | 2.00 | 710.24 | 85.08 | 24.3–32.5 |
| 6 | Maxherb | No | rj-MCMC | Uniform (0,100) | 9000 | -442.58 | 2.00 | 889.16 | 264.00 | 21.5–35.6 |
| 7 | Maxherb | Yes | rj-MCMC | Exponential (10) | 547.19 | -355.36 | 140.00 | 990.72 | 365.56 | 29.1–65.4 |
| 8 | Maxherb | Yes | rj-MCMC | Uniform (0,100) | 627.12 | -353.87 | 141.00 | 989.74 | 364.58 | 21.5–60.4 |

**Table S39.** Estimated diet for key nodes of the animal Tree of Life (Tree II) based on the best fitting BayesTraits models (Table S38). Values are posterior probabilities of each observed state. Results are shown for each coding strategy (maxcar and maxherb).

| Clade | Maxcar | | | | | | | | Maxherb | |
| --- | --- | --- | --- | --- | --- | --- | --- | --- | --- | --- |
|  | Model 1 | | Model 2 | | Model 5 | | Model 6 | | Model 1 | |
|  | C | H | C | H | C | H | C | H | C | H |
| Root | 0.959 | 0.041 | 0.959 | 0.041 | 0.963 | 0.037 | 0.963 | 0.037 | 0.878 | 0.122 |
| Bilateria | 0.992 | 0.008 | 0.992 | 0.008 | 0.993 | 0.007 | 0.993 | 0.007 | 0.768 | 0.231 |
| Protostomia | 0.988 | 0.012 | 0.988 | 0.012 | 0.990 | 0.010 | 0.990 | 0.010 | 0.769 | 0.231 |
| Deuterostomia | 0.995 | 0.005 | 0.995 | 0.005 | 0.997 | 0.003 | 0.997 | 0.003 | 0.583 | 0.416 |
| Arthropoda | 0.994 | 0.006 | 0.994 | 0.006 | 0.995 | 0.005 | 0.995 | 0.005 | 0.513 | 0.486 |
| Chordata | 0.923 | 0.077 | 0.923 | 0.077 | 0.934 | 0.066 | 0.934 | 0.066 | 0.803 | 0.197 |
| Mollusca | 0.859 | 0.141 | 0.860 | 0.140 | 0.898 | 0.102 | 0.898 | 0.102 | 0.403 | 0.596 |

**Table S40.** Summary statistics for the 8 models compared for ancestral-state reconstruction of diet with BayesTraits for Tree III. We compared 8 different models to find the best-fitting model(s) for each coding strategy (note that we here excluded one of two very similar models that were compared in Table S35 for Tree I). These models involved different combinations of prior distributions (exponential, uniform), sampling methods (Markov-Chain Monte Carlo, MCMC, with and without a reversible jump; rj-MCMC), and a parameter allowing variable rates across the tree (Venditti et al. 2011). For the analysis of each model, we ran three independent MCMC or rj-MCMC chains for 11 million generations (sampling every 1,000 generations), discarding the first 10% as burn-in. For each chain, mixing was assessed based on acceptance-rate values, with values from 20–40% considered indicative of optimal mixing (Venditti et al. 2011). Convergence was assessed based on the combined posterior distribution of the three chains by inspecting the harmonic mean over time and effective sample sizes (ESS, with ESS>200 indicating convergence; Pagel et al. 2004). Models were compared based on AIC values, after estimating the number of parameters based on the number of analyzed states (nstates), with the number of parameters equal to nstates^2^-nstates (following Venditti et al. 2011). For models including variable rates, each rate was treated as an independent parameter (Venditti et al. 2011). The posterior probability for each state for six key nodes in the animal tree (see above) was estimated for the best-fitting model for each coding strategy (and those with ∆AIC<4 from the best model; Table S41). Both uniform and exponential priors were set on the transition rates between states (carnivore and herbivore) across the phylogeny. Four models with variable rates for the analyzed states were also fitted (Venditti et al. 2011). Log-likelihood values are summarized under the “Ln” column. For models that did not include variable rates, the number of parameters was estimated as nstates^2^-nstates (Venditti et al. 2011). The effective sample size (ESS) of the MCMC or rj-MCMC runs was estimated based on three independent runs. Acceptance rates (%) are indicated for each chain. The latter statistics suggest adequate mixing when values are between 20% and 40%. The best-fitting models for each strategy (∆AIC<4 from the best model) are highlighted in gray.

| Model | Dataset | Variable rates | Sampling | Prior | ESS | Ln | nstates | AIC | ∆AIC | Acceptance (%) |
| --- | --- | --- | --- | --- | --- | --- | --- | --- | --- | --- |
| 1 | Maxcar | No | MCMC | Exponential (10) | 10000.00 | -412.70 | 2.00 | 829.39 | 1.12 | 30–30 |
| 2 | Maxcar | No | MCMC | Uniform (0,100) | 10000.00 | -412.67 | 2.00 | 829.33 | 1.06 | 30–35 |
| 3 | Maxcar | Yes | MCMC | Exponential (10) | 605.16 | -322.80 | 142.00 | 929.59 | 101.32 | 23–24 |
| 4 | Maxcar | Yes | MCMC | Uniform (0,100) | 722.99 | -319.85 | 141.00 | 921.69 | 93.42 | 23–24 |
| 5 | Maxcar | No | rj-MCMC | Exponential (10) | 5000.00 | -412.14 | 2.00 | 828.27 | 0.00 | 22–24 |
| 6 | Maxcar | No | rj-MCMC | Uniform (0,100) | 5000.00 | -412.17 | 2.00 | 828.33 | 0.06 | 22–22 |
| 7 | Maxcar | Yes | rj-MCMC | Exponential (10) | 648.70 | -321.26 | 140.00 | 922.53 | 94.26 | 22–22 |
| 8 | Maxcar | Yes | rj-MCMC | Uniform (0,100) | 560.90 | -318.97 | 142.00 | 921.94 | 93.67 | 22–22 |
| 1 | Maxherb | No | MCMC | Exponential (10) | 9000 | -441.27 | 2.00 | 886.54 | 16.40 | 20.9–33 |
| 2 | Maxherb | No | MCMC | Uniform (0,100) | 8085.28 | -433.07 | 2 | 870.14 | 0 | 23.1–36.3 |
| 3 | Maxherb | Yes | MCMC | Exponential (10) | 741.37 | -357.39 | 142.00 | 1038.78 | 168.64 | 11.5–52.3 |
| 4 | Maxherb | Yes | MCMC | Uniform (0,100) | 642.50 | -357.55 | 141.00 | 997.10 | 126.96 | 18.1–46.9 |
| 5 | Maxherb | No | rj-MCMC | Exponential (10) | 9000 | -441.25 | 2 | 986.5 | 116.36 | 22.3–34.1 |
| 6 | Maxherb | No | rj-MCMC | Uniform (0,100) | 9000 | -443.07 | 2.00 | 910.14 | 40.00 | 19.8–42.1 |
| 7 | Maxherb | Yes | rj-MCMC | Exponential (10) | 435.54 | -357.60 | 140.00 | 985.20 | 115.06 | 24.5–37.2 |
| 8 | Maxherb | Yes | rj-MCMC | Uniform (0,100) | 588.18 | -356.14 | 142.00 | 1002.28 | 132.14 | 25.9–45.2 |

**Table S41.** Estimated diet for key nodes of the animal Tree of Life based on the best fitting BayesTraits models for Tree III. Values are posterior probabilities of each observed state. Results are shown for each coding strategy (maxcar and maxherb). Model definitions are provided in Table S40.

|  | Maxcar | | | | | | | | Maxherb | |
| --- | --- | --- | --- | --- | --- | --- | --- | --- | --- | --- |
|  | Model 1 | | Model 2 | | Model 5 | | Model 6 | | Model 2 | |
|  | C | H | C | H | C | H | C | H | C | H |
| Root | 0.919 | 0.081 | 0.920 | 0.080 | 0.925 | 0.075 | 0.925 | 0.075 | 0.859 | 0.141 |
| Bilateria | 0.996 | 0.004 | 0.996 | 0.004 | 0.996 | 0.004 | 0.996 | 0.004 | 0.717 | 0.282 |
| Deuterostomia | 0.975 | 0.025 | 0.975 | 0.025 | 0.980 | 0.020 | 0.980 | 0.020 | 0.915 | 0.008 |
| Arthropoda | 0.950 | 0.050 | 0.951 | 0.049 | 0.958 | 0.042 | 0.958 | 0.042 | 0.997 | 0.003 |
| Chordata | 0.958 | 0.042 | 0.958 | 0.042 | 0.965 | 0.035 | 0.965 | 0.035 | 0.900 | 0.100 |
| Mollusca | 0.891 | 0.109 | 0.893 | 0.107 | 0.928 | 0.072 | 0.928 | 0.072 | 0.415 | 0.584 |

**Table S42.** Transition-rate matrices for the best-fitting HiSSE model for both coding strategies (M24 for maxcar and maxherb strategies; see Table S6). Observed states are 0 for herbivory and 1 for carnivory. Hidden states are labeled A and B. Thus, transitions from carnivory to herbivory are (1A)(1B) to (0A)(0B) and (1B)(1A) to (0B)(0A) and transitions from herbivory to carnivory are 0A to 1A and 0B to 1B. Transition rates should be read from “row” to “column” (i.e. the starting state is the row, the end state is the column, such that the first row shows transition rates from 0A to 1A, 0A to 0B, and 0A to 1B). Based on the rates estimated below, we estimated the overall transition rates between the two diet states by averaging rates associated with each of the hidden states (the overall transition rate for carnivory to herbivory is the average of the rates from 1A to 0A and 1B to 0B and the overall rate for transitions from herbivory to carnivory is the average of the rates from 0A to 1A and 0B to 1B). For maxcar coding the overall rate for transitions from carnivory to herbivory is 0.0005188 and for herbivory to carnivory the overall rate is 0.0001985, with transitions from carnivory to herbivory 2.61 times more common than transitions from herbivory to carnivory (ratio of the two rates). For maxherb coding, the overall rate for transitions from carnivory to herbivory is 0.0006257 and the rate for transitions from herbivory to carnivory is 0.0003179, with transitions from carnivory to herbivory 1.97 times more common than transitions from herbivory to carnivory.

| Strategy | States | 0A | 1A | 0B | 1B |
| --- | --- | --- | --- | --- | --- |
| Maxcar (M24) | 0A | - | 4.38E-04 | 1.12E-02 | 0.00 |
|  | 1A | 1.69E-03 | - | 0.00 | 1.23E-02 |
|  | 0B | 8.83E-04 | 0.00 | - | 3.56E-04 |
|  | 1B | 0.00 | 6.03E-04 | 3.85E-04 | - |
| Maxherb (M24) | 0A | - | 1.88E-04 | 7.84E-05 | 0.00 |
|  | 1A | 5.44E-04 | - | 0.00 | 2.06E-09 |
|  | 0B | 1.54E-02 | 0.00 | - | 1.08E-03 |
|  | 1B | 0.00 | 1.38E-02 | 1.95E-03 | - |

**Table S43.** Results of PGLS analyses testing the relationship between habitat and diet among animal phyla. Data on the proportion of non-marine species in each phylum and the proportion of herbivorous species were from Table S10. See Methods for other details of PGLS analyses. Tree I was used.

| *r^2^* | *P*-value |
| --- | --- |
| 0.002 | 0.8206 |

**Table S44.** Comparison of the fit of different models for the evolution of diet, and estimated level of phylogenetic signal (lambda) for Tree I, using an alternative dataset. Analyses were based on a matrix in which states for four phyla were modified to better reflect estimates of diet based on all species in each phylum (Dataset S3). The relative fit of two models was compared based on AICc values: a model with no phylogenetic signal (white noise model) and one with phylogenetic signal (lambda model). The best-fitting model is boldfaced. The estimated value of lambda quantifies the level of phylogenetic signal, from 0 to 1 (maximum signal). Significant lambda values (*P*<0.0001; tested by simulations) are asterisked. For these analyses, we initially included all three states (i.e. carnivorous, herbivorous, and omnivorous; the 2% of taxa with ambiguous or unknown states were excluded). Alternatively, all omnivorous and ambiguous taxa (5% of all sampled taxa) were coded as either carnivorous (maxcar) or herbivorous (maxherb).

| Dataset | Model | Ln-likelihood | AICc |
| --- | --- | --- | --- |
| Three states | White-noise | -1069.580 | 2143.172 |
|  | Lambda (λ=0.80*) | -844.292 | **1694.607** |
| Maxcar | White-noise | -715.270 | 1434.551 |
|  | Lambda (λ=0.84*) | -298.262 | **602.547** |
| Maxherb | White-noise | -750.787 | 1505.586 |
|  | Lambda (λ=0.85*) | -345.590 | **697.203** |

**Table S45.** Testing for phylogenetic signal in diet using the D-statistic for Tree I, using an alternative dataset. Analyses were based on a matrix in which states for four phyla were modified to better reflect estimates of diet based on all species in each phylum (Dataset S3). Estimated D is scaled based on D-values simulated under the Brownian motion model (strong phylogenetic signal) and random noise (no phylogenetic signal). Smaller values indicate stronger support for phylogenetic signal, with negative values showing that traits are highly conserved. Probabilities (*P*-values) indicate whether the observed D-statistic is significantly different from 0 (Brownian motion) and from 1 (random noise). Since the D-statistic is designed for binary data, two coding strategies were used, treating omnivorous and ambiguous taxa (5% of all sampled taxa) as either carnivorous (maxcar) or herbivorous (maxherb).

| Coding strategy | Estimated D | Probability of D different from Brownian motion (strong signal) | Probability of D different from random noise (no signal) |
| --- | --- | --- | --- |
| Maxcar | -0.497 | 0.999 | <0.0001 |
| Maxherb | -0.446 | 0.992 | <0.0001 |

**Table S46.** Comparison of the fit of different models for the evolution of diet, and estimated level of phylogenetic signal (lambda) for Tree II, using an alternative dataset. Analyses were based on a matrix in which states for four phyla were modified to better reflect estimates of diet based on all species in each phylum (Dataset S3). The relative fit of two models was compared based on AICc values: a model with no phylogenetic signal (white noise model) and one with phylogenetic signal (lambda model). The best-fitting model is boldfaced. The estimated value of lambda quantifies the level of phylogenetic signal, from 0 to 1 (maximum signal). Significant lambda values (*P*<0.0001; tested by simulations) are asterisked. For these analyses, we initially included all three states (i.e. carnivorous, herbivorous, and omnivorous; the 2% of taxa with ambiguous or unknown states were excluded). Alternatively, all omnivorous and ambiguous taxa (5% of all sampled taxa) were coded as either carnivorous (maxcar) or herbivorous (maxherb).

| Dataset | Model | Ln-likelihood | AICc |
| --- | --- | --- | --- |
| Three states | White-noise | -1059.794 | 2123.600 |
|  | Lambda (λ=0.82*) | -835.852 | **1677.727** |
| Maxcar | White-noise | -713.628 | 1431.267 |
|  | Lambda (λ=0.86*) | -298.268 | **602.558** |
| Maxherb | White-noise | -750.787 | 1505.586 |
|  | Lambda (λ=0.87*) | -346.178 | **698.378** |

**Table S47.** Testing for phylogenetic signal in diet using the D-statistic for Tree II, using an alternative dataset. Analyses were based on a matrix in which states for four phyla were modified to better reflect estimates of diet based on all species in each phylum (Dataset S3). Estimated D is scaled based on D-values simulated under the Brownian motion model (strong phylogenetic signal) and random noise (no phylogenetic signal). Smaller values indicate stronger support for phylogenetic signal, with negative values showing that traits are highly conserved. Probabilities (*P*-values) indicate whether the observed D-statistic is significantly different from 0 (Brownian motion) and from 1 (random noise). Since the D-statistic is designed for binary data, two coding strategies were used, treating omnivorous and ambiguous taxa (5% of all sampled taxa) as either carnivorous (maxcar) or herbivorous (maxherb).

| Coding strategy | Estimated D | Probability of D different from Brownian motion (strong signal) | Probability of D different from random noise (no signal) |
| --- | --- | --- | --- |
| Maxcar | -0.482 | 0.997 | <0.0001 |
| Maxherb | -0.443 | 0.996 | <0.0001 |

**Table S48.** Comparison of the fit of different models for the evolution of diet, and estimated level of phylogenetic signal (lambda) for Tree III, using an alternative dataset. Analyses were based on a matrix in which states for four phyla were modified to better reflect estimates of diet based on all species in each phylum (Dataset S3). The relative fit of two models was compared based on AICc values: a model with no phylogenetic signal (white noise model) and one with phylogenetic signal (lambda model). The best-fitting model is boldfaced. The estimated value of lambda quantifies the level of phylogenetic signal, from 0 to 1 (maximum signal). Significant lambda values (*P*<0.0001; tested by simulations) are asterisked. For these analyses, we initially included all three states (i.e. carnivorous, herbivorous, and omnivorous; the 2% of taxa with ambiguous or unknown states were excluded). Alternatively, all omnivorous and ambiguous taxa (5% of all sampled taxa) were coded as either carnivorous (maxcar) or herbivorous (maxherb).

| Dataset | Model | Ln-likelihood | AICc |
| --- | --- | --- | --- |
| Three states | White-noise | -1069.580 | 2143.172 |
|  | Lambda (λ=0.82*) | -843.483 | **1692.988** |
| Maxcar | White-noise | -715.270 | 1434.551 |
|  | Lambda (λ=0.86*) | -301.203 | **608.426** |
| Maxherb | White-noise | -750.787 | 1505.575 |
|  | Lambda (λ=0.85*) | -345.736 | **697.495** |

**Table S49.** Testing for phylogenetic signal in diet using the D-statistic for Tree III, using an alternative dataset. Analyses were based on a matrix in which states for four phyla were modified to better reflect estimates of diet based on all species in each phylum (Dataset S3). Estimated D is scaled based on D-values simulated under the Brownian motion model (strong phylogenetic signal) and random noise (no phylogenetic signal). Smaller values indicate stronger support for phylogenetic signal, with negative values showing that traits are highly conserved. Probabilities (*P*-values) indicate whether the observed D-statistic is significantly different from 0 (Brownian motion) and from 1 (random noise). Since the D-statistic is designed for binary data, two coding strategies were used, treating omnivorous and ambiguous taxa (5% of all sampled taxa) as either carnivorous (maxcar) or herbivorous (maxherb).

| Coding strategy | Estimated D | Probability of D different from Brownian motion (strong signal) | Probability of D different from random noise (no signal) |
| --- | --- | --- | --- |
| Maxcar | -0.471 | 0.996 | <0.0001 |
| Maxherb | -0.434 | 0.997 | <0.0001 |

**Figure S1.** Ancestral-state reconstruction for the best-fitting HiSSE model (M24) for maxherb coding and using the maddfitz method for estimating the state at the root (for Tree I). Colored pies at nodes indicate the proportional likelihoods of both observed and hidden states.

**
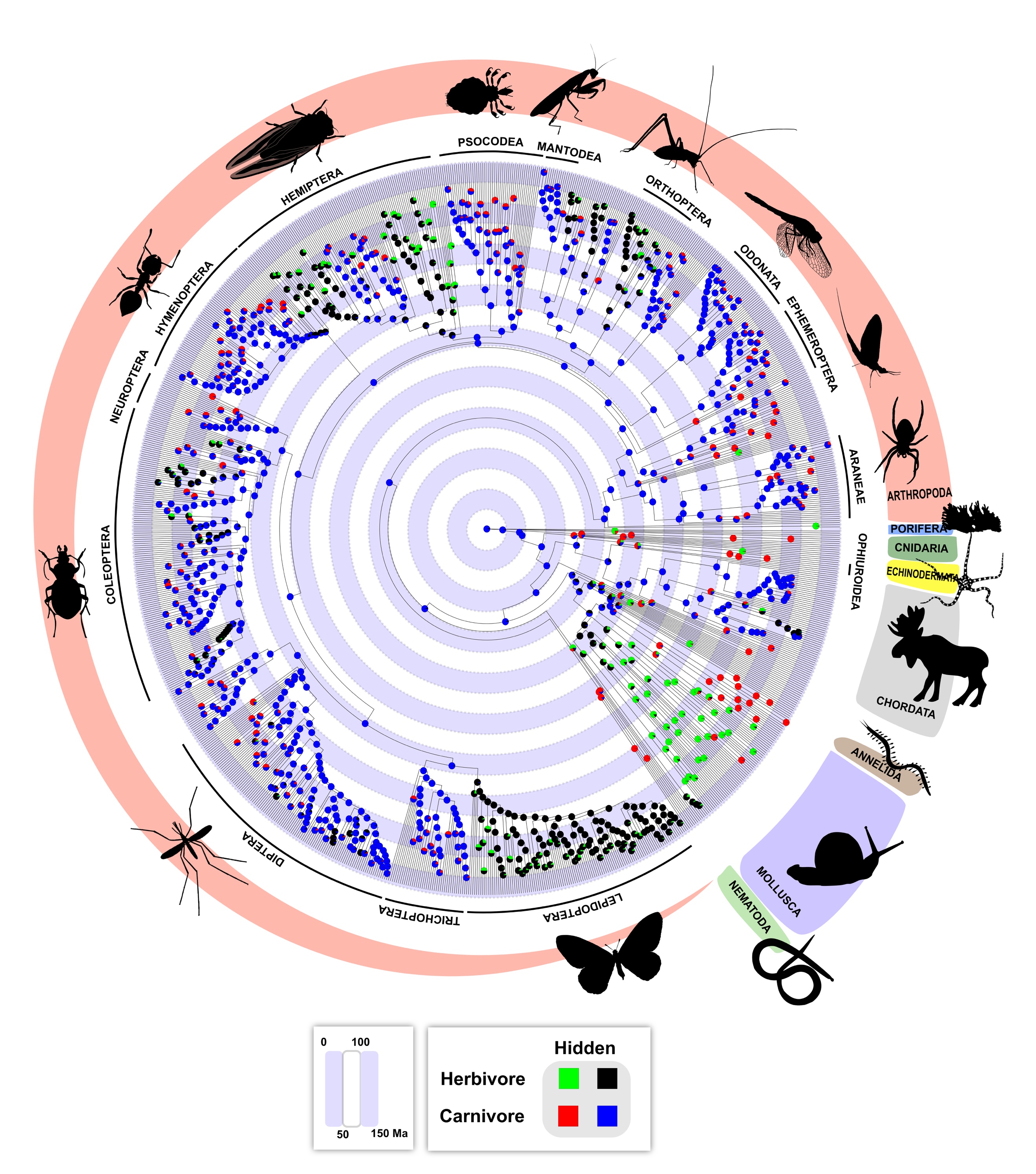
**

**Figure S2.** Ancestral state reconstructions for the best-fitting model using corHMM and the maxcar coding strategy (for Tree I), and using the maddfitz method for estimating the initial probabilities at the root for each state. Two hidden states are supported for each observed state. See Table S26–S28 for details.

**
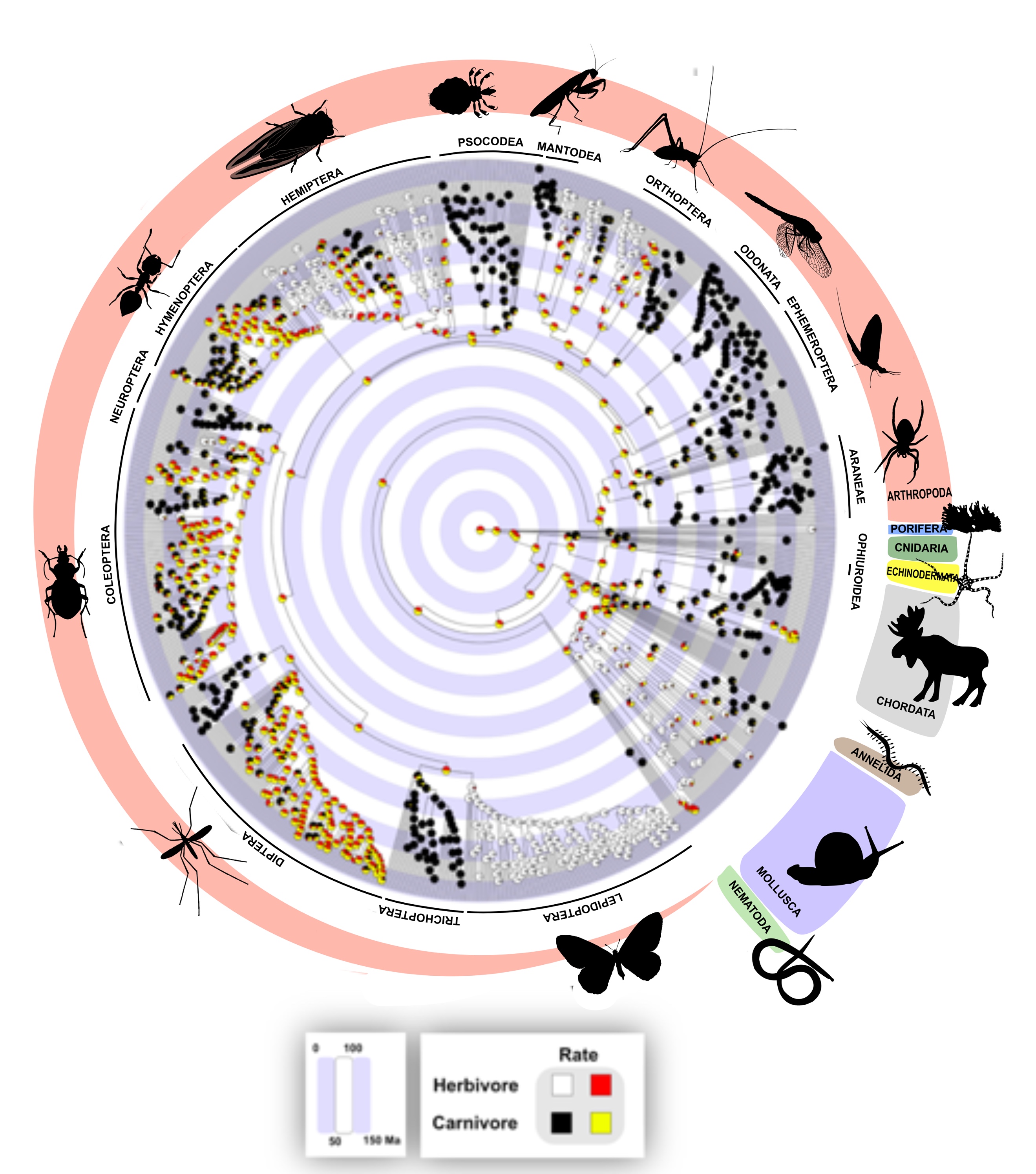
**

**Figure S3.** Ancestral state reconstructions for Tree I for the best-fitting model using corHMM and the maxherb coding strategy (treating omnivorous and ambiguous taxa as herbivorous), and using the maddfitz method for estimating the initial probabilities at the root for each state. No hidden states are supported for each observed state. See Table S26–S28 for details.


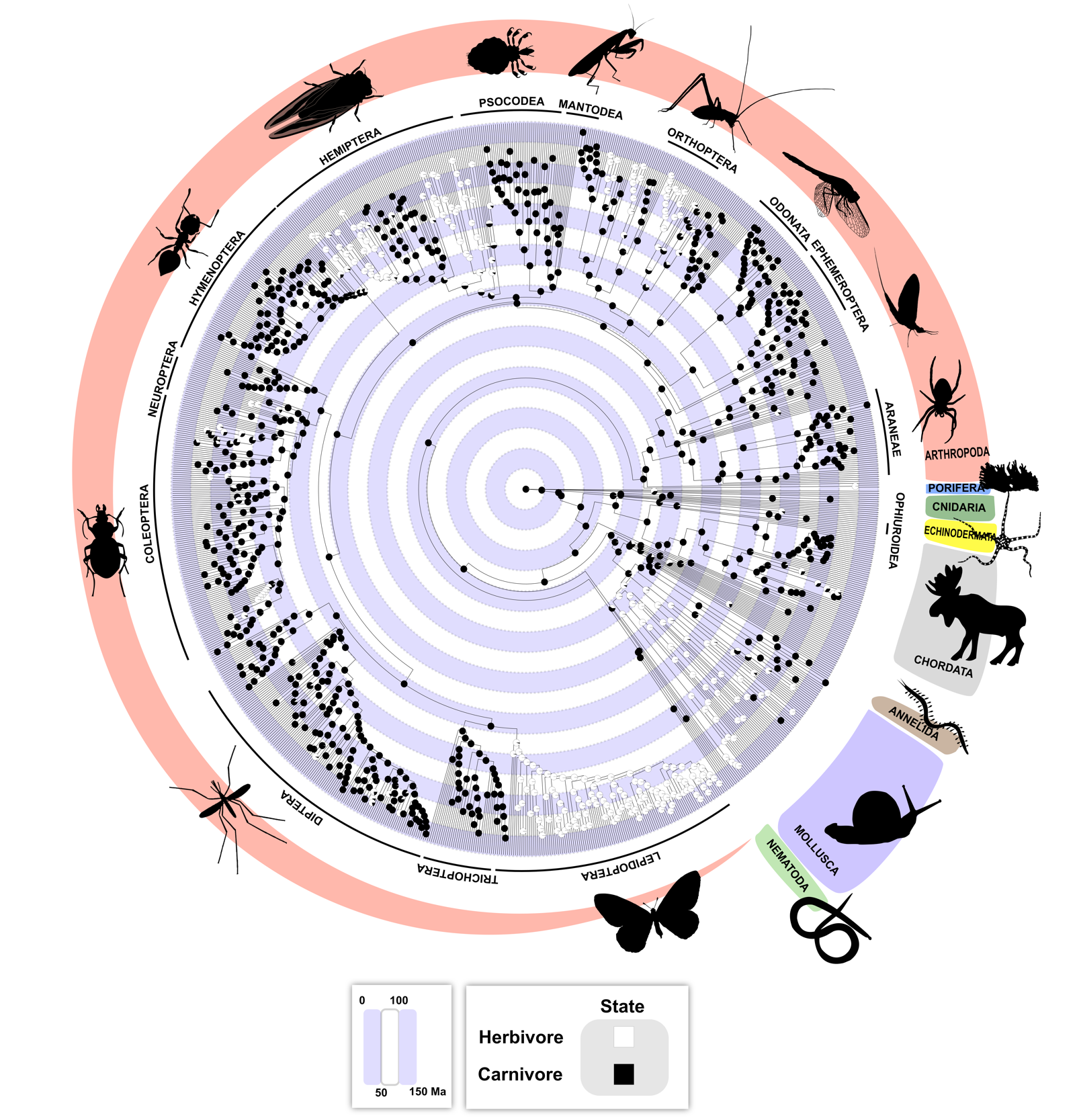


**Figure S4.** Ancestral-state reconstructions for Tree I for the M1 model fitted in BayesTraits using the maxherb coding strategy (treating omnivorous and ambiguous taxa as herbivorous). See Table S35–S36 for details.

**
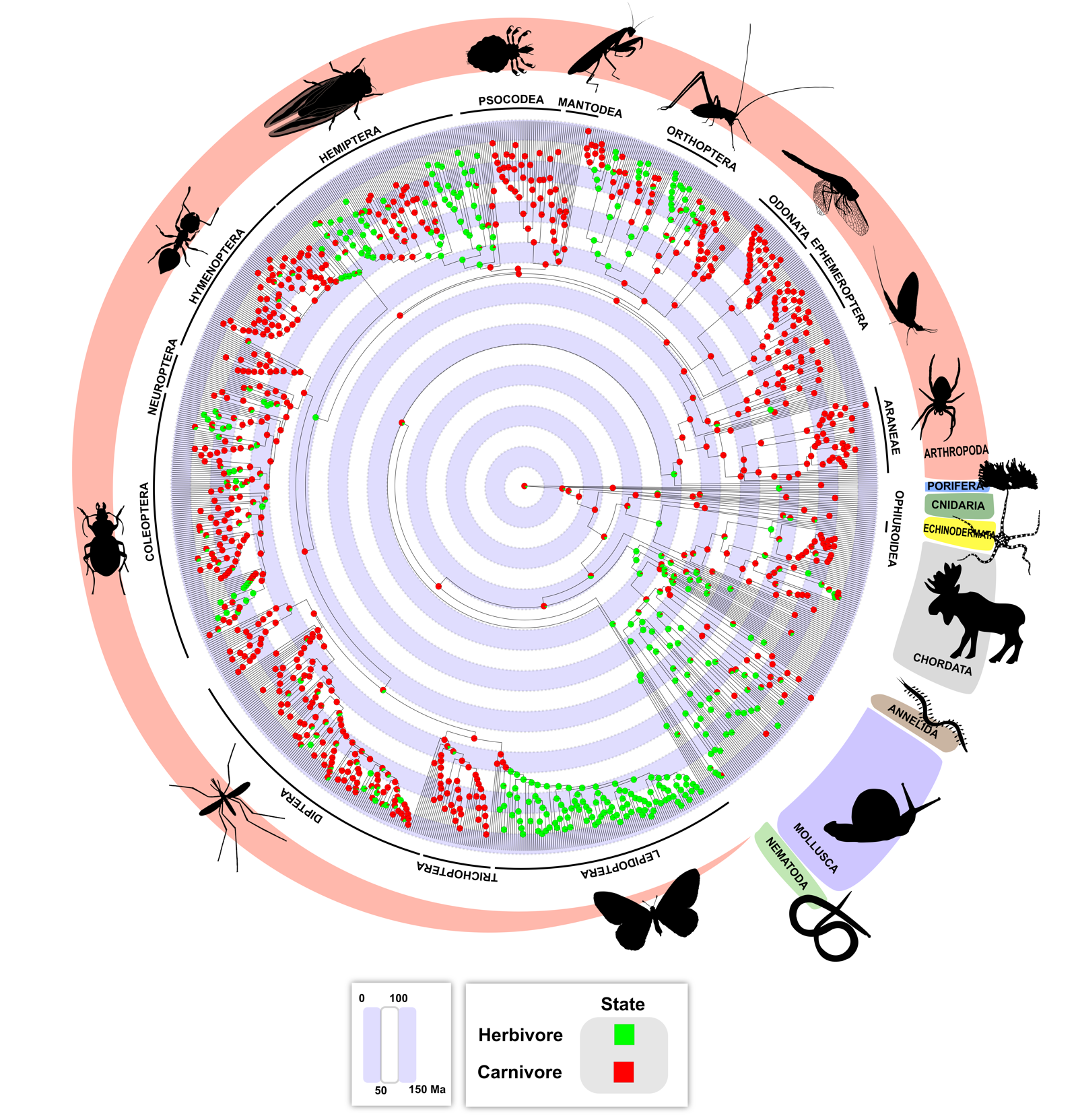
**

**Figure S5.** Ancestral-state reconstructions for Tree I for the M1 model fitted in BayesTraits using the maxcar coding strategy (treating omnivorous and ambiguous taxa as carnivorous). See Table S35–S36 for details.

**
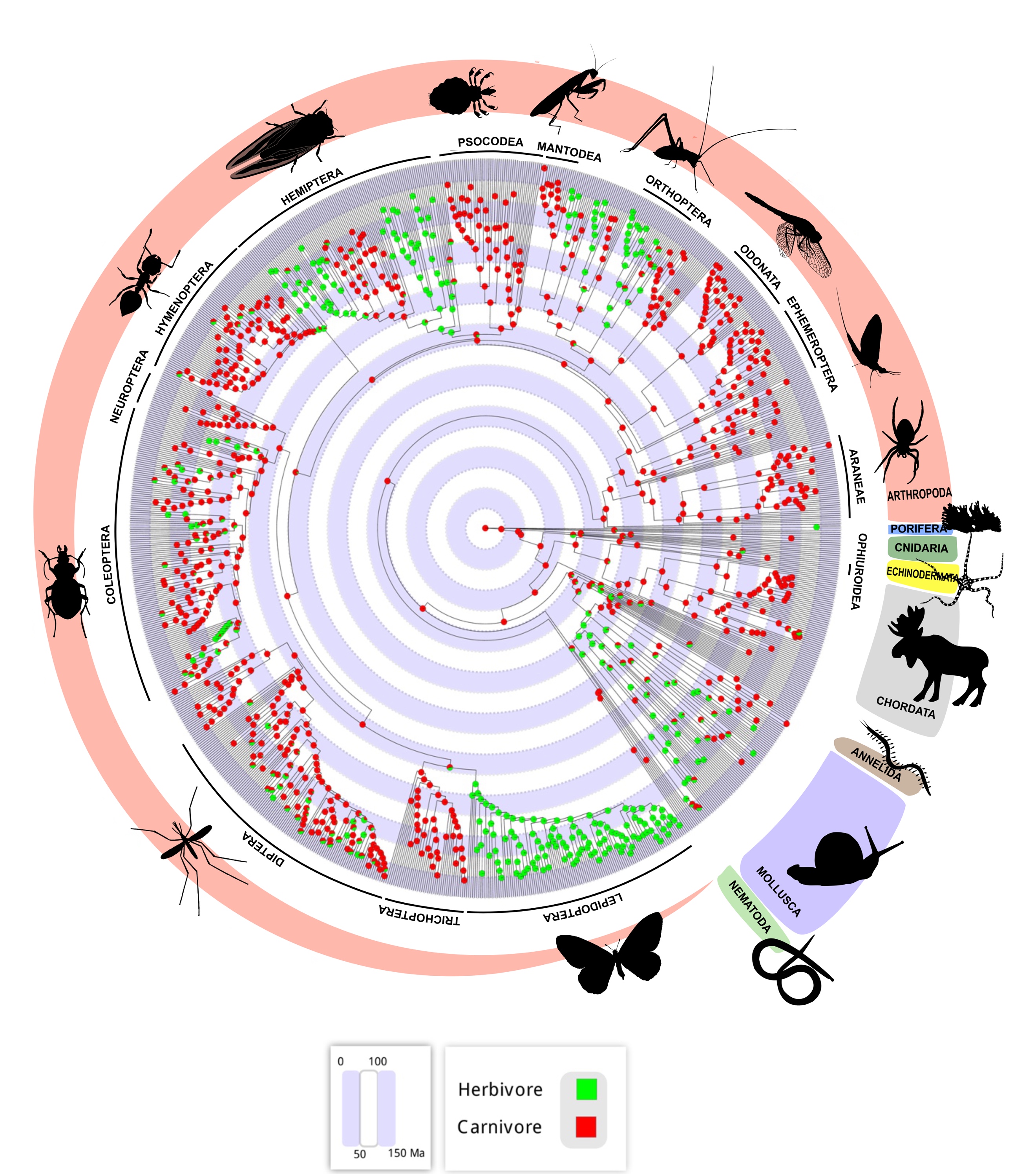
**

**Figure S6.** Ancestral-state reconstructions for Tree I for the M2 model fitted in BayesTraits using the maxcar coding strategy (treating omnivorous and ambiguous taxa as carnivorous). See Table S35–S36 for details.

**
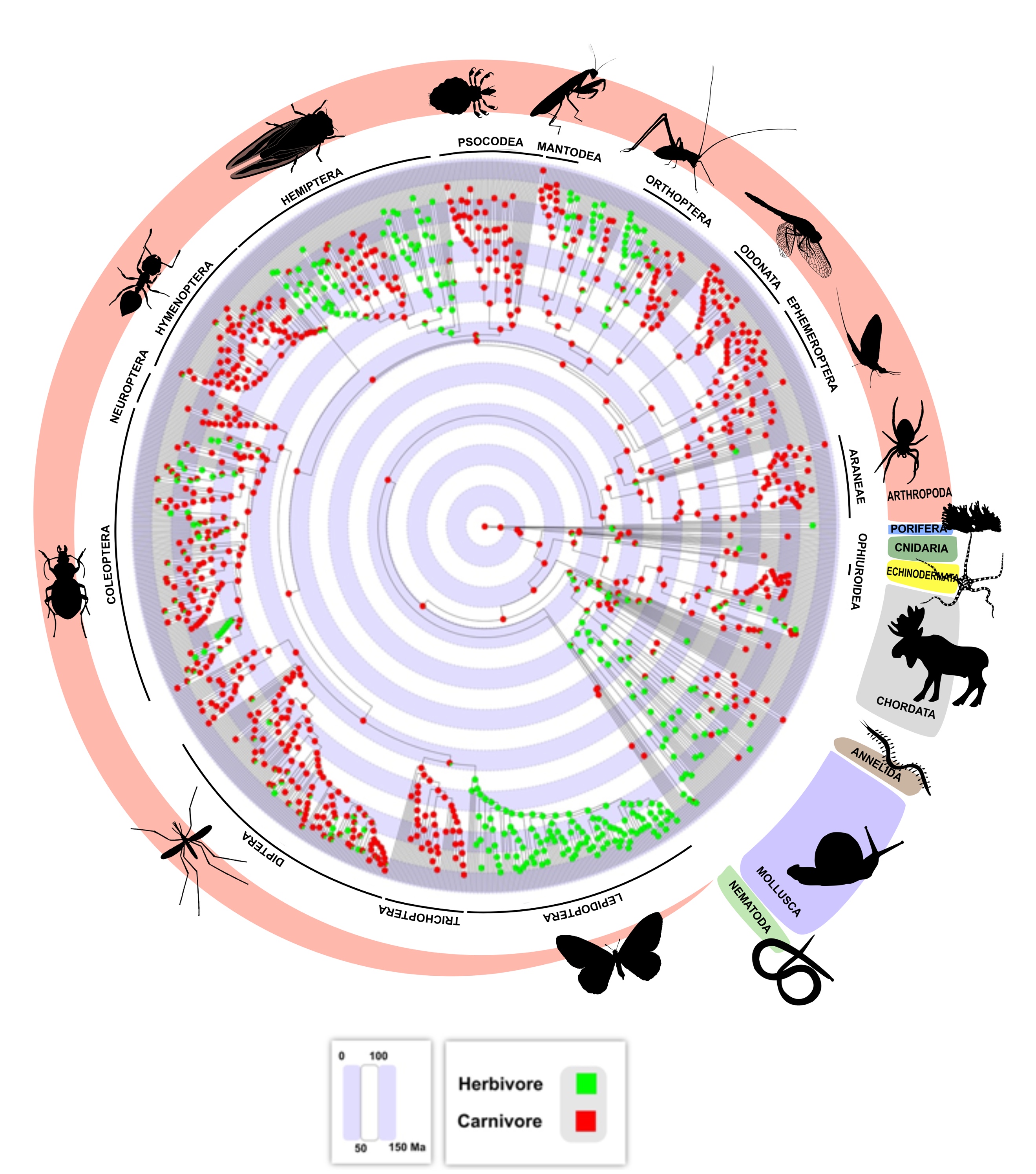
**

**Figure S7.** Ancestral-state reconstructions for Tree I for the M3 model fitted in BayesTraits using the maxcar coding strategy (treating omnivorous and ambiguous taxa as carnivorous). See Table S35–S36 for details.

**
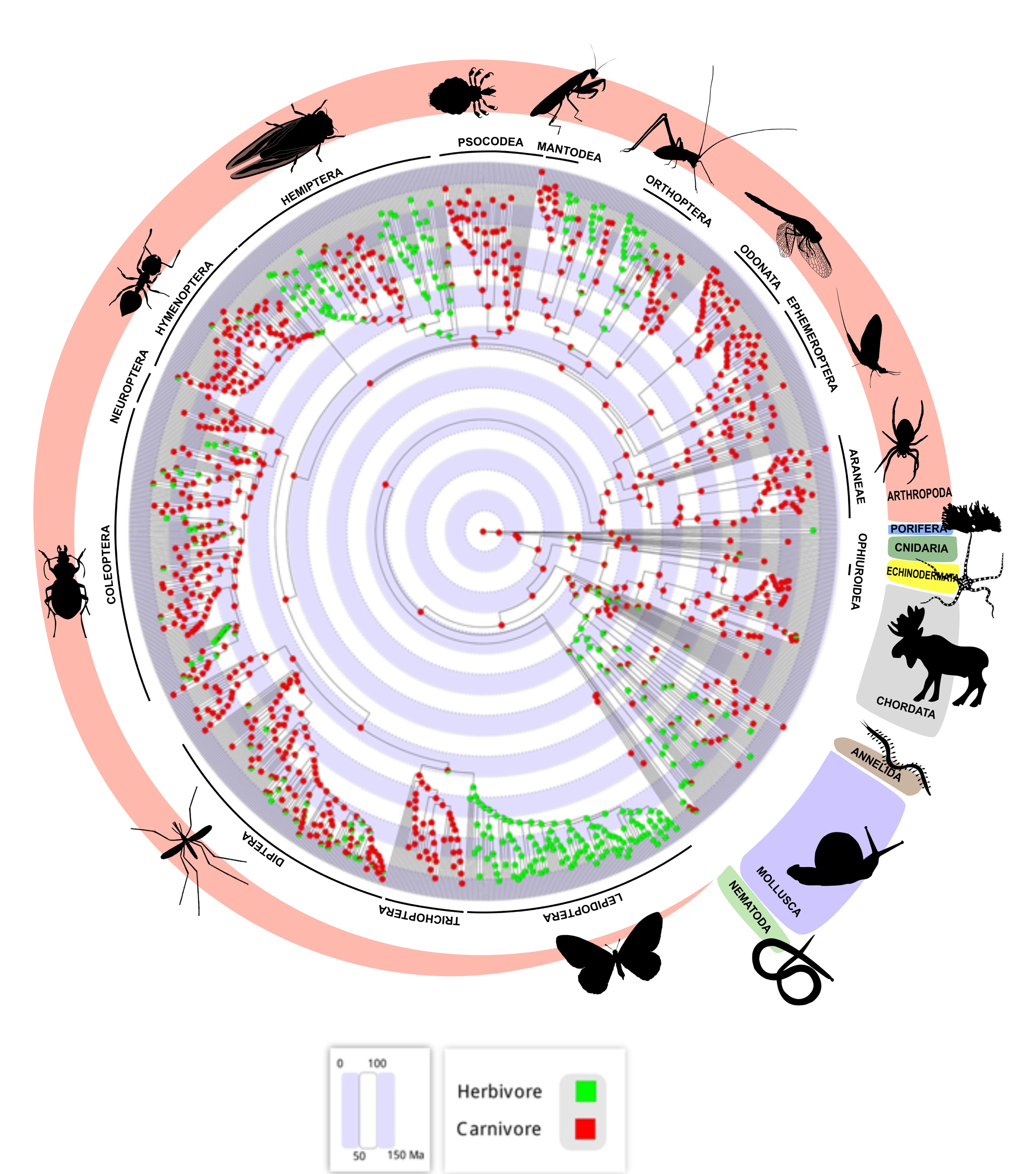
**

**Figure S8.** Ancestral-state reconstructions for Tree I for the M6 model fitted in BayesTraits using the maxcar coding strategy (treating omnivorous and ambiguous taxa as carnivorous). See Table S35–S36 for details.

**
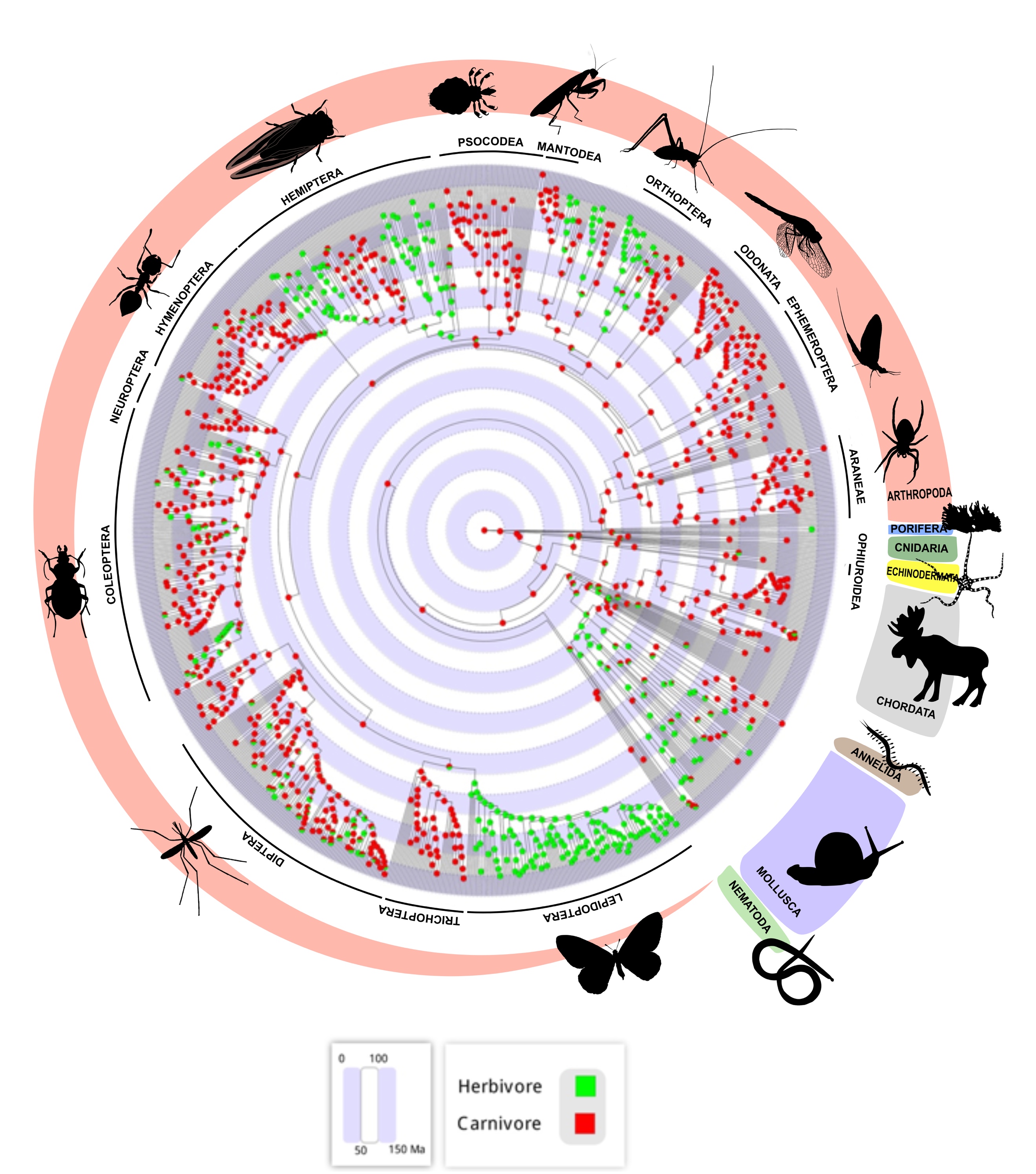
**

**Figure S9.** Ancestral-state reconstructions for Tree I for the M7 model fitted in BayesTraits using the maxcar coding strategy (treating omnivorous and ambiguous taxa as carnivorous). See Table S35–S36 for details.


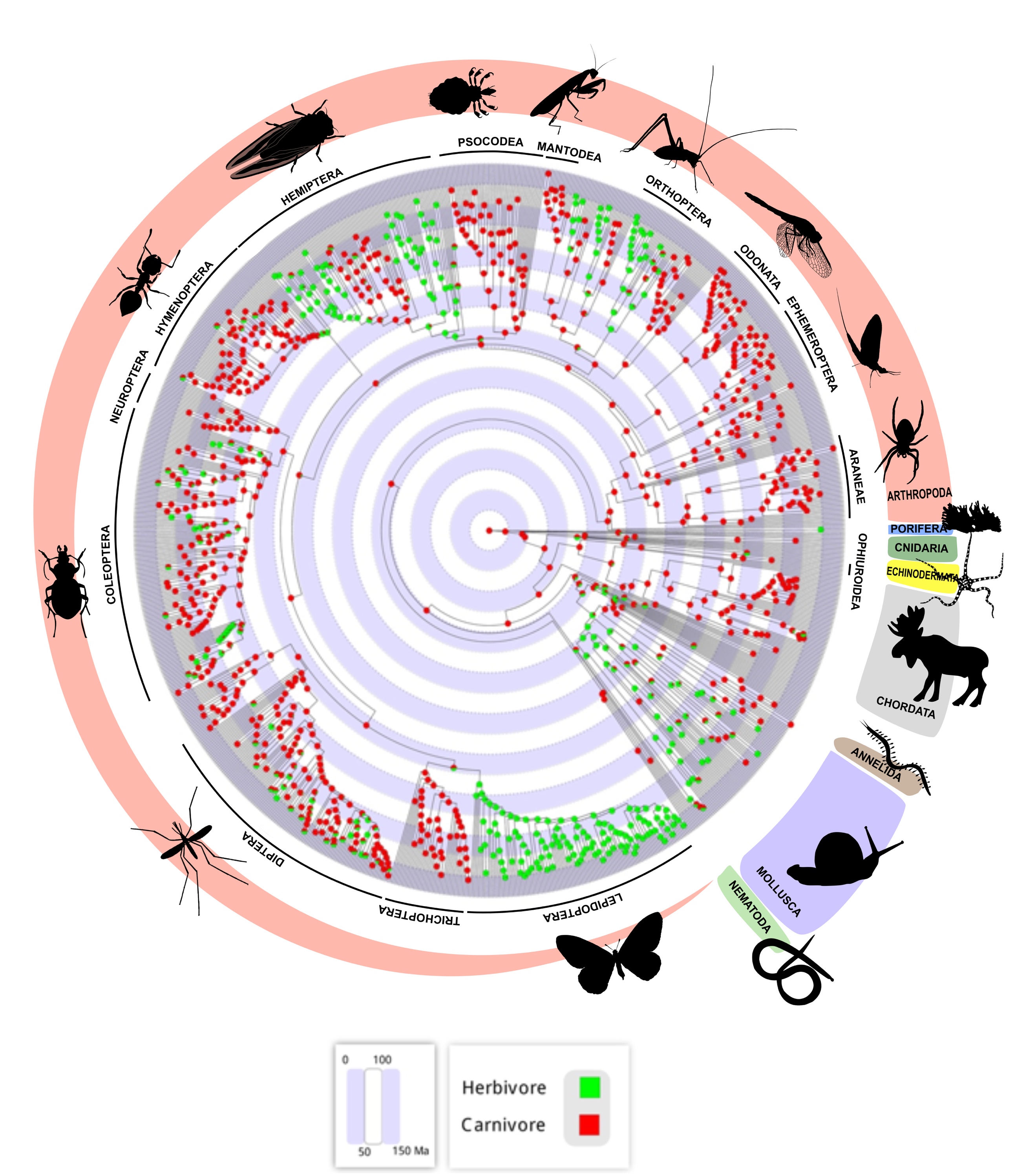

Supplement: Supplementary file 1 — Supplementary Material. Supplementary Methods, Tables S1–S49, Figures S1–S9. [file EVL3-3-339-s002.docx]
